# Early life microbiome disbalance impacts neuroendocrine outcomes in pre-pubertal mice in a sexually dimorphic manner

**DOI:** 10.3389/fmicb.2025.1504513

**Published:** 2025-06-20

**Authors:** Bistra B. Nankova, Furong Hu, Edmund F. LaGamma

**Affiliations:** ^1^Department of Pediatrics, New York Medical College, Valhalla, NY, United States; ^2^Department of Biochemistry and Molecular Biology, New York Medical College, Valhalla, NY, United States; ^3^The Regional Neonatal Center, Maria Fareri Children’s Hospital at Westchester Medical Center, Valhalla, NY, United States

**Keywords:** maternal antibiotics, neonatal microbiome, sex differences, behavior, stress response, catecholamine production/release, RNA sequencing, stimulus-secretion coupling

## Abstract

**Introduction:**

Adverse exposures during perinatal development disrupt the emerging gut microbial ecology that in turn negatively influences long term health. How gut dysbiosis affects complex neurobehavioral functions or even simple reflex arcs (e.g. the amplitude of sympathoadrenal adaptive responses to hypoglycemia) in the extrauterine environment is not well understood.

**Methods:**

The C57Bl6 dams were given broad-spectrum antibiotics in the drinking water at parturition until weaning of their litter to perturb the normal seeding and maturation of the postnatal microbiome, control animals received sterile water. To evaluate the impact of altered postnatal flora the offspring were subjected to behavioral tests or sacrificed after exposure to insulin-induced hypoglycemia. Fecal samples were collected for microbial whole genome shotgun taxonomic profiling and predictive functionality. As an index of host sympathoadrenal capacity, individual adrenal medulla samples from each group were subjected to RNA sequencing to identify differentially expressed genes between the groups and gain insights into molecular pathways contributing to the observed outcomes. Given that several neurodevelopmental disorders in humans are biased by sex we also included it as variable in this report.

**Results:**

The offspring of control dams displayed sex-specific differences in microbiome composition, exploratory behavior, adrenal transcriptome profiles and basal urinary epinephrine levels. Maternal antibiotics during nursing caused: (1) microbial dysbiosis in the offspring as evident by markedly enlarged ceca, no detectable by-products of bacterial fermentation (sp. SCFA) and dramatic changes in microbial composition, diversity (reduced - alpha Chao1and beta Bray-Curtis, as compared to their respective controls) and predictive metabolic activity; (2) alteration in the transcriptional signature of the adrenal medullae and attenuated peripheral stress responses in male offspring, associated with gap junction signaling pathways; (3) increased anxiety-like testing metrics, and decreased locomotor activity; all in a sexually dimorphic manner.

**Discussion:**

We speculate that the observed sex differences in the gut microbiome may contribute to neurodevelopmental disorders known to have sex-related disparities and in the capacity for successful adaptation to stress. A better understanding of how microbial communities and their hosts interact during critical portions of postnatal neurobehavioral development may help personalize nutritional and therapeutic strategies to promote long term health.

## 1 Introduction

The gut microbiota is a multifaceted ecosystem of microorganisms that co-evolved with their mammalian hosts over thousands of years in a symbiotic relationship where the interaction of genes and environment came to mediate digestion and nutrient absorption as well as to strengthen adaptive mucosal immune barrier defenses to contain luminal pathogens (Gilbert et al., 2018; Molina-Torres et al., 2019; Forsythe and Bienenstock, 2016). These relationships begin *in utero* when metabolites, antibodies and bioactive substrates produced by the maternal gut microbiota also support the formation of neural circuits, immune development, blood–brain barrier formation, programming of the hypothalamic–pituitary–adrenal (HPA) stress axis and regulation of appetite and energy balance in the developing offspring (Jasarevic and Bale, 2019). The newborn’s gut colonization became an advantage through evolution and commences after a vaginal birth upon passive transfer of maternal flora and her antibodies (Dominguez-Bello et al., 2010; Borre et al., 2014a; Mutic et al., 2017; Ratsika et al., 2021; Cryan et al., 2019; Bresesti et al., 2022; Kennedy et al., 2023), in parallel with ongoing neuronal maturation (Zoghbi, 2003) followed by the establishment of homeostasis between the gut microbiota and the mucosal GI immune system (Sanidad and Zeng, 2020). These pioneering neonatal bacteria prime the gut lumen for subsequent acquisition of secondary microbiota that further alters the maturing immune, metabolic, hormonal and nervous systems of the newborn (Arrieta et al., 2014; Diaz Heijtz et al., 2011; Staude et al., 2018; Dominguez-Bello, 2019; Smith et al., 2014).

During the perinatal period, an existing high rate of antibiotic use in both mother and newborn elevates the risk of disturbing the evolutionary appropriate homeostatic dynamic functions of the neonate’s microbiome thus, potentially altering the trajectory of the maturing brain and behavior (Klein et al., 2018). For example, maternal antibiotic use before and during pregnancy, or postpartum has been associated with increased risk for neurodevelopmental problems such as attention-deficit/hyperactivity disorder (ADHD), autism spectrum disorder (ASD), cerebral palsy (CP), and epilepsy in the offspring, as revealed by recent comprehensive systematic review and meta-analysis (Tao et al., 2022), and population-based cohort study (Hamad et al., 2019). Preclinical research also linked maternal antimicrobial exposure during pregnancy with reduced locomotor activity, higher anxiety-like behavior, reduced sociability (Tochitani et al., 2016; Leclercq et al., 2017; Kayyal et al., 2020), relevant to the etiology of ASD (Harshaw et al., 2022), as well as obesity, diabetes, inflammatory bowel disease and even immune-related afflictions like asthma and allergies in the adult offspring (Stokholm et al., 2014; Ramirez et al., 2020; Perdijk et al., 2024).

In tandem to the evolving microbiome’s impact on the CNS, the functional maturation of the stress-response systems occurs in the early postnatal period in parallel with the initial gut colonization and microbiota-gut-brain axis development to provide appropriate and coordinated physiological reactions essential for survival (Schmidt et al., 2003; Juruena et al., 2021). Using a well-characterized neurohumoral reflex arc as a model (sp. hypoglycemia-induced release of epinephrine from adrenal medullary chromaffin cells in response to activation of autonomic nervous system and splanchnic nerve stimulation), we recently reported that in adult male, germ-free mice (born and raised without microbiome, GF) or normally colonized mice with antibiotic-induced microbiome depletion, the adrenal catecholamine responses to hypoglycemic stress are selectively impaired (Giri et al., 2019; LaGamma et al., 2021). Considering the high rate of exposure to broad spectrum antibiotics prevailing as current standard of human perinatal care in mothers and/or their preterm human newborns, a better understanding of how interfering with the “normal” vertical microbial seeding of the newborn gut affects the ability to adapt to threatening environments (even before puberty), is a high priority. Moreover, if evident, this would serve as a compelling illustration of the existence of a direct connection between the microbiome and the emerging functions of the immature nervous system.

We hypothesized that interrupting the evolutionary established cycle of maternal-to-newborn microbiota transfer by exposure to antibiotics immediately after birth will affect the transcriptional output of the adrenal medullae (AM), the adaptive responses to hypoglycemic stress and modify more complex neurobehaviors in early postnatal life. Given that several neurodevelopmental disorders in humans are sex-biased (Fombonne, 2009; Jacquemont et al., 2014), we hypothesized that “sex” is an important biological variable in this process. Here we evaluated whether maternal oral antimicrobials during nursing will alter gut microbiome composition, diversity, and predictive functionality in their offspring and the interaction between sex, microbiome, behavior, stress responses and adrenal transcriptomes at weaning.

## 2 Methods

### 2.1 Animals

All experiments using C57Bl/6N mice were conducted in accordance with the ARRIVE guidelines and the National Institute of Health Guide for the Care and Use of Laboratory Animals. Protocols were approved by the NYMC Institutional Animal Care and Use Committee, including efforts to minimize suffering and the number of animals used.

#### 2.1.1 Breeding

**Figure 1:**
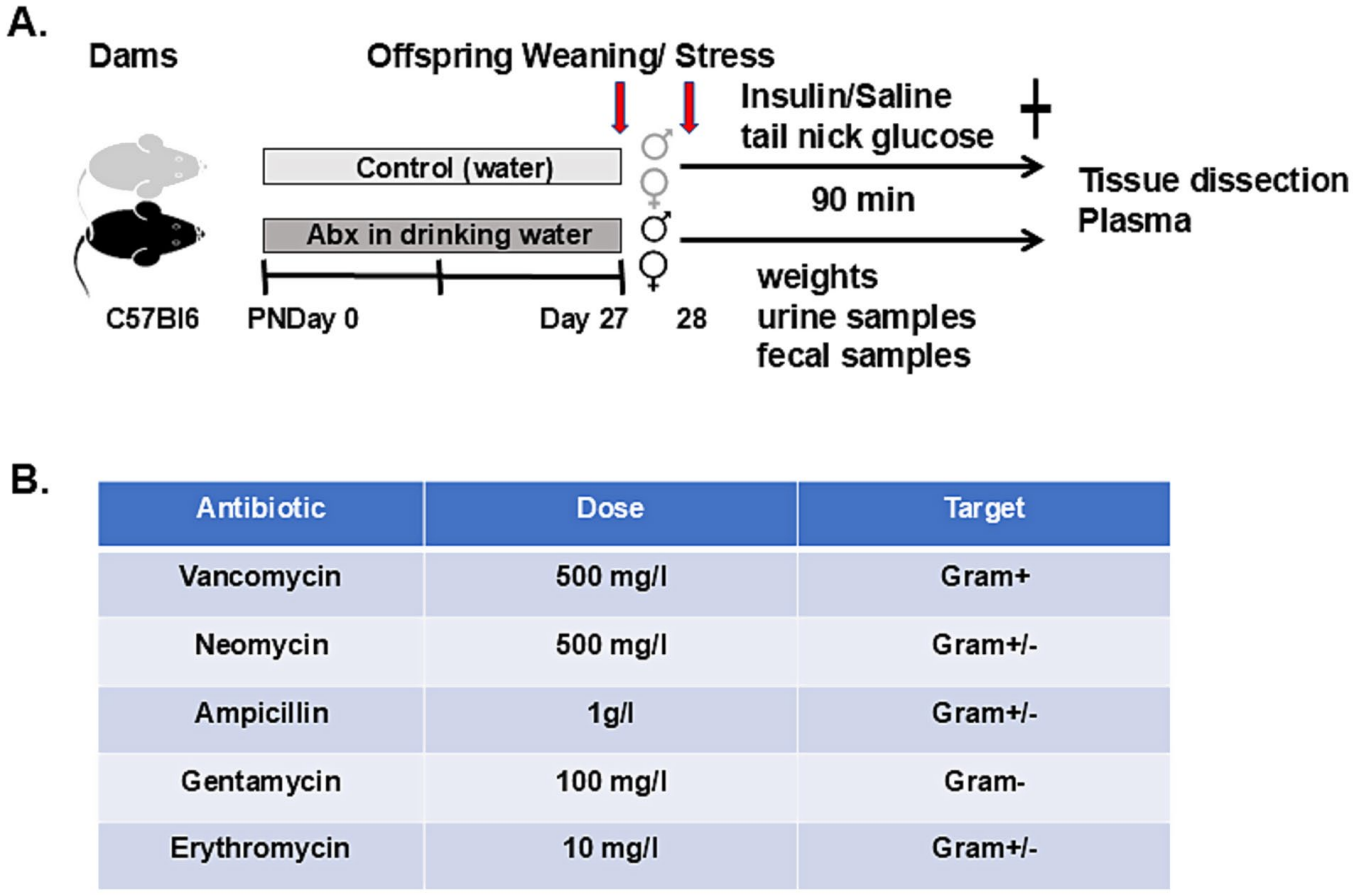
Graphical display of the experiments: **(A)** Dams were divided into 2 major groups: control (drinking regular sterile water) and Abx (mice given mixture of broad-spectrum antibiotics in the drinking water) starting from birth (PND 0). The offspring were weaned on PND 27, and the next day fasted for 3 h before the stress experiment. The pups (subdivided to male and female) were injected either with Insulin (2 IU/kg, i. p.) or Saline (equal volume). Blood glucose levels were monitored every 30 min (tail nick), and animals were sacrificed 90 min after injections. Fecal samples were collected aseptically for microbiome analyses before and at 30-, 60-, and 90-min time points. Urine samples were collected during blood glucose checks when possible. Plasma samples were collected at sacrifice and stored at −80°C for hormonal analyses. Adrenal medullae and ceca were collected, snap frozen and stored at −80°C for further analyses. **(B)** Oral antibiotics cocktail used in the study.

Male and female mice (7–8 weeks old) were purchased from Taconic Farms Inc., Germantown, NY, United States. The breeding strategy was polygamous mating (1 male and 2 females per cage). The animals were kept on a 12/12 h light/dark cycle, in a temperature (22–24^°^C) and hygrometry (70–80%) controlled room, with ad libitum access to water and food (LabDiet Laboratory Autoclavable Diet 5,010, St Louis, MO). The cages had plastic houses and nesting materials for enrichment. Weight gain for each female mice was monitored every second day to confirm pregnancy (Heyne et al., 2015). Close to delivery dams were transferred to a new cage. Cage changes were performed in a laminar flow hood. All litters were culled to 7–8 pups and weaned at postnatal week 4 (27 days) (Figure 1A).

#### 2.1.2 Antibiotics treatment

The antibiotic cocktail was composed of ampicillin (1 g/L; Sigma Aldrich, St. Louis, MO), vancomycin (0^.^5 g/L; Sagent Pharmaceuticals, Schaumburg, IL), neomycin (0^.^5 g/L; Fisher Scientific), gentamycin (100 mg/L; Sigma Aldrich), and erythromycin (10 mg/L; Sigma Aldrich) (Figure 1B) (see LaGamma et al., 2021; Reikvam et al., 2011; Kiraly et al., 2016; Sampson et al., 2016), and the red-colored drinking bottles (for protection from the light) were changed every second day. The water consumption of each dam was also monitored every 2nd day to estimate the antibiotics intake. Offspring from control and Abx-treated mice were weaned from their mothers at PND 27, pups were transferred to new cages and housed 2–5 (treatment and sex-matched) pups/cage with continuous respective treatments.

#### 2.1.3 Exposure to stress

For this part of the study, we used all pups from 12 dams: 6 Abx (45 weanling, 22 males and 23 females) and 6 control (43 pups including 22 males and 21 females). A total of two Abx dams were excluded from the study due to infanticide. At 4 weeks, the offspring were fasted for 3 h. with access to water (±Abx) prior to metabolic stress exposure. The groups were randomly subdivided to Insulin (Ins, receiving intraperitoneal injection of regular human insulin Humulin, Eli Lilly; 2UI/kg) or Saline (Sal, equal volume)—treated, as we and others described before (Shum et al., 2001; LaGamma et al., 2014; Kudrick et al., 2015; Kim et al., 2017; Giri et al., 2019; LaGamma et al., 2021). Blood glucose levels were monitored from the tail nicks before and every 30 min throughout the treatment using AlphaTrak3 Blood Glucose Monitoring System (Zoetis Inc., Kalamazoo, MI United States) (see experimental design, Figure 1A).

Mice were sacrificed after 90 min with an overdose of ketamine (35 mg/kg)—xylazine (5 mg/kg) cocktail. To avoid fluctuations due to the host circadian rhythm, all samples were collected at the same time of the day. Blood was collected by cardiac puncture prior to the excision of tissues. Tissue samples (ceca and adrenal medullae) were dissected, snap-frozen and stored at -80°C for further molecular analysis.

#### 2.1.4 Fecal and urine samples

Fecal pellets were collected aseptically from individual mice in each litter on their corresponding postnatal day 28 during the stress exposure (before and at 30-, 60-, and 90-min time points when glucose check was performed). To limit potential influences of diurnal compositional and functional oscillations of the microbiome all samples were collected between 10 a.m. and 2 p.m. and were stored at −80^°^C until further use (Thaiss et al., 2014; Wang et al., 2018; Mukherji et al., 2013; Kuang et al., 2019). For whole genome shotgun sequence analysis samples from each litter were pooled (females and males separate) to assure sufficient amount of extracted DNA.

Urine samples for catecholamine detection were collected, when possible, on PND 28 before and every 30 min after injection (Ins or Sal).

#### 2.1.5 Behavioral studies

**Figure 2:**
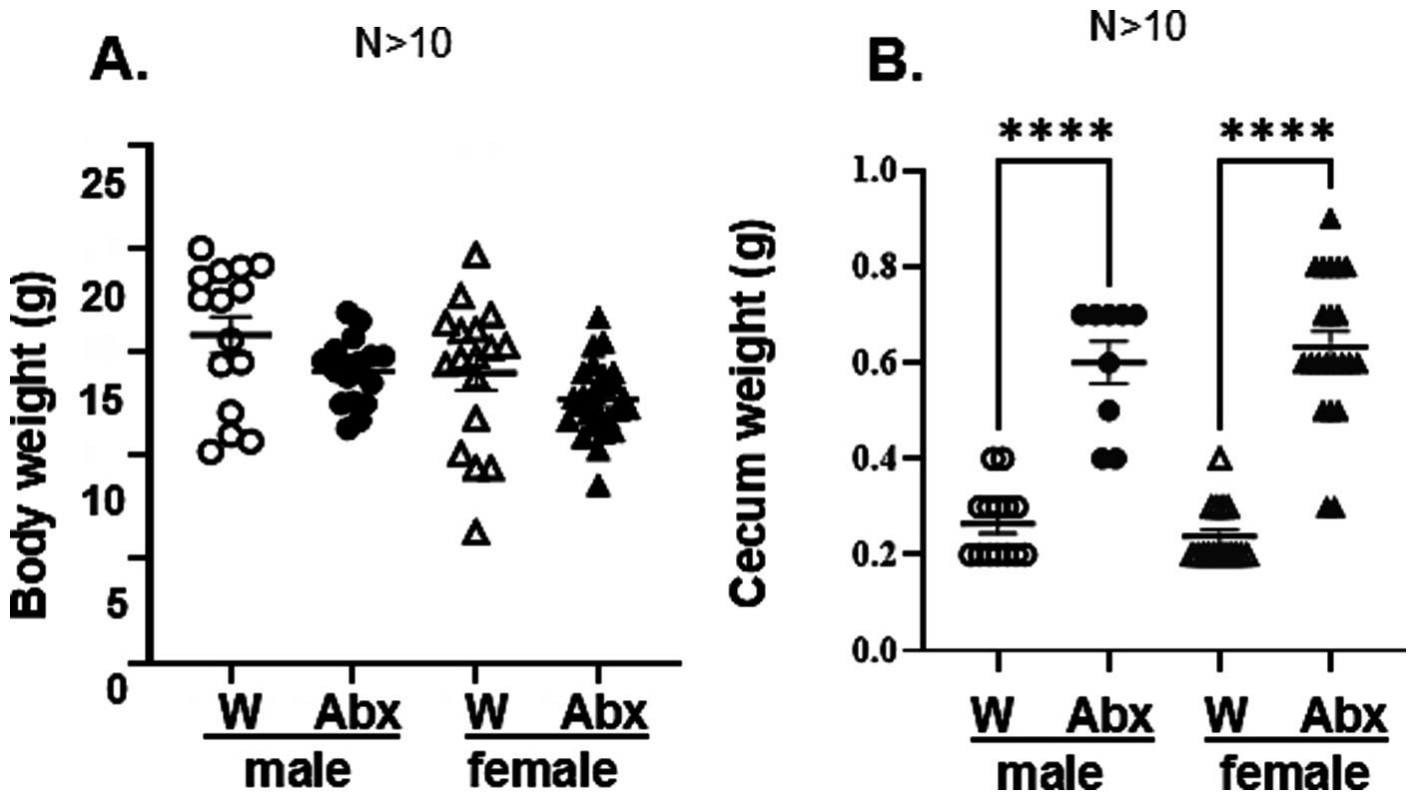
Maternal exposure to Abx did not affect basal physiology in the offspring, but enlarged ceca: **(A)** Body weights in the offspring were recorded before the stress experiment at 4 weeks: Data passed the normality test and were analyzed using one way-ANOVA, followed by Tukey’s multiple comparisons test. All data are expressed as means ± SEM, ^**^p < 0^.^002. Each dot represents value for an individual animal, *N* > 10/group. **(B)** Each caecum was dissected after sacrifice and weighted using precision scale (*N* > 10). Data passed the normality test and were analyzed using one way-ANOVA, followed by Tukey’s multiple comparisons test. All data are expressed as means ± SEM, ^****^p ≤ 0^.^0001. Each dot represents value for an individual animal.

In a separate set of experiments, behavioral profiling was performed 1 day after weaning the offsprings of control dams (drinking water, 3 dams with 21 weanlings, 8 males and 13 females) and Abx-exposed dams (receiving cocktail of antibiotics in the drinking water, 4 dams with 29 pups, 15 males and 14 females) as described (see Figure 2A). Pups were divided into separate cages by sex and treatment and received corresponding water bottles. On the day of testing, animals were transported to the testing room and left undisturbed for 30 min before the test. All behavioral tests were performed in a room with dim light between 8 a.m. and 12:30 p.m. and videotaped with a ceiling camera and analyzed by ANY-maze 5.14 tracking system software (Stoelting Co, Wood Dale, Illinois). Males and females were tested separately; males first, then followed by females, for 5 min. The Open Field (OF) box had dimensions 45 × 45 × 20 cm (L × W × H), and the total distance traveled and the time and number of entries into the center of the OF arena were calculated. Anxiety/avoidance-like behavior was tested on the Elevated Plus Maze (EPM). The apparatus (Stoelting, Wood Dale, IL, United States), 74 cm above ground level, had four cross-shaped platforms (48 × 12 cm); two platforms with a 2-cm-high plexiglass fence wall were open, while the other two platforms with 42-cm-high opaque walls on the sides were closed. Arms of the same type are located opposite each other. The animals were acclimatized to the room for 30 min prior to each experiment. Each pup was placed on the central platform with its head toward an open arm and allowed 5 min to explore the maze. Their movements on the maze were tracked. The maze was then cleaned with 70% ethanol between tests. The following measurements were calculated: % duration in the open arm (OA)—calculated as a percentage of the total time of the test; and % entries into the OA—calculated as the percentage of total open arm entries of the total number of arm entries. All tests were analyzed by trained individuals blinded to the group assignments.

### 2.2 Analytical methods

#### 2.2.1 Urinary epinephrine analysis

Urine samples were acidified immediately after collection with an equal volume of 0^.^01 M HCl and stored at −80°C for further analysis (Wang et al., 2016). The levels of epinephrine were quantified (in duplicates) using competitive enzyme immunoassay kit (Rocky Mountain Diagnostics, Colorado Springs, CO) and normalized to creatinine concentrations in the same samples (DetectX Urinary Creatinine Kit, Arbor Assays, Ann Arbor, MI) as described earlier (Giri et al., 2019; LaGamma et al., 2021).

#### 2.2.2 Plasma hormones measurements

All samples for hormonal assays were measured in duplicates using commercially available immunoassay kits: Arbor Assays, Ann Arbor, MI for corticosterone and Millipore Sigma, Burlington MA for glucagon, resp.

#### 2.2.3 SCFA analysis

Cecal SCFA gas chromatography analysis was performed at the Gnotobiotics, Microbiology and Metagenomics Center (Boston, MA) using an Agilent 7890B system with a flame ionization detector (FID, Agilent Technologies, Santa Clara, CA) as described before (Giri et al., 2019; LaGamma et al., 2021). Briefly, the chromatographic analysis was carried out using a high-resolution gas chromatography capillary column 30 m × 0^.^25 mm coated with 0^.^25um film thickness was used (DB-FFAP) for the volatile acids (Agilent Technologies). Nitrogen was used as the carrier gas. The oven temperature was 145°C and the FID and injection port were set to 225°C. The injected sample volume was 1 μL and the run time for each analysis was 12 min. Chromatograms and data integration were carried out using the OpenLabChemStation software (Agilent Technologies). A volatile acid mix containing 10 mM of acetic, propionic, isobutyric, butyric, isovaleric, valeric, isocaproic, caproic and heptanoic acids was used for standard solution (Supelco CRM46975, Bellefonte, PA). An internal standard control (stock solution containing 1% 2-methyl pentanoic acid, Sigma-Aldrich St. Louis, MO) was used for the volatile acid extractions.

### 2.3 RNA sequencing and analysis

RNA was isolated from the dissected adrenal medullae (3 randomly selected biological replicates per group, originating from different litters) using RNeasy MicroKit (Qiagen) according to the manufacturer’s instructions. More than 200 ng of total RNA from each sample were submitted to Yale Center for Genome Analysis (YCGA) for PolyA RNA-seq analysis service including alignment, expression counting, differential gene expression and pathway analysis of the differentially expressed genes. Briefly, sample’s quality was determined using an Agilent TapeStation (Agilent Technologies, Santa Clara, CA) to assure that their RNA integrity numbers were greater than 7.0. PolyA RNA library preparation and sequencing on NovaSeq X Plus using 2 × 150 paired-end read configuration to a minimum depth of 25 milllion reads per sample were performed according to standard procedures, including FASTQC and quality control of aligned reads. Differentially expressed genes (DEG) were analyzed in R using the DESeq2 (Love et al., 2014). Significance was defined to be those with *q*-value <0.05 calculated by the Benjamini-Hochberg method to control the false discovery rate (FDR). Comparison analysis of DEGs was performed between the treatment groups—Male W vs. Male Abx and Female W vs. Female Abx separately; and between the sexes—Female W vs. Male W and Female Abx vs. Male Abx.

### 2.4 Microbiome analyses

DNA from pooled stool samples (*n* = 6 per group, originating from different litters) was isolated using DNeasy Power SoilPro Kit according to the manufacturer’s protocols (Qiagen). The purity (260/280 ratio) and concentration of the extracted DNA were measured using a NanoDrop spectrophotometer (NanoDrop Technologies, Wilmington, Delaware, United States). DNA samples (*n* = 6/group) were sent to CosmosID Inc., Rockville, MD, United States for library preparation (using Illumina Nextera XT Library Preparation Kit, Illumina, San Diego, CA, United States), whole genome shotgun (WGS) sequencing (3 million total reads, 2×150bp), and taxonomic and predictive functional profiling of the gut microbiota.

### 2.5 Bioinformatics and statistical analyses

Unassembled reads were directly analyzed by CosmosID-HUB bioinformatics platform (CosmosID Inc., Rockville MD) for multi-kingdom microbiome analysis, profiling of antibiotic resistance and virulence genes and quantification of organism’s relative abundance (Ranjan et al., 2016). Shifts in microbial composition were assessed in relation to offspring’s sex and treatment group. Alpha and beta diversity were calculated from the species level relative abundance matrices from CosmosID taxonomic analysis using the R software Vegan package (Version 2.5-6). Wilcoxon Rank-Sum tests were done to investigate the statistical difference in the alpha diversity of the gut microbiota based on the Chao1, Simpson and Shannon indexes between the groups using the ggsignif package for R. Boxplots were generated using the same package for R. The statistical significance of the beta diversity between groups was determined with the nonparametric PERMANOVA (Permutational multivariate of variance analysis) based on the Bray–Curtis’s distance using Vegan’s function adonis2. The principal coordinate analysis plot was generated using the Vegan package’s PCoA function. Plots were visualized using the ggpubr package for R. Nonsignificant differences are not indicated in the figures.

Predictive microbial functional profiles and functional enrichment analysis and visualization plots were generated using CosmosID-HUB application and MetaCyc database (Caspi et al., 2016, web application).^1^ Briefly, the quality-controlled reads are subjected to a translated search against a comprehensive and non-redundant protein sequence database, UniRef 90, provided by UniProt (Apweiler et al., 2004). The mapping of metagenomic reads to gene sequences are weighted by mapping quality, coverage, and gene sequence length to estimate community wide weighted gene family abundances as described (Franzosa et al., 2018). Gene families are then annotated to MetaCyc reactions (Metabolic Enzymes) to reconstruct and quantify MetaCyc (Caspi et al., 2016) metabolic pathways in the community as described (Franzosa et al., 2018). Furthermore, the UniRef_90 gene families are also regrouped to GO terms (Carbon et al., 2009) to get an overview of GO functions in the community (data not shown).

Differences in the relative abundance of microbial features were determined by linear discriminant analysis (LDA) effect size (LEfSe) using the CosmosID-HUB. A logarithmic LDA score (log10) ≥ 2.0 was used as a threshold for nonparametric factorial Kruskal-Wallis test (alpha value of 0.05), and a Wilcoxon alpha value of 0.05 which were used to judge whether a test statistic was statistically significant when comparing the groups. LEfSe figures were generated using the LEfSe tool from the Huttenhower lab, based on the phylum, genus, species, strain, and functional matrices from the CosmosID analysis.

### 2.6 Data analyses

Statistical analysis was performed using the GraphPad Prism 9 (GraphPad Software Inc.). The normality of all quantitative variables (Shapiro–Wilk test), and the equality of variances (Brown-Forsythe and Bartlett’s tests) were tested before the analysis. Comparisons of the groups were performed using One-Way ANOVA followed by Tukey’s multiple comparison test for Gaussian distributions, whereas the Kruskal-Wallis test followed by Dunn’s multiple comparison test was used for non-Gaussian distributions. Two-way ANOVA were used when appropriate, with post-hoc Sídăk’s and/or Tukey’s multiple comparisons test. A *p*-value of <0^.^05 was used to indicate statistically significant differences. Data are expressed as the mean ± SEM.

The whole genome shotgun sequencing data is deposited to NCBI SRA (BioProject ID PRJNA1098026).

## 3 Results

### 3.1 Maternal antimicrobials during nursing did not affect offspring’s baseline physiology

On postnatal day (PND) 0 the dams were randomly assigned to two main groups: control (C) and experimental (mice exposed to broad spectrum antibiotics, Abx). To avoid any confounding effects resulting from chronic stress induced by oral gavage (Branchi et al., 2005) the cocktail of broad spectrum Abx was administered in the dam’s drinking water (see experimental designs in Figure 1). Controls received regular sterile water. This protocol was shown previously to substantially deplete the gut microbiota in adult mice with minimal off-target effects due to systemic absorption (LaGamma et al., 2021; Reikvam et al., 2011; Kiraly et al., 2016; Sampson et al., 2016). Mothers’ weight and water consumption were monitored until weaning (PND 27). Since water consumption increases significantly during lactating periods and is influenced by the number of pups per litter, the doses of Abx were sustained by adjusting them to body weight and water consumption of dams, measured every second day. Given the potential link between maternal psychosocial distress and milk microbiome (Ortega et al., 2021; Gur et al., 2017; Browne et al., 2019) litters with dam’s signs of discomfort (barbarizing pups) were excluded from the study. Moms exposed to oral antimicrobials were sacrificed to confirm the effectiveness of the protocol and as expected showed macroscopical signs of dysbiosis (significantly enlarged ceca, data not shown), as reported by others (Nyangahu et al., 2018).

**Figure 3:**
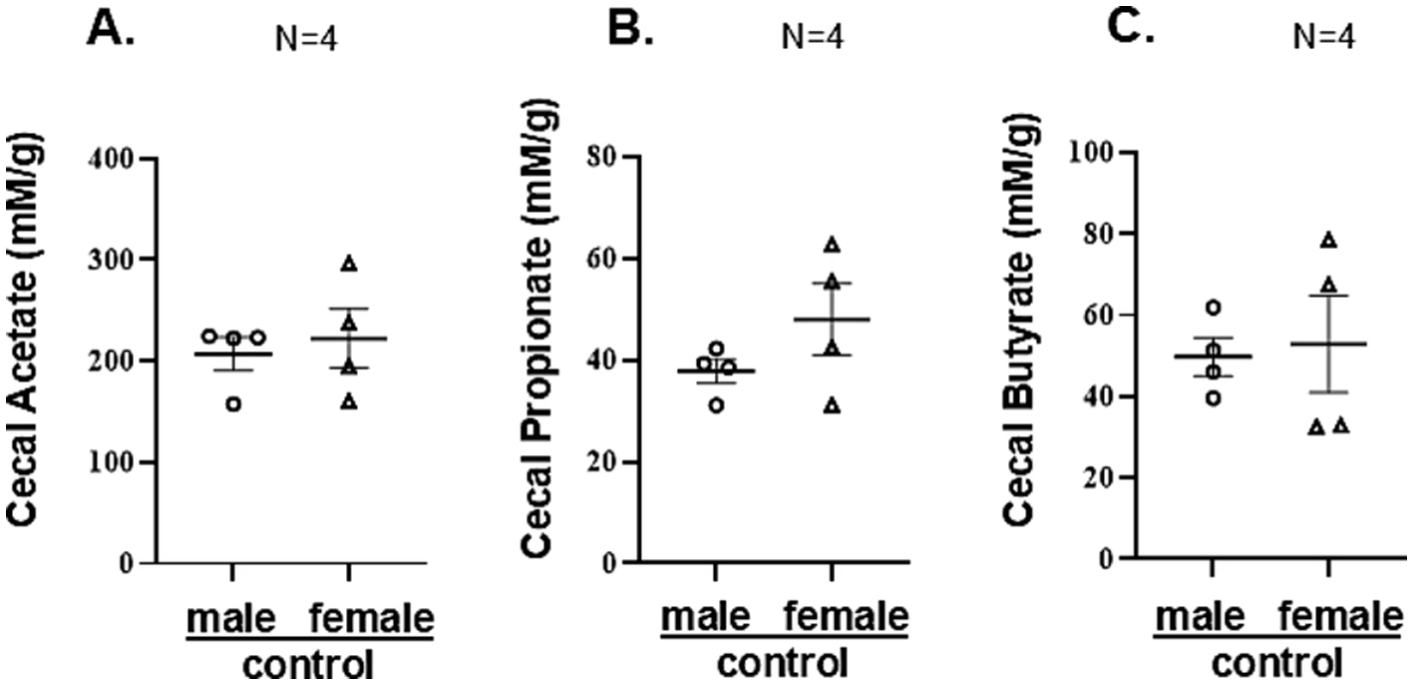
The offspring of mice on oral antimicrobials had no detectable SCFA: **(A–C)** Cecal SCFAs (mM/g tissue) from randomly selected individual samples (n = 4) for each group (originating from different litters) were quantified by gas chromatography analysis as described in Methods. They were not detectable in the Abx groups. Data for male and female controls were summarized for each experimental group and compared between groups by unpaired two-tailed t-test. No significant sex differences were found.

Bodyweights of the offspring were recorded on the day of stress (PND 28) as a measure of general wellbeing (Figure 2A). No significant differences were observed between treatment and control experimental groups and between sexes. Post-mortem cecal weights in the offspring of mice drinking Abx water were also significantly higher when compared to controls [hallmark of microbiome disruption (Reikvam et al., 2011; Kiraly et al., 2016; Simpson et al., 2020; Erny et al., 2015; Smith et al., 2007; Figure 2B)]. No sex differences between the groups were observed. In addition, by-products of bacterial fermentation (cecal SCFA) were not detectable in the Abx groups, consistent with microbiome depletion. In the offspring of control dams (drinking regular water) we did not find significant differences in the cecal SCFA levels between male and female pups on PND 28 (Figures 3A–C), although data for females varied in a larger range.

Given that cecal SCFA are index of bacterial metabolic activity, in a separate pilot experiment we pooled cecal samples from male and female control offspring (moms on regular water) on several postnatal days (from 3rd to 16th) and analyzed their SCFA content to determine if the initial gut colonization occur with the same dynamics for male and female pups. While SCFA were not detected between PND 3–11, on PND 14 acetate and butyrate were detected only in male pups (Supplementary Figure 1A). On PND 16 both, male and female offspring had detectable cecal SCFA, although in different concentrations (lower for acetate and propionate in females, Supplementary Figure 1B), suggesting delayed initial colonization and different microbial activity in females. Of note, SCFA levels at PND 16 were significantly lower than the levels detected at PND 28 (compared to the values reported in Figures 3A–C).

### 3.2 Antimicrobial treatment of mothers reshapes the bacterial communities in the offspring

**Figure 4:**
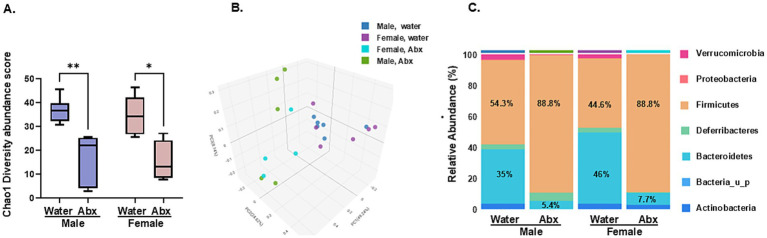
Maternal oral Abx during nursing profoundly shifted gut microbial composition in the offspring: The WGS sequencing was used to determine the microbial diversity and composition in stool samples of male and female pups born to dams drinking regular water or Abx cocktail (*n* ≥ 4 per group, samples originating from different litters). Taxonomic profiling was performed using CosmosID-Hub. **(A)** Alpha diversity represented by Chao 1 index (richness). Boxplots show 25th and 75th percentiles with a line at the median. Data were analyzed using Wilcoxon rank sum test for biological consistence, ^**^p = 0^.^006; ^*^p = 0^.^025. **(B)** Beta diversity (Bray-Curtis) principal coordinate analysis: *F*-value was calculated based on permutation-based ANOVA (PERMANOVA) test from the Vegan package, all ^**^p = 0^.^001. **(C)** Stacked bar (aggregated by group) based on % relative abundance (phylum level).

Based on the macroscopic indication of commensals depletion in the offspring of Abx dams and the lack of detectable SCFA, we next analyzed microbiome composition and predictive functionality in pooled stool samples from each experimental group, using WGS sequencing, allowing for deep resolution of microbiome communities, down to strain level. Comparison analyses revealed that both, female and male weanling of Abx-treated dams displayed significantly altered taxonomic profiles and diversity compared to their respective control groups (nursed by mothers drinking regular water). For example, alpha diversity Chao1 index of microbial richness within each group (known to give more weight to rare species) was different between the controls and the Abx offspring (Figure 4A), with significantly decreased number of species displayed in Abx groups [for both, male (^**^p value 0^.^006) and females (^*^p value 0^.^025), Wilcoxon Rank Sum test]. Of note, no significant differences were observed between controls (male vs. female) and between treatment groups (male Abx vs. female Abx) respectively, suggesting no major sex-specific shifts in microbiome composition. In the Simpson (gives more weight to common or dominant species) and Shannon (accounts for both richness and evenness) indexes only male Abx were significantly different from male controls (^*^p values 0^.^045), data not shown. Also, principal coordinate analysis revealed significant differences in beta diversity (which provides a measure of similarity or dissimilarity of the whole microbial community between the groups), as shown in Figure 4B (Bray Curtis distances). There was apparent separation between the samples from offspring of Abx and control mice [p values 0^.^008 (male control vs. male Abx), and 0^.^004 (female control vs. female Abx) resp., PERMANOVA analysis], demonstrating significant differences in their respective gut microbiome structure. No separation between males and females both, in control or Abx groups was observed.

The taxonomic profiles are illustrated in Figure 4C—stacked bar, phylum level (top 25—most abundant, see also detailed strain level relative abundances in Supplementary Table 1). Firmicutes (now Bacillota, with total of 67 strains detected) was the most abundant phylum detected in fecal microbiotas in control males (mean relative abundance 54^.^3%), and second most abundant for control females (44^.^6%). This phylum was significantly increased in Abx offspring to 88^.^8% (for both, Abx male and Abx female). In contrast, Bacteroidota (formerly known as Bacteroidetes, total of 43 strains detected) was greatly decreased in the Abx groups to 5^.^4% (from 35% in male controls, 6^.^5-fold decrease) and 7^.^7% (from 46% in female controls, 6-fold decrease, resp). Phylum Actinobacteria had similar relative abundance in male and female controls (3^.^48 and 3^.^54% resp., 11 strains), and Abx in maternal drinking water resulted in no detectable levels in males and reduced relative abundance in females (2^.^8%). Phylum Verrucomicrobia (3 strains) was significantly decreased in Abx male offspring (from 3^.^3 to 0^.^33%) and in Abx female offspring (from 2^.^5% in control group to no detectable levels). Similar for phylum Proteobacteria (3 strains), pups born to dam’s drinking Abx water had reduced relative abundance (from 0^.^5 to 0^.^23% in males, and from 0^.^35% to no detectable levels in females). The only sex difference in microbial composition observed at phylum level was the opposite effect of maternal antimicrobials on Deferibacteres (1 strain, *Mucispirillum schaedleri ASF457*) causing increased relative abundance in male Abx offspring (from 3^.^3 to 5^.^2%) but decreased relative abundance in female Abx offspring (from 3^.^0 to 0^.^5%). Thus, the maternal oral Abx caused significant shifts in the offspring’s microbial composition, consistent with the observed lack of detectable SCFA.

**Figure 5:**
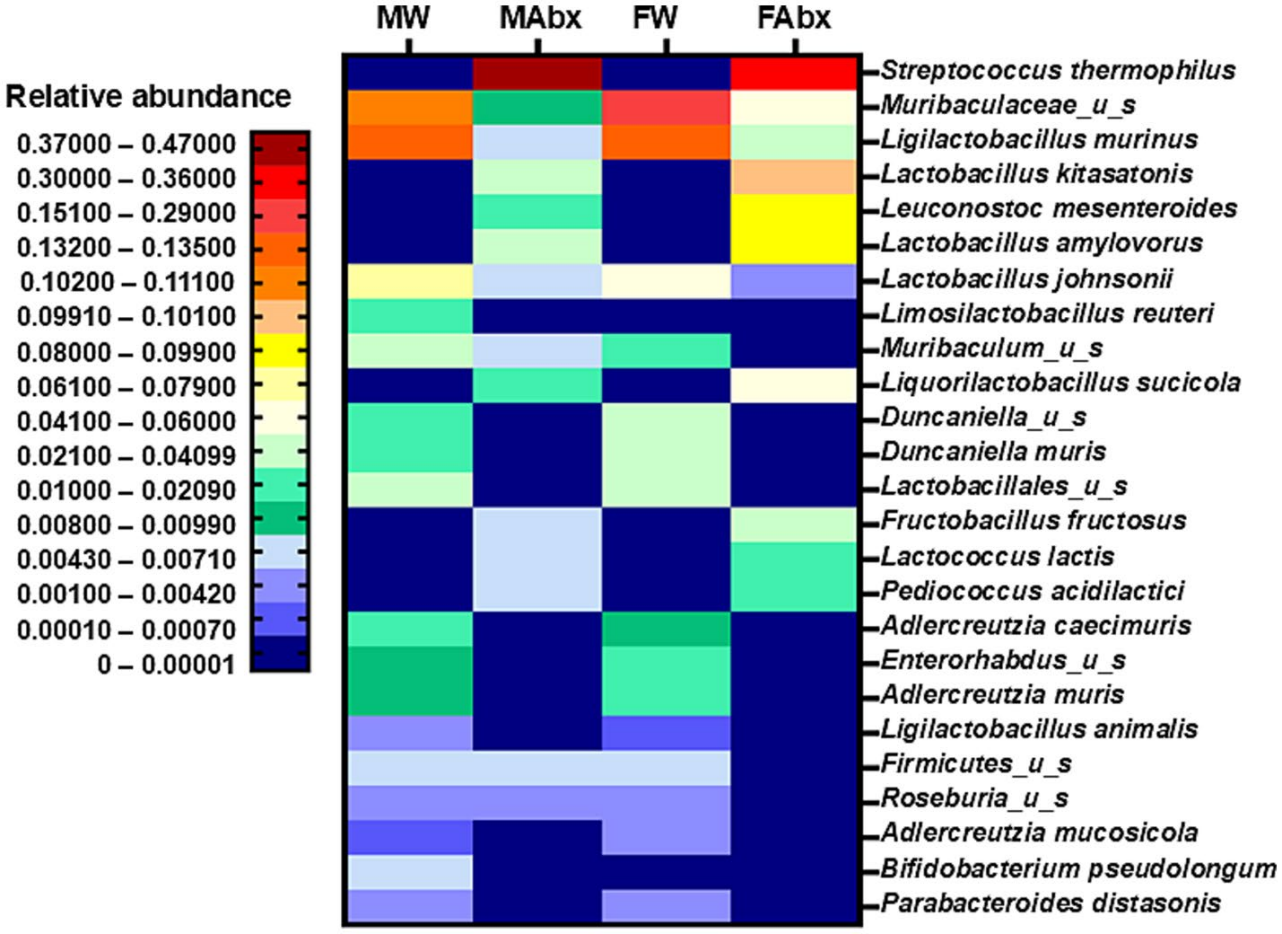
Heatmap illustrating significantly different microbial strains found between the groups: based on WGS sequencing of individual fecal samples, taxonomic profiling and comparative analysis (CosmosID), top 25 most abundant strains (mean values) shown.

Next, we performed Linear Discriminant Analysis Effect Size (LEfSe) for multilevel comparison of taxa across the experimental groups using the default statistical thresholds as described (Segata et al., 2011). The results are illustrated on the graphic plot of LDA scores [Supplementary Figure 2, indicating most enriched taxa in each group (LDA threshold 3^.^47 to 5^.^35, *p*-values < 0^.^001 to 0^.^041; total of 25) see Supplementary Table 2 for details]. The treatment and sex-specific strain differences in microbial composition between the groups are visualized on the heat map (Figure 5). More specifically, the comparison analysis revealed nine taxa detected only in controls (both, male and female), namely *Parabacterioides distasonis, Duncaniella_u_s* (u_s_ stands for “unnamed species”), *Duncaniealla muris* (Bacteroidetes), *Adlercreutzia caecimuris, Enterorhabdus_u_s, Adlercreutzia muris and Adlercreutzia mucosicola* (Actinobacteria) and *Ligilactobacillus animalis* and *Lactobacillales_u_s* (Firmicutes), with sex-specific enrichment between the taxa (4 in males, 5 in females, for details see Supplementary Figure 1; Supplementary Table 2). Characteristic only for Abx offspring were 8 taxa, including *Streptococcus thermophilus, Lactobacillus kisatatonis, Leuconostoc mesenteroides, Lactobacillus amylovorus, Liquorilactobacillus sucicola*, *Fructobacillus fructosus, Lactococcus lactis* and *Pediococcus acidilactici*, all belonging to Firmicutes. From them only *Streptococcus thermophilus* was enriched in male Abx group, the remaining 7 taxa were enriched in female Abx group (see LDA Supplementary Figure 2; Supplementary Table 2). Specific for only male controls were two taxa, *Bifidobacterium pseudolongum* (Actinobacteria) and *Limosilactobacillus reuteri* (Firmicutes), while absent only in female Abx cohort were three taxa: *Roseburia _u_s, Firmicutes _u_s* (Firmicutes) and *Muribaculum_u_s* (Bacterioidetes), all enriched in male controls. Only three taxa were present in all four cohorts: *Muribaculaceae_u_s, Ligilactobacillus murinus* and *Lactobacillus johnsonii* (Firmicutes), with significantly lower scores in the Abx groups compared to controls; and displaying sex-specific enrichment—(*Lactobacillus johnsonii* in male controls; *Muribaculaceae_u_s, Ligilactobacillus murinus* in female control cohorts). Thus, despite the similar levels of community richness and diversity between male and female offspring in control and in Abx groups, significant differences in microbiota composition (strain level) were observed.

### 3.3 Metagenomic predictive functional profiling

**Figure 6:**
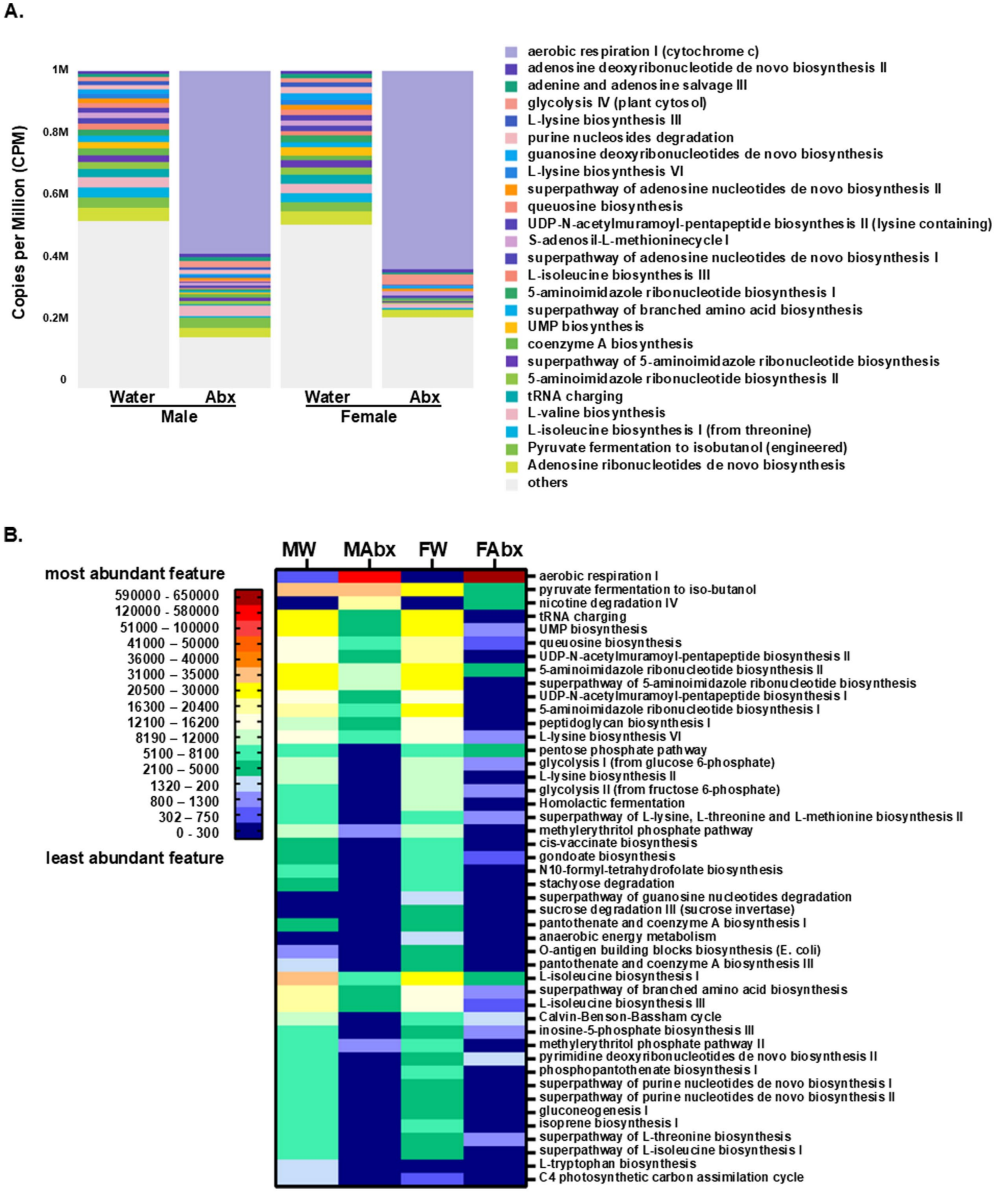
Predictive microbial functional profiling from WGS sequencing. Shown are the data from metabolic pathways enrichment analysis based on MetaCyc database (CosmosID). **(A)** Stacked bar aggregated by group (settings: most abundant feature/overall, top 25, ordered by default). To facilitate comparisons across multiple samples with different sequencing depths, the abundance values were subjected to Total-Sum Scaling (TSS) normalization to produce “Copies per million” (CPM). **(B)** Predictive metabolic pathways heat map—Abx treatment and sex-interaction. The heat map was created based on the top 50 dominant pathways, showing differential abundance of predictive metabolic pathways among the groups stratified by sex and treatment. A color gradient demonstrates the significant shifts in the bacterial metabolic activity in the offspring of Abx exposed dams and the sex-specific differences.

Metagenomic predictive functional profiling was also conducted to identify changes in the molecular, biochemical, and metabolic activities associated with the disturbed microbial communities in pups of Abx-exposed dams, using the CosmosID-HUB application and MetaCyc database [Caspi et al., 2016, web application (see text footnote 1)]. A total of 284 metabolic pathways were annotated. The generated visualization plots for metabolic pathways are shown in Figure 6 and Supplementary Figure 3. Consistent with the taxonomic findings, the Abx groups exhibited significant shifts in the predictive metabolic pathways including decreased overall alpha diversity (Supplementary Figure 3A, Shannon). No sex-dependent significant differences in alpha diversity were observed between control (male and female) and Abx (male and female) groups. The PCoA plots of Bray-Curtis distances (Supplementary Figure 3B) discriminated between controls and Abx cohorts, with large distances between the samples, indicating significant differences in the functional potential between the treatment and control groups. Again, no significant separation was observed between males and females in control or Abx groups. The shifts in the metabolic pathways in the offspring of Abx exposed dams is illustrated in Figure 6A (stacked bar, top 25 most abundant features) exhibiting significantly increased potential for aerobic respiration (more pronounced in females).

The functional enrichment analysis and visualization plots were generated using the CosmosID-HUB application as described in methods (Supplementary Figure 4 and see Supplementary Table 3 for the LDA scores and *p*-values). Of note, sex- and treatment-distinct enrichment pattern were observed: aerobic respiration I (cytochrome C), was significantly enriched in female Abx group, while biosynthesis of components of the peptidoglycan (which gives shape and rigidity to the bacterial cell walls) were enriched in female controls; as well as pantotenate, and coenzyme A biosynthesis III, while nicotine degradation IV pathway was detected in Abx groups, (enriched in male Abx); and L-tryptophane biosynthesis pathway was evident only in male control group.

The top 50 predictive metabolic pathways were used to create a heatmap (Figure 6B). Detected in all 4 groups were 9 metabolic pathways (aerobic respiration, pyruvate fermentation to isobutanol, UMP biosynthesis, queuosine biosynthesis, 5-aminoimidazole ribonucleotide biosynthesisII, L-lysine biosynthesis VI, L-isoleucine biosynthesis I, superpathway of branched amino acid biosynthesis and L-isoleucine biosynthesis III). Only the aerobic respiration pathway was increased in the Abx groups (from being significantly lower in female controls compared to male controls, shifted to significantly enriched in female Abx groups; see details in Supplementary Table 3). The pyruvate fermentation to isobutanol pathway was enriched in male controls, remained similar for male Abx, but was significantly decreased in female Abx group. The remaining 7 common for all pathways were significantly lower in Abx groups, compared to their corresponding controls. More specifically, UMP biosynthesis was enriched in female controls, significantly decreased in Abx groups (4^.^5-fold for male Abx and more than 31-fold in female Abx). Similar patterns were observed for the queuosine biosynthesis, 5-aminoimidazole ribonucleotide biosynthesis II and L-lysine biosynthesis II pathway. In contrast, L-isoleucine biosynthesis I, superpathway of branched amino acid biosynthesis, and L-isoleucine biosynthesis III were enriched in male controls and decreased significantly in Abx groups (for male Abx resp. 6-folds, 4^.^5-fold and 4^.^2-fold; and for female Abx—6^.^4-fold, 17-fold and 20-fold resp.). Another set of predicted metabolic pathways (total of 16) was present only in control offspring (both male and female). From them enriched in male offspring were 7 pathways (phosphopantothenate biosynthesis I, superpathway of purine nucleotide *de novo* biosynthesis I and II, gluconeogenesis I, isoprene biosynthesis I, superpathway of L-isoleucine biosynthesis I and C4 photosynthetic carbon assimilation cycle, NADP-ME type), and 9 in female offspring (L-lysine biosynthesis II, homolactic fermentation, cis-vaccenate biosynthesis, N10-formyl-tetrahydrofolate biosynthesis, stachyose degradation, O-antigen building blocks biosynthesis (*E. coli*), pantothenate and coenzyme A biosynthesis I and III). We observed 1 pathway exhibited in male control offspring only (L-tryptophan biosynthesis) and in female control offspring only 3 pathways (superpathway of guanosine nucleotides degradation, sucrose degradation III (sucrose invertase) and anaerobic energy metabolism). Present in Abx groups only was nicotine degradation IV pathway, while present in 3 groups but not in female Abx were 8 pathways (e.g., tRNA charging, UDP-N-acetylmuramoyl-pentapeptide biosynthesis II and I, superpathway of 5-aminoimidazole ribonucleotide biosynthesis, 5-aminoimidazole ribonucleotide biosynthesis, peptidoglycan biosynthesis I, methylerythritol phosphate pathway I and II). Of note, another set of pathways were observed in 3 of the groups, but were not displayed in male Abx offspring, for example pentose phosphate pathway, glycolysis I, superpathway of L-lysine, L-threonine and L-methionine biosynthesis II, gondoate biosynthesis (anaerobic), Calvin-Benson-Bassham cycle, inosine-5-phosphate biosynthesis III, pyrimidine deoxyribonucleotides de novo biosynthesis II and superpathway of L-threonine biosynthesis. Thus, our results indicate strain differences in the microbial composition between females and males in control groups and common and sex-specific effects of the Abx-induced microbiome shifts, and in their respective predictive functionality which collectively, offers the prospect of functionally relevant cellular influences on the biochemical communication between these microbial symbionts and their hosts.

### 3.4 Perturbed early life colonization alters the responses to acute metabolic stress only in male pups

**Figure 7:**
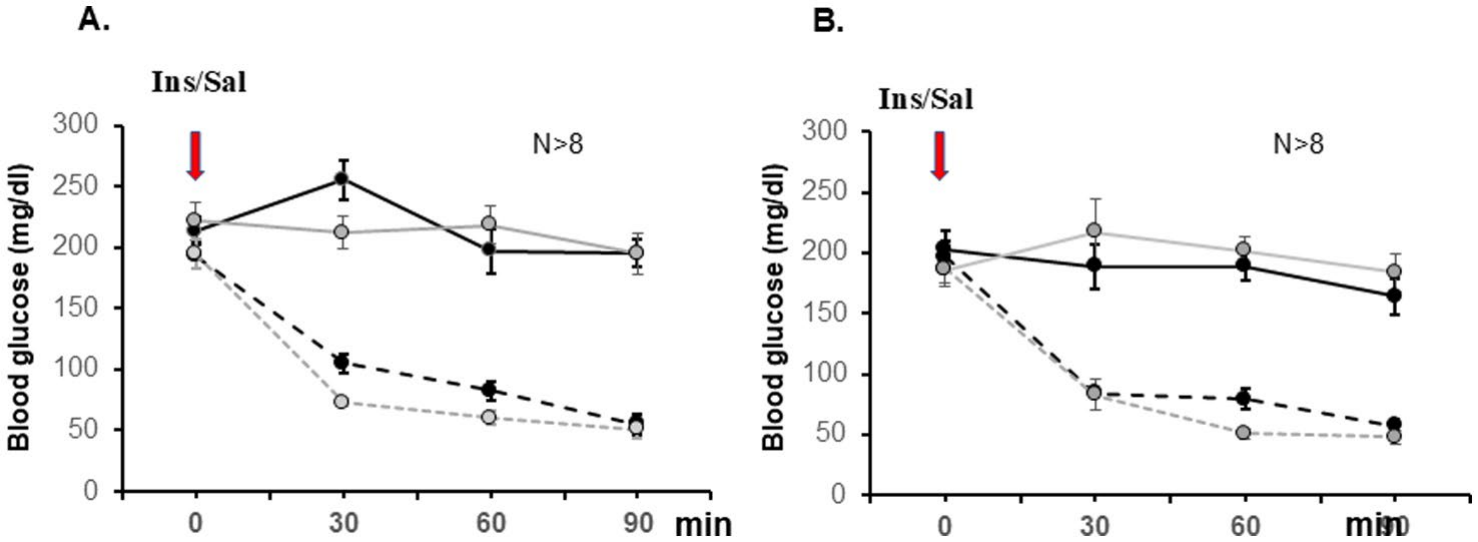
Basal glucose levels and magnitude of insulin-induced hypoglycemia were similar between control (black lines) and Abx-treated (gray lines) mice. Glucose values (mg/dL) for saline (solid lines) and insulin-treated (broken lines) groups are shown as mean ±SEM, *N* ≥ 8. Data are summarized from 12 independent experiments and analyzed by Two Way Repeated Measures ANOVA, followed by all pairwise multiple comparison procedures (Bonferroni t-test): Control Sal vs. Control Ins differed at 30, 60, and 90 min in males **(A)**, *p* < 0^.^001, t = 7^.^913; *p* < 0^.^001, t = 9^.^545; *p* < 0^.^001, t = 9^.^589; and in females **(B)**; and Abx Sal differed from Abx Ins for the same time points (*p* < 0^.^002, t = 3^.^929; *p* < 0^.^001, t = 5^.^760; and *p* < 0^.^001, t = 6^.^382, resp.).

**Figure 8:**
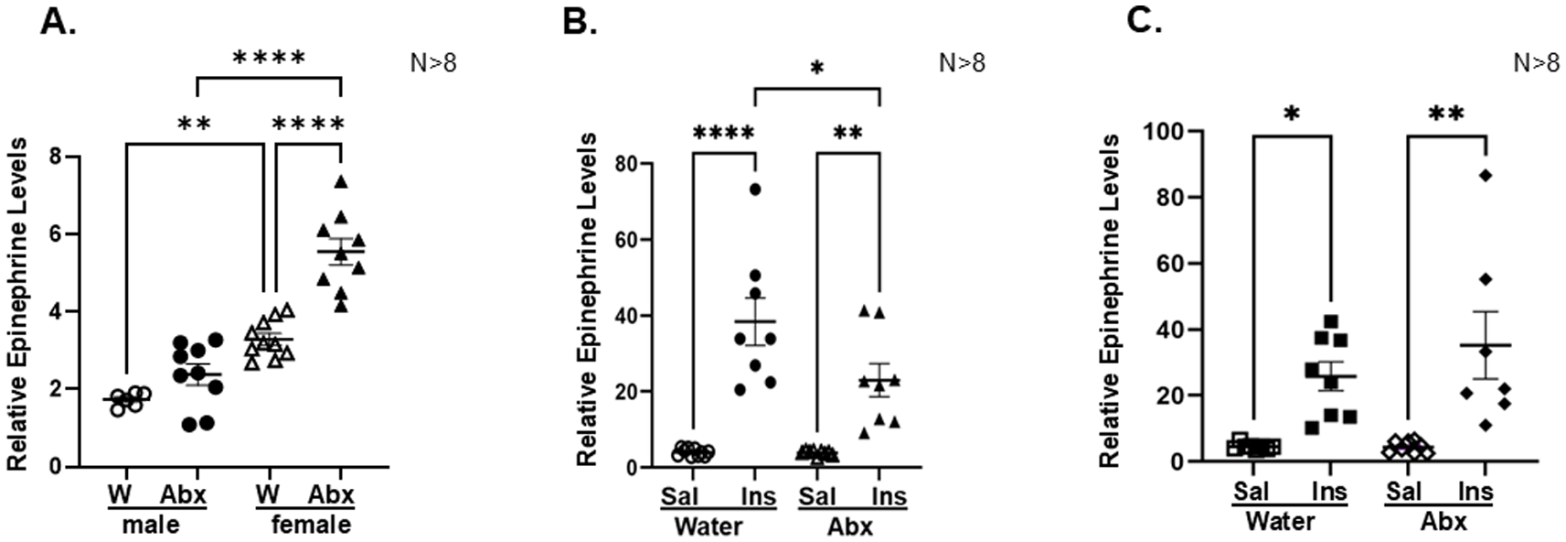
Disturbed neonatal colonization reduces epinephrine responses to hypoglycemia only in male offspring: Epinephrine levels were analyzed in urinary samples collected before (0′ time point, **A**) and 90 min after injecting insulin/saline (90 min, **B**—males; **C**—females). For each experimental group catecholamine levels were normalized to creatinine content in the same sample. Results are presented as mean ± SEM, *N* ≥ 8. Two Way Analysis of Variance and Tukey’s multiple comparisons test was used, ^*^p < 0^.^05; ^**^p < 0^.^003; ^****^p < 0^.^0001. Each dot represents the value of an individual animal. **(A)** 0 min—significant interaction between sex and treatment (Water/Abx): *F*(1,30) = 10^.^10 *p* = 0^.^034; row factor—sex *F*(1,30) = 32^.^52 *p* < 0^.^0001; column factor—treatment (Water/Abx) *F*(1,30) = 86.42 *p* < 0.0001; **(B)** Male 90 min—Significant interaction between groups (Water/Abx) and (Ins/Sal) treatment *F*(1,31) = 4^.^819 *p* = 0^.^0358; row factor—groups *F*(1,31) = 5^.^038 *p* = 0^.^0321; column factor—treatment *F*(1,31) = 59^.^21 *p* < 0^.^0001; **(C)** Female 90 min: no interaction, *F*(1,27) = 0^.^8763, *p* = 0^.^3575; row factor—groups (Water/Abx) *F*(1,27) = 26^.^03 *p* < 0^.^0001; column factor—treatment (Ins/Sal) *F*(1,27) = 0^.^8225 *p* = 0^.^3725.

Next, we aimed to determine whether the observed differences in microbial composition and functionality will be associated with altered ability of the host to respond to stress. We again used our innovative research approach to study gut-microbiota-brain interface at individual synapse (splanchnic nerve fibers innervating the adrenal medulla, a simple model of catecholaminergic neurotransmission) during exposure to metabolic stress (insulin-induced hypoglycemia detected in the hypothalamus) (Giri et al., 2019; LaGamma et al., 2021). The experiment is outlined in Figure 1A. At PND 27 all pups were weaned, and the next day fasted for 3 h (Figure 1A, Methods), before being subdivided into saline-treated (Sal) and insulin-treated (Ins) subgroups (*n* > 10). We had total of 8 experimental groups: males and females, offspring of controls and Abx exposed, Sal- or Ins-treated. Basal glucose levels and the magnitude of insulin-induced hypoglycemia were not significantly different between the groups (Figure 7A, males and Figure 7B, females), suggesting that all groups exposed to insulin experienced similar extent of metabolic stress. Baseline urinary epinephrine levels were measured before exposure to handling stress/injections. The results are summarized in Figure 8A. Male offspring of controls (dams drinking regular water, W) had significantly lower relative epinephrine levels as compared to female offspring of controls (^**^p = 0^.^0014), indicating sex-specific differences as reported by others (Colomer et al., 2012; Weinstock et al., 1998). Exposure of the dams to Abx and the corresponding perturbed colonization of the gut in the offspring was associated with significantly higher basal relative epinephrine levels in females, as compared to control female/male or Abx male weanlings (^****^p < 0^.^0001). Taken together, these data support the notion that a microbiome derived signal is reaching the nervous system and can alter the efficacy of a classic reflex arc’s function in a meaningful way at the whole animal level.

The effects of antibiotic-induced microbiota depletion on plasma glucagon and corticosterone, and urinary epinephrine responses to acute metabolic stress were also assessed in male and female pups of control and Abx offspring. We did not observe significant changes in the corticosterone and glucagon levels between the groups at the time of sacrifice (90 min after injecting insulin or saline, data not shown). The relative epinephrine levels in saline injected animals were also not significantly different between the groups (males vs. females, water control vs. Abx exposed) at 90 min. The effect of insulin treatment was analyzed for male (Figure 8B) and female (Figure 8C) offspring separately: as expected, exposure to hypoglycemia significantly increased relative epinephrine levels in males and females in both, controls (offspring of regular water drinking mothers) and Abx groups (offspring of mothers drinking Abx cocktail). However, compared to their respective saline treated controls, only male Abx pups displayed significantly reduced epinephrine response (Figure 8B), not evident in the female Abx pups (Figure 8C).

### 3.5 Adrenal medullary transcriptome profiling: effects of sex and microbiome depletion

To elucidate how maternal antibiotics-driven early-life microbiota perturbation affects acute responses to hypoglycemia in the offspring, we performed polyA RNA sequencing as described in Methods. The reads were mapped to approximately 50,000 genes and the differentially expressed genes between the groups were identified using DESeq2 (Love et al., 2014). First, we compared the AM transcriptome in control and Abx offspring. At significance cutoff of q < 0^.^05 (log2 fold change FC = 1) only 11 transcripts were detected in the comparison of male control (offspring of regular water drinking dams) vs. male Abx (pups of Abx exposed dams), see Supplementary Table 4. From them, 4 transcripts (among them Cntn5—contactin 5, a cell adhesion molecule, mediating cell surface interactions during nervous system development) were identified as DEG in male control adrenal medullae, while 7 transcripts were only detected as DEGs in male Abx (including Igfbp1—Insulin-like Growth Factor Binding Protein 1, Rnase1 and DKK—dickkopf WNT signaling pathway inhibitor 1). Of note, approximately 200 protein coding transcripts with a *p* value < 0^.^05 were identified (data available on request), indicating an effect of gut dysbiosis on AM transcriptional activity in the host in a gene specific manner: 53 genes displayed increased expression in the Abx group, 118 were significantly down regulated, no expression was detected in Abx for 12, and 14 new transcripts (evident only in the Abx group) were observed. In female offspring the comparison female control vs. female Abx revealed 45 genes differentially expressed at significance cutoff of q < 0.05 (log2 fold change FC = 1), as shown in Supplementary Table 5. While only 2 transcripts were not identified in female control as differentially expressed (Barx2—a homeobox transcription factor and Cntn5), 26 transcripts were not present in the female Abx DEG list. Induced after Abx exposure were 11 transcripts [including Syt2—synaptotagmin 2, synaptic vesicle membrane protein which functions as calcium sensor for neurotransmitter release linked to presynaptic congenital myasthenic syndromes and motor neuropathies (Bauche et al., 2020)]; Cntnap2—contactin associated protein 2, implicated in multiple neurodevelopmental disorders like Gilles de la Tourette syndrome, schizophrenia, epilepsy, autism, ADHD and intellectual disability (Rodenas-Cuadrado et al., 2014); Kcnmb4—potassium calcium-activated channel subfamily M regulatory beta subunit 4, involved in neuronal excitability and the enhanced synaptic transmission in neuropathic pain state, may result in anxiety-like behavior (Zhao et al., 2020). (These data were not subjected to predictive functional pathway analyses due to the limited number of DEGs.)

**Figure 9:**
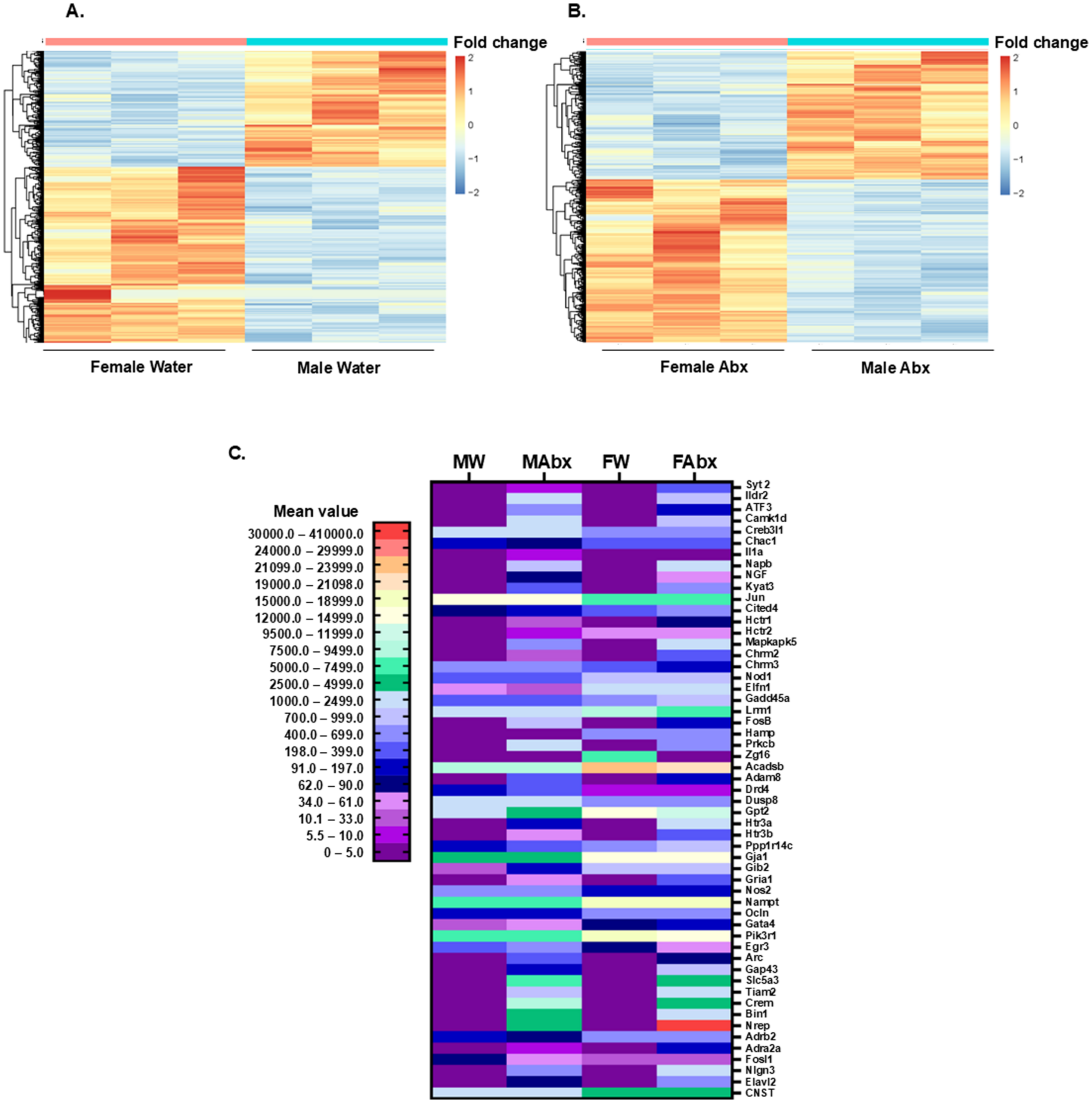
PolyA mRNA sequencing and differential gene expression analysis in adrenal medullae. **(A)** Heat map of DEGs: female water vs. male water control groups: padj < 0^.^05, FC (fold change) cutoff = 1, *n* = 1,471. Each column represents a biological replicate (*n* = 3/group). **(B)** Heat map of DEGs: female Abx vs. male Abx groups, padj < 0^.^05, FC cutoff = 1, *n* = 1,733. The color intensity of the symbols represents the fold change of upregulation (red) or downregulation (blue) of each gene. **(C)** Heat map created based on manually selected DEGs in all 4 groups relevant to synaptic transmission, exocytosis/stress responses, neuroinflammation and response to insulin. A color gradient (based on mean values) demonstrates the significant shifts in DEGs in the offspring of Abx exposed dams and the sex-specific differences.

Next, we included “sex” as variable in the comparison analysis: in controls “Female versus Male” 1,471 DEGs were detected at significance cutoff of q < 0.05 (log2 fold change FC = 1), visualized on the heat map in Figure 9A. To get more manageable counts, the data was filtered based on log2 fold change (FC = 2) reducing the gene numbers to 568. Of note, significantly more transcripts (397) were enriched in females (displaying positive FC value) compared to the 171 DEGs with negative FC value (higher expression level in males, see Supplementary Table 6). Maternal antibiotics in early life shifted the transcriptional activity in the adrenal medullae of the offspring in a sex-dependent manner: the comparison of “Female Abx versus Male Abx” groups at Log2 FC = 1 revealed higher number of DEG genes (1,733) (Figure 9B), and altered the ratio of positive and negative FC values in both groups (female Abx and male Abx). After FC = 2 filtering, the DEG numbers were reduced to 640, with 339 transcripts enriched in “Female Abx” and 301—in “Male Abx” (Supplementary Table 7).

To better illustrate the effect of sex and early life microbiome perturbation on adrenal transcriptome landscapes we manually selected several genes, relevant to synaptic transmission, exocytosis/stress responses, neuroinflammation and response to insulin, and visualized the data on a heat map (Figure 9C). From the selected 55 DEGs, 26 were present in all 4 groups and 27 were evident only in Abx groups (Male and Female); one transcript [interleukin 1 alpha, Il1a, pro-inflammatory cytokine known to stimulate genes involved in inflammation and immunity, included in the reported cytokine imbalances in ASD (Goines and Ashwood, 2013)] met the requirements for DEG only in Male Abx group; and zymogen granule protein 16 gene (Zg16) was expressed only in females (significantly higher in Female control group compared to Female Abx). Enriched in female control group (compared to male control group) were several transcripts in our selection (Smith et al., 2014), including Chac1 [Glutathione-specific gamma-glutamylcyclotransferase 1, pro-apoptotic molecule involved in the regulation of neuronal differentiation and oxidative balance (Sun et al., 2024)], Cited4 (CBP/p300 transcriptional coactivator), Hctr2 (hypocretin receptor 2, involved in regulation of feeding behavior), Nod1 [nucleotide binding oligomerization domain containing 1, the encoded protein binds bacterial peptidoglycans and initiates inflammation, and modulates epinephrine secretion in response to stress (Xiang et al., 2021)], Elfn1 [extracellular leucine rich repeat and fibronectin type III domain containing 1, involved in synapse organization (Ayhan and Dursun, 2024)], Gadd45 (growth arrest and DNA damage inducible alpha), Lrrn1 (leucine rich repeat neuronal 1, predicted positive regulator of synapse assembly), Hamp (hepcidin antimicrobial peptide), Acadsb (acyl-CoA dehydrogenase short/branched chain), Gpt 2 [glutamic—pyruvic transaminase 2, mutations in this gene are associated with intellectual and developmental disability (Ouyang et al., 2016)], Ppp1r14c (protein phosphatase 1 regulatory inhibitor subunit 14C), Gja1 (gap junction protein alpha 1), Gjb2 (gap junction protein beta 2), Nampt [nicotinamide phosphoribosyltransferase—essential pleiotropic protein involved in basic cellular functions (Zhang et al., 2017)], Ocln (occludin—key protein in formation of tight junctions), Gata4 (zinc finger transcription factor), Pik3r1 (phosphoinositide-3-kinase regulatory subunit 1), BDNF (brain-derived neurotrophic factor) and CNTS [consortin, integral membrane protein binding partner of connexins (del Castillo et al., 2010)]. In male control group there were 7 transcripts with higher expression levels compared to female control: cAMP responsive element binding protein 3 like 1—Creb3l1, Jun proto-oncogene, AP-1 transcription factor subunit—Jun, cholinergic receptor muscarinic 3—Chmr3, dopamine receptor D4—Drd4, dual specificity phosphatase 8—Dusp8, nitric oxide synthase 2—inducible Nos2, and early growth response 3 gene—Egr3. Maternal antimicrobials had distinct effects on adrenal medullary transcriptome profiles in the offspring: increased the expression in both, male and female offspring [i.e., Gadd45, Cited4, Ppp1r14c, Gata4, Nlgn3 – neuroligin 3, postsynaptic adhesion molecule, among the first genes linked to ASD and intellectual disability (Nguyen et al., 2020)], decreased the transcription in both compared to corresponding water controls (Chac1, Jun, Nod1, Elfn1, Acadsb, Gja1, Nampt, CNST), or had divergent (opposite) transcriptional effects (Hctr2, Chrm3, Lrrn1, Drd4, Dusp8, Gpt2, Gjb2, Nos2, Ocln, Pik3r1, Egr3, Adrb2–beta 2 adrenergic receptor), for details see Table 1. In our selection we also included number of differentially expressed transcription factors: present in all groups, i.e., Creb3l1, Egr3 and Fosl1—enriched in male controls; and affected by maternal antimicrobials treatment in opposite direction—further increased in male, but down regulated in female offspring; Jun—enriched in males, down regulated by microbiome depletion in both sexes; or present only in Abx groups—ATF3, FosB and Crem, enriched in male pups. Evidently, several sex-specific DEGs were detected in our study following Abx-induced gut dysbiosis that are important in transcriptional regulation, affect synaptic function and/or inflammation and have been associated with ASD (neurodevelopmental disorder prevalent in males), which further emphasize the importance of environmental factors (gut microbiome) in its etiology and as potential therapeutic targets (Taniya et al., 2022; Nguyen et al., 2020).

**Table 1:** Comparison of selected DEGs: effect of early life microbiome perturbation and sex.

		Mean values			
Gene ID	Name	Male water	Male Abx	Female water	Female Abx
ENSMUSG00000026452	Syt 2		9		283
ENSMUSG00000040612	Ildr2		1,862		711
ENSMUSG00000026628	ATF3		576		122
ENSMUSG00000039145	Camk1d		2,106		992
ENSMUSG00000027230	Creb3l1	1,478	1,733	662	610
ENSMUSG00000027313	Chac1	101	89	343	312
ENSMUSG00000027399	Il1a		9		0
ENSMUSG00000027438	Napb		746		1,684
ENSMUSG00000027859	NGF		84		37
ENSMUSG00000040213	Kyat3		295		626
ENSMUSG00000052684	Jun	13,536	13,396	6,225	5,666
ENSMUSG00000070803	Cited4	85	101	381	453
ENSMUSG00000028778	Hcrtr1		24		79
ENSMUSG00000032360	Hcrtr2	3	5.5	40	34
ENSMUSG00000029454	Mapkapk5		527		1,306
ENSMUSG00000045613	Chrm2		16		228
ENSMUSG00000046159	Chrm3	419	441	207	197
ENSMUSG00000038058	Nod1	332	309	763	719
ENSMUSG00000048988	Elfn1	47	28	1,322	1,283
ENSMUSG00000036390	Gadd45a	266	293	645	805
ENSMUSG00000034648	Lrrn1	1,601	2,484	8,828	6,738
ENSMUSG00000003545	FosB		736		128
ENSMUSG00000050440	Hamp	1.3	0.7	597	478
ENSMUSG00000052889	Prkcb		1,149		530
ENSMUSG00000049350	Zg16	0	0	7,464	1
ENSMUSG00000030861	Acadsb	8,838	8,340	22,763	21,004
ENSMUSG00000025473	Adam8		273		98
ENSMUSG00000025496	Drd4	163	226	8	9
ENSMUSG00000037887	Dusp8	1,401	1,529	611	584
ENSMUSG00000031700	Gpt2	2,189	2,901	12,167	11,566
ENSMUSG00000032269	Htr3a		195		1,042
ENSMUSG00000008590	Htr3b		38		289
ENSMUSG00000040653	Ppp1r14c	172	205	614	704
ENSMUSG00000050953	Gja1	3,530	3,277	14,771	13,132
ENSMUSG00000046352	Gjb2	33	119	998	939
ENSMUSG00000020524	Gria1		46		345
ENSMUSG00000020826	Nos2	417	514	156	158
ENSMUSG00000020572	Nampt	6,431	5,807	18,535	17,752
ENSMUSG00000021638	Ocln	167	171	507	441
ENSMUSG00000021944	Gata4	33	38	83	92
ENSMUSG00000041417	Pik3r1	5,866	6,239	15,323	13,969
ENSMUSG00000033730	Egr3	253	504	76	54
ENSMUSG00000022602	Arc		383		78
ENSMUSG00000047261	Gap43		101		754
ENSMUSG00000089774	Slc5a3		7,292		3,585
ENSMUSG00000023800	Tiam2		804		1,916
ENSMUSG00000063889	Crem		9,385		4,629
ENSMUSG00000024381	Bin1		4,341		2,131
ENSMUSG00000042834	Nrep		3,153		40,632
ENSMUSG00000045730	Adrb2	94	70	426	566
ENSMUSG00000033717	Adra2a		9		122
ENSMUSG00000024912	Fosl1	62	49	11	12
ENSMUSG00000031302	Nlgn3		651		1,471
ENSMUSG00000008489	Elavl2		64		555
ENSMUSG00000038949	CNST	1,395	1,115	2,894	2,647

Overview of the effects of maternal Abx exposure on DEG in adrenal medullae of male and female offspring compared to their respective controls (offspring of water drinking dams). The data represents “mean values” (n = 3) of manually selected DEGs in all 4 groups relevant to synaptic transmission, exocytosis/stress responses, neuroinflammation and response to insulin.

In summary, our RNA seq data revealed: (1) Early life gut microbiome dysbiosis affected transcriptional landscape in the adrenal medulla of the host; (2) female and male control weanlings (dams drinking regular water during nursing) displayed different transcriptional profiles; (3) in the offspring of Abx exposed dams the transcriptional activity in the adrenal medullae was affected in a sex- and gene-specific manner compared to their respective controls.

**Figure 10:**
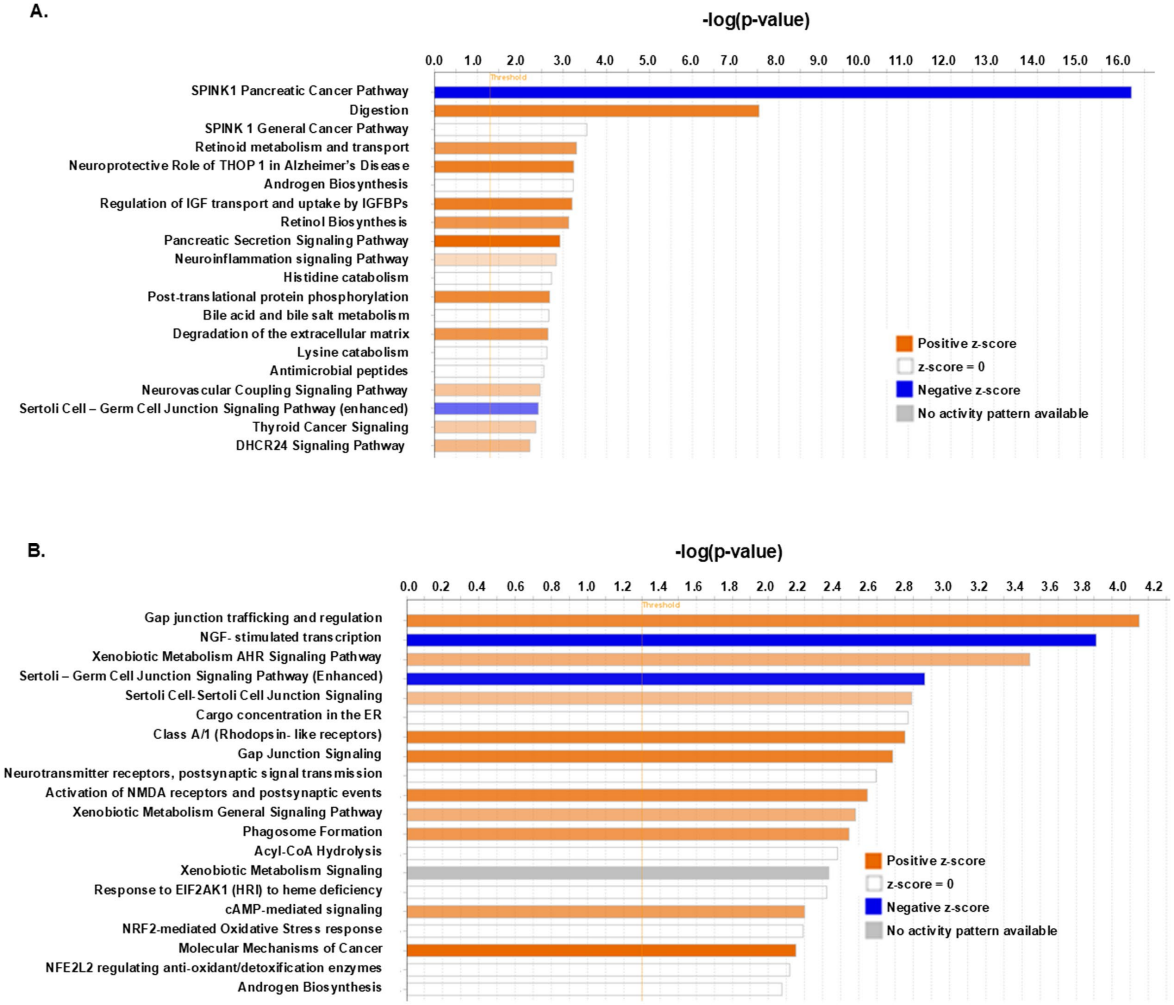
Predictive Canonical Pathways enriched in the AM of control and Abx offspring. **(A)** Selection of predictive top 20 IPA Canonical Pathways that are significantly enriched for DEGs between female and male controls, FC = 1; with z-score > 2^.^0 and padj < 0^.^05. Bar-chart color indicates predicted direction: blue—negative z-score; red—positive z-score; white—z-score = 0; and the broken line (-----) indicates the threshold of 1^.^3. **(B)** Selection of predictive top 20 IPA Canonical Pathways that are significantly enriched for DEGs in the offspring of Abx treated dams (females vs. males).

To evaluate the potential biological impact of the observed DEGs between cohorts in the RNA seq data we performed IPA core predictive functional enrichment analysis. The most significant differences in the Canonical Pathways category (top 20) between female and male control groups are shown in Figure 10A. The top differentially regulated pathways included SPINK 1—(serine protease inhibitor, Kazal type 1) pathway, Digestion, and Retinoid metabolism and transport, Neuroinflammation signaling pathway (enriched in females) and others, for details see Table 2.

**Table 2:** IPA predictive functional enrichment analysis: female water vs. male water.

Ingenuity Canonical Pathways	−log *p*-value	Ratio	z-score	Molecules
SPINK1 pancreatic cancer pathway	16.2	0.233	−3.21	CELA1, CELA2A, CELA3B, CLPS, CPA1, CPA2, CPB1, CTRB2, CTRL, KLK3, PRSS2, PRSS3, Prss3b, SPINK1
Digestion	7.55	0.261	2.449	Amy2a1, CLPS, MGAM, PNLIP, PNLIPRP1, PNLIPRP2
SPINK1 general cancer pathway	3.55	0.0725	0	PIK3C2G, PRSS2, PRSS3, Prss3b, SPINK1
Retinoid metabolism and transport	3.3	0.0909	2	AKR1C3, CLPS, GPC5, PNLIP
Neuroprotective role of THOP1 in AD	3.24	0.0492	2.449	CTRB2, CTRL, KLK3, PRSS2, PRSS3, ST14
Androgen biosynthesis	3.23	0.15		AKR1C3, GSTA5, SRD5A2
Regulation of IGF transport and uptake by IGFBPs	3.2	0.0484	2.449	C3, F5, IGFBP1, KLK3, SERPINA10, SPP1
Retinol biosynthesis	3.13	0.0816	2	AKR1C3, PNLIP, PNLIPRP1, PNLIPRP2
Pancreatic secretion signaling	2.92	0.0323	2.828	Amy2a1, Amy2b, CA8, CELA3B, LIPN, PIK3C2G, PNLIP, PNLIPRP1
Neuroinflammation signaling	2.84	0.0284	0.707	BDNF, GABRB1, HMOX1, KLK3, Naip1 (includes others), NOX4, PIK3C2G, PLA2G1B, PLA2G4D
Histidine catabolism	2.72	0.25		AMDHD1, HDC
Post-translational protein phosphorylation	2.68	0.0467	2.236	C3, F5, IGFBP1, SERPINA10, SPP1
Bile acid and bile salt metabolism	2.66	0.0968		AKR1C3, AKR1D1, CH25H
Degradation of the extracellular matrix	2.64	0.0606	2	A2M, CDH1, SPP1, TMPRSS6
Lysine catabolism	2.62	0.222		AASS, PIPOX
Antimicrobial peptides	2.55	0.0882		PRSS2, PRSS3, REG3A
Neurovascular coupling signaling	2.46	0.0302	1.134	CTH, GABRB1, GJA1, KCNJ4, KCNMB4, PLA2G1B, PLA2G4D
Sertoli cell-germ cell junction signaling pathway (enhanced)	2.42	0.0297	−1.89	A2M, CDH1, CLDN23, CXADR, FOSL1, GJA1, PIK3C2G
Thyroid cancer signaling	2.36	0.0506	1	BDNF, CDH1, KLK3, PIK3C2G
DHCR24 signaling pathway	2.22	0.0365	1.342	APOF, C3, HMOX1, PIK3C2G, SAA1

Predictive top 20 IPA Canonical Pathways that are significantly enriched for DEGs between female (positive z-score) and male controls (negative z-score), FC = 1; with z-score > 2^.^0 and padj < 0^.^05.

The Canonical Pathways that underwent the most significant changes in the Abx groups are shown in Figure 10B and Table 3. Among them were Gap junction trafficking and regulation (enriched in females), NGF stimulated transcription (enriched in males, important for the growth and survival of diverse peripheral and central neurons), Gap junction signaling and Xenobiotic metabolism and aryl hydrocarbon receptor signaling pathway (enriched in females). Given that adrenal medulla gap junctional communication is another component of the stimulus-secretion coupling regulation (Colomer et al., 2012; Guerineau, 2018; Eiden and Jiang, 2018; Berends et al., 2019), and the reported wider expression of gap junctions in females (Martin et al., 2001) vs. males (Colomer et al., 2008), the predicted here canonical pathways may contribute to the observed differences in basal and stress-stimulated epinephrine release.

**Table 3:** IPA predictive functional enrichment: female Abx vs. male Abx.

Ingenuity Canonical Pathways	−log (*p* value)	Ratio	Z-score	Molecules
Gap junction trafficking and regulation	4.05	0.118	2	GJA1, GJB2, TUBB2B, TUBB3
NGF-stimulated transcription	3.81	0.103	−2	ARC, EGR3, FOSB, FOSL1
Xenobiotic metabolism AHR signaling	3.44	0.0575	1.342	ALDH3A1, GSTA3, GSTA5, IL1A, UGT1A1
Sertoli cell-germ cell junction signaling (enhanced)	2.86	0.0297	−1.89	A2M, CXADR, FOSB, FOSL1, GJA1, IL1A, PIK3C2G
Sertoli cell-sertoli cell junction signaling	2.79	0.0288	1.134	A2M, CDH22, GJA1, IL1A, PIK3C2G, TUBB2B, TUBB3
Cargo concentration in the ER	2.77	0.0882		F5, FOLR1, GRIA1
Class A/1 (rhodopsin-like receptors)	2.75	0.0252	2.121	ADRA2A, ADRB2, C3, CHRM2, DRD4, HCRTR2, OXTR, RXFP1
Gap junction signaling	2.69	0.0246	2.121	GJA1, GJB2, GRIA1, OXTR, PIK3C2G, PLA2G4D, TUBB2B, TUBB3
Neurotransmitter receptors and postsynaptic signal transmission	2.59	0.182		HTR3A, HTR3B
Activation of NMDA receptors and postsynaptic events	2.55	0.0476	2	ERBB4, GRIA1, TUBB2B, TUBB3
Xenobiotic metabolism general signaling	2.48	0.035	1.342	GSTA3, GSTA5, HMOX1, PIK3C2G, UGT1A1
Phagosome formation	2.45	0.0172	1.732	ADRA2A, ADRB2, C3, CHRM2, DRD4, HCRTR2, HMOX1, MYO1A, OXTR, PIK3C2G, PLA2G4D, RXFP1
Acyl-CoA hydrolysis	2.38	0.143		ACOT1, ACOT4
Xenobiotic metabolism signaling	2.33	0.0238		ALDH3A1, GSTA3, GSTA5, HMOX1, IL1A, PIK3C2G, UGT1A1
Response of EIF2AK1 (HRI) to heme deficiency	2.32	0.133		ATF3, TRIB3
cAMP-mediated signaling	2.2	0.0254	1,633	ADRA2A, ADRB2, CHRM2, DRD4, PDE6A, SMPDL3B
NRF2-mediated oxidative stress response	2.19	0.0253		CYP2F1, FOSL1, GSTA3, GSTA5, HMOX1, PIK3C2G
Molecular mechanisms of cancer	2.15	0.0152	2.496	ADRA2A, ADRB2, BMP3, CHRM2, CSF2RB, DRD4, HCRTR2, IL1A, MMP27, Naip1 (includes others), OXTR, PIK3C2G, RXFP1
NFE2L2 regulating antioxidant detoxification enzymes	2.12	0.105		GSTA3, HMOX1
Androgen biosynthesis	2.07	0.1		GSTA5, SRD5A2

Predictive top 20 IPA Canonical Pathways that are significantly enriched for DEGs in the offspring of Abx treated dams (female vs. male comparison).

### 3.6 Effects of maternal Abx on anxiety-like behavior and locomotor activity measured by OF test

**Figure 11:**
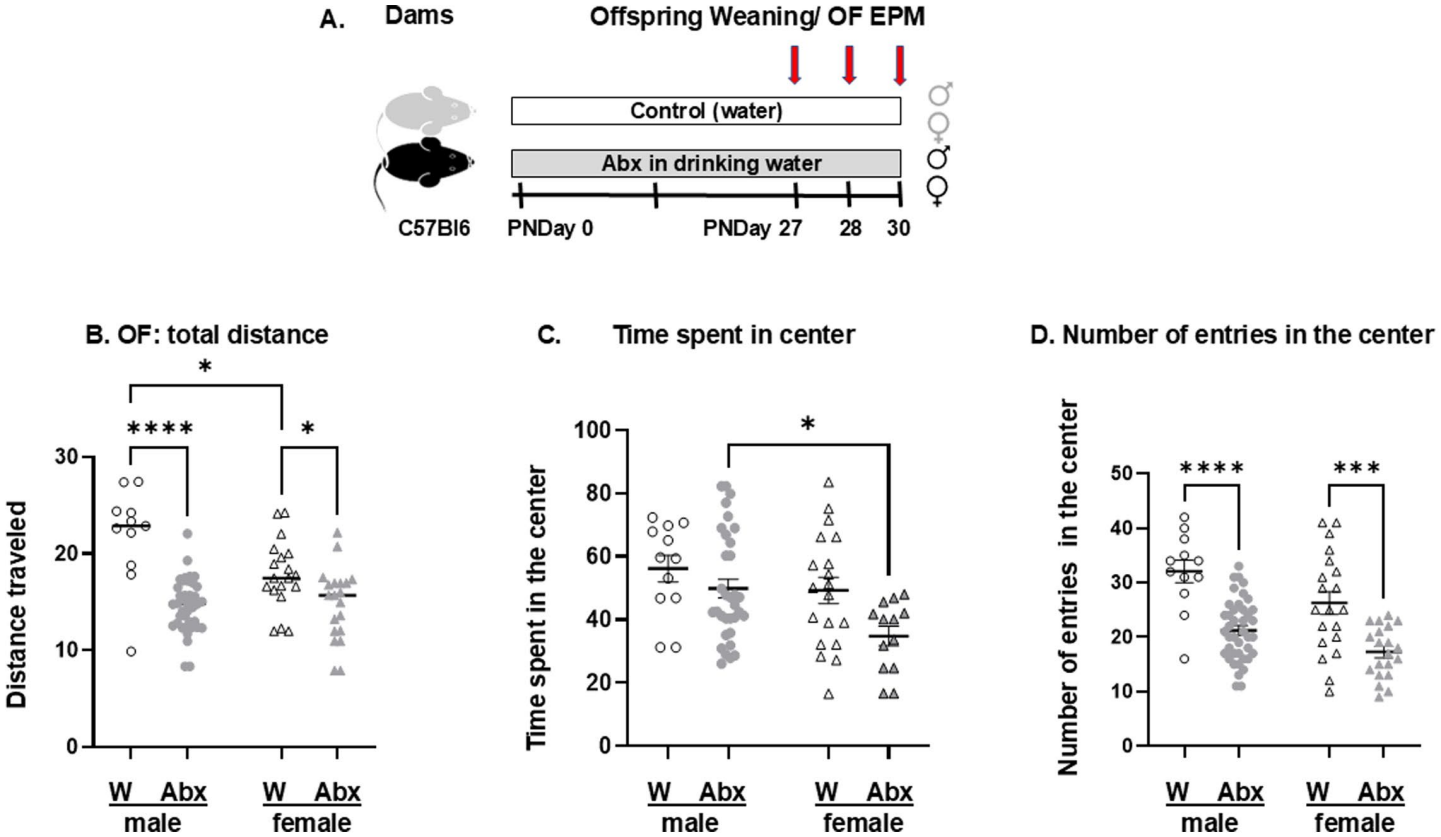
**(A)** Experimental design for behavioral tests: Dams were divided into 2 major groups: control (drinking regular sterile water) and Abx (mice given mixture of broad-spectrum antibiotics in the drinking water) starting from birth (PND 0). On their corresponding PND 27 each offspring was weaned and transferred to new cages (2–5 per cage, sex- and treatment matched) with the continuous respective treatments. On the next day (PND 28) the pups were subjected to OF test (first male pups, followed by the females as described in Methods). The EPM test was performed on PND 30. Open Field test: On PND 28 all pups were tested for: **(B)** Total distance traveled; **(C)** Time spent in the center of the OF arena; and **(D)** Number of entries into the center of the arena. The data passed the normality test and were analyzed using two way-ANOVA, followed by Tukey’s multiple comparisons test. Each dot represents a value for an individual animal. Circles—males, triangles—females. All data are expressed as means ± SEM, *n* ≥ 12. ^*^p < 0^.^05, ^***^p < 0^.^0002, ^****^p < 0^.^0001.

Next, we evaluated potential effects of maternal Abx during nursing on brain/behavior of the weanlings. The experimental design is shown in Figure 11A. The OF test provides an initial screen for anxiety-related and exploratory behaviors. Offspring of Abx dams displayed decreased locomotor activity: they traveled significantly less total distance compared to their respective controls, and female controls explored less than male controls (Figure 11B), with interaction between sex and treatment groups: *F*(1,86) = 7^.^002 *p* = 0^.^0097; *F*(1,86) = 40^.^47 *p* < 0^.^0001 (row—treatment); *F*(1,86) = 5^.^84 *p* = 0^.^018 (column—sex). Abx females spent significantly less time in the center of the arena compared to Abx males: *F*(1,73) = 6^.^800 *p* = 0^.^011 (row—treatment); *F*(1,73) = 7^.^533 *p* = 0^.^0076 (column—sex, Figure 11C), indicating increased anxiety-like measures. The Abx offspring had significantly fewer number of entries into the center of the arena compared to their respective controls (Figure 11D). The data passed the normality test and were analyzed using two way-ANOVA, followed by Tukey’s multiple comparisons test *F*(1,89) = 43^.^89 *p* < 0^.^0001 (row—treatment); *F*(1,89) = 10^.^77 *p* < 0^.^0015 (column—sex). No significant sex differences were found between male and female control and Abx groups in the number of entries into the center.

### 3.7 The offspring of Abx dams display different behavioral outcomes on the EPM test

**Figure 12:**
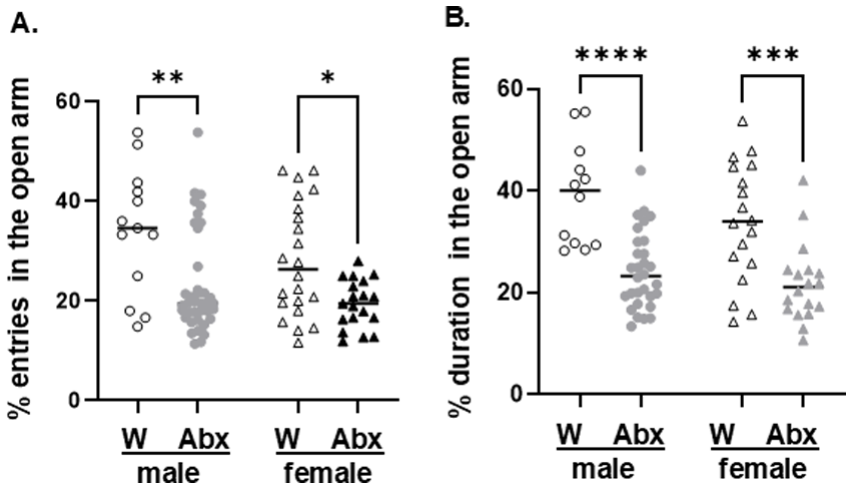
Elevated Plus Maze test: on their PND 30 pups were tested for: **(A)** % entries in the Open Arm (OA) of the EPM, and **(B)** % duration in the OA. The data passed the normality test and were analyzed using two way-ANOVA, followed by Tukey’s multiple comparisons test. Each dot represents a value for an individual animal. Circles—males, triangles—females. All data are expressed as means ± SEM, *n* ≥ 12. ^*^p < 0^.^05, ^**^p < 0^.^01, ^***^p < 0^.^001.

All pups were also tested on the EPM test validated to assess anxiety-like or avoidance behavior in rodents (Walf and Frye, 2007). The number of entries into the open arms (%, Figure 12A) and the duration of time spent in the OA (%) were scored (Figure 12B). The data passed the normality test and was analyzed using two-way ANOVA followed by Tukey’s multiple comparisons test. Offspring of Abx dams had significantly less % entries in the OA: *F*(1,88) = 18^.^76 *p* < 0^.^0001 (row—treatment); *F*(1,88) = 4^.^512 *p* = 0^.^0365 (column—sex), and less % duration in the OA: *F*(1,74) = 38^.^59 *p* < 0^.^0001 (row—treatment); *F*(1,74) = 3^.^479 *p* = 0^.^0661 (column—sex) than their respective controls, indicating increased anxiety-like measures. No significant sex-specific differences were found between the groups on the EPM test.

Together, our results indicate sex-dependent differences in microbiome composition and predictive functionality, adrenal transcriptome profiles, basal urinary epinephrine levels and exploratory behavior in control weanling mice. Maternal antimicrobials resulted in significant microbiome disruption in the offspring and affected the transcriptional activity in the adrenal medullae, peripheral catecholaminergic pathways, responses to stress, anxiety-like behavior and locomotor activity in a sexually dimorphic manner.

## 4 Discussion

Initial gut colonization at birth serves a crucial role in the development of the enduring gut microbiome as well as affecting the host’s health over the entire life of the subject (Dierikx et al., 2020). Notwithstanding many clear immediate benefits, the risks from direct and indirect exposure of neonates to antibiotics extends beyond the spread of antibiotic resistance to include unintended and less clearly understood adverse effects related to gut dysbiosis and its impact on long-term wellbeing (Zimmermann and Curtis, 2019). The current study provides unequivocal evidence of the sex-specific effects of oral antibiotics given to dams from parturition, on gut microbial composition, predictive functionality, adrenal transcriptome, responses to acute insulin-induced hypoglycemia and anxiety-like behaviors in the offspring at weaning.

The use of antimicrobials or germ-free animals are complimentary tools valuable for exploring causality in the gut-brain axis and the impact of microbiome on different aspects of host physiology. While treatment with antibiotics offers the advantage to model common clinical exposures in humans by disrupting microbial composition during selected life periods [perinatal (Tochitani et al., 2016; Leclercq et al., 2017; Russell et al., 2013; Russell et al., 2012; O'Connor et al., 2021; Champagne-Jorgensen et al., 2020; Morel et al., 2023), early life (Clarke et al., 2014; Lynch et al., 2023), adolescence (Desbonnet et al., 2015; Lach et al., 2020), or in adulthood (Hoban et al., 2016; Frohlich et al., 2016; Bercik et al., 2011; Gacias et al., 2016; Mohle et al., 2016; Guida et al., 2018), and in old age animals (van Opstal et al., 2016)], the timing, duration, mode of delivery and type of antibiotics are important factors to consider when studying its influence on the gut microbiome. We are particularly interested in the early postnatal period as this is a critical time for both neuronal development (Zoghbi, 2003) and the initial seeding of the microbiota (Mutic et al., 2017), linked to development of the HPA axis, and stress response (Sudo et al., 2004; Foster et al., 2017; Davidson et al., 2018; Farzi et al., 2018). In addition, due to the high rates of antibiotic use in the postpartum period in both mother and infant (Morreale et al., 2023; Miyoshi and Hisamatsu, 2022; McDonnell et al., 2021), disturbance to the microbiota in early life has the potential to significantly impact brain and behavior in human offspring. Maternal antibiotic treatment in the perinatal period clearly affects gut colonization in the offspring by prenatal exposure to the fetus or changes in the vertical microbial transmission (not relevant in our case), through skin-to-skin contact with the mother, and through the breastmilk (Dominguez-Bello et al., 2010; Martin et al., 2003; Dierikx et al., 2020; Pacifici, 2006; Morreale et al., 2023; Zwittink et al., 2018).

The broad spectrum Abx cocktail used in this study was chosen based on previously published preclinical reports (by us and others—see LaGamma et al., 2021; Kiraly et al., 2016; Sampson et al., 2016; Reikvam et al., 2011), showing an overall gut microbiome depletion after 2 weeks of exposure in adult rodents with no systemic effects. While this specific antibiotic combination is not used in clinical settings, broad spectrum antimicrobials like vancomycin, ampicillin and gentamycin alone and/or in combination (included in our cocktail) are preferred clinical choices for empirical therapy in the NICU to cover a wide spectrum of potential pathogens in neonates with suspected sepsis or meningitis (Ting et al., 2022), validating the translational potential of our study. In our experiments, the significant disruption of the microbiome in the offspring of Abx drinking dams was confirmed by several independent criteria: (i) fecal WGS sequencing of individual samples from each experimental group revealed significantly reduced alpha- and altered beta diversity in the offspring of Abx treated dams (Figures 4A–C; Supplementary Figure 2); (ii) significantly enlarged ceca in the Abx offspring—a macroscopic hallmark for gut microbiome imbalance (Figure 2B); and (iii) no detectable products of bacterial fermentation (cecal SCFA) in the Abx groups; key bioactive metabolites in microbiota-gut-brain axis communication (O'Riordan et al., 2022). Our results are supportive of previously reported data (Russell et al., 2012; Leclercq et al., 2017; O'Connor et al., 2021) using a different strain of mice [BALB/C (Leclercq et al., 2017; Champagne-Jorgensen et al., 2020)], and a different time/length of exposure (Russell et al., 2012; O'Connor et al., 2021; Morel et al., 2023) including different combinations of antibiotics—vancomycin or streptomycin (Russell et al., 2012), low dose penicillin (Leclercq et al., 2017; Champagne-Jorgensen et al., 2020), single Abx [ampicillin (O'Connor et al., 2021)] or cocktail of Abx, including ampicillin, vancomycin, metronidazole, ciprofloxin and imipenem (O'Connor et al., 2021).

Alpha diversity is used to assess richness (number of species represented) and evenness (equality between species) within a given community. As in previously reported studies using a similar experimental paradigm (Russell et al., 2012; Leclercq et al., 2017), we observed significantly reduced microbial richness in Abx groups and in their predictive functionality compared to their respective controls. Beta diversity comparison (Bray-Curtis distance matrix, which takes abundance into account) also identified significant differences in the overall composition between control and Abx groups and in their predictive metabolic activity, similar to previous reports of antibiotic treatments in other preclinical models (Lach et al., 2020) such as adolescent mice (Lynn et al., 2021; Desbonnet et al., 2015; Leclercq et al., 2017; Lynch et al., 2023), or in human children (aged from 0 to 18 years) (McDonnell et al., 2021).

Maternal Abx exposure markedly altered microbiome taxonomic profiles in both male and female offspring. Using WGS sequencing data, we identified 128 strains from 53 genera, 35 families and 8 phyla significantly altered in the offspring of Abx exposed dams. The Abx groups exhibited notable shifts at the phylum level in Firmicutes (Bacillota) and Bacteroidetes (Bacteroidota), the major bacterial phyla in the murine (and human) intestinal microbiome (Shah et al., 2021; Qin et al., 2010). In this regard, higher abundance of phylum Firmicutes (Tomova et al., 2015) and Proteobacteria was found in children with ASD (Abuljadayel et al., 2024). Animal studies revealed that the lipopolisaccharides produced by Proteobacteria result in reduced levels of gluthatione in the brain and immune dysregulation, suggesting possible link of oxidative stress mechanisms and clinical symptoms in individuals with ASD (Zhu et al., 2007; Chauhan and Chauhan, 2006). Several Bacilli members [e.g., *Streptococcus thermophilus TH1435* (Martinovic et al., 2020), *Lactobacillus kitasatonis, Leuconostoc Mesenteroides* etc. exhibited significantly increased relative abundance in Abx cohorts. Similarly, children with ASD also have higher levels of Bacilli associated with GI symptoms (diarrhea, nausea, vomiting and abdominal pain) (Abuljadayel et al., 2024)]. The effects on *Clostridia* (uses organic acids to generate additional SCFA) (Pickard and Nunez, 2019) and other *Bacilli* members migrated in the opposite direction (decrease in relative abundance) in our study, including *Roseburia, Ligilactobacillus animalis* and *L. murinus, Lactobacillus johnsonnii* like the data obtained from ASD patient samples (Abuljadayel et al., 2024).

Phylum Bacteroidetes (aerobic, anaerobic, gram-negative bacteria, primary fermenters transforming sugars from complex carbohydrates to organic acids and SCFA) displayed markedly reduced relative abundance in Abx cohorts, consistent with the observed lack of detectable SCFA in the offspring of Abx dams. Notably, decreased levels of this phylum cause mucosal dysbiosis in the gut and abnormal digestion of carbohydrates in kids with ASD (rev. in Gyawali and Patra, 2019). Despite the inconsistent taxonomic shifts reported across studies (Lynch et al., 2023; Nyangahu et al., 2018; Russell et al., 2012; Leclercq et al., 2017; O'Connor et al., 2021; Kayyal et al., 2020; Clarke et al., 2014) gut microbial dysbiosis in the offspring of Abx exposed dams is a common finding. Although most preclinical research in this field has been performed in male animals yet reports of sex-dependent differences are emerging (Org et al., 2016; Gao et al., 2021; Vemuri et al., 2019; Kayyal et al., 2020; Poon et al., 2022; Morel et al., 2023).

In view of the complex systems biology of sex specific differences, we expanded our study in weanling mice, and it revealed strain-level sex differences between microbiomes in offspring of both, control dams and Abx exposed dams, reflected in their predictive metabolic activities. These findings indicate that sex differences in the gut microbiome composition emerge before puberty in contrast with the previous belief (Yurkovetskiy et al., 2013; Sisk and Foster, 2004). Of particular relevance is that even after a human birth (first defecation sample) males were found to have higher total bacterial counts than females, while females had higher gut *B. fragilis* and *Lactobacillus* spp. than males (Martin et al., 2016), indicating that sexual dimorphism in the initial gut colonization exists in humans as well as in animal models. Collectively the data illustrates different colonization dynamics and metabolic activity exists between sexes (Supplementary Figures 1A,B), and that it can impact future biological functions. In support of this interpretation are observations that communication along the microbiota-gut-brain axis is also reported to be sexually dimorphic, and to contribute to the development of distinct microbial communities, immune signaling pathways, and neuroinflammatory processes in males and females, ultimately giving rise to different mental health phenotypes (McCarthy et al., 2017; Jasarevic et al., 2016; Audet, 2019; Coretti et al., 2017).

In our experimental model we also found sex-dependent differences in host physiological responses in control groups vs. offspring of Abx-exposed dams, suggesting distinct gut microbiome phenotypes may be affecting those reactions even after normal birth. In support of this interpretation, we found that basal urinary epinephrine levels were significantly higher in female offspring of control dams compared to male offspring, consistent with the previously reported higher levels of sympathoadrenal activity and plasma catecholamines in females (Weinstock et al., 1998). These asymmetrical sex-specific findings are further corroborated by our AM RNA sequencing data analyses showing significantly up-regulated DEGs encoding connexins (Gja1, Gjb2), Ocln and CNST [integral membrane protein, binding partner of connexins (del Castillo et al., 2010)] in females. The RNAseq data taken together with evidence of more gap junction channels present in chromaffin cells of females (Martin et al., 2001) compared to males (Colomer et al., 2008), suggest a role for gap junctional communication in the sex-specific regulation of basal circulating/urinary catecholamine concentrations; yet to be proven.

There are additional effects of gut microbiome disruption that resulted in higher basal epinephrine levels in Abx cohorts, especially for female offspring. This phenomenon was also correlated with altered expression of several molecules (Elfn, Lrrn1, Chrm2, Chmr3, Htr3a, Htr3b, Gria1, Nos2, Adrb2, Adra2a) and the enrichment in predictive canonical pathways in Abx offspring, all also related to gap junction trafficking, regulation and signaling (Syt2, Napb, Nod1, Gja1, Gja2, Ocln, CNST, Oxtr) in a sex-specific manner. To the best of our knowledge, we are first to report sexually dimorphic transcriptome profiling data of adrenal medullae in prepubertal controls and mice with gut dysbiosis and in relating this observation with gap junctions. Of note, the adrenal cortex transcriptomes of male and female prepubertal mice are also sexually dimorphic and respond differently to the same external stimulus (triiodothyronine) (Lyu et al., 2020).

Another interpretation of our results is that the different basal epinephrine levels may stem from sex-specific differences in the urinary creatinine levels, as noted previously in healthy newborns commonly related to muscle mass rather than glomerular filtration (Lopez-Hernandez et al., 2020). We do not subscribe to that interpretation as there was neither significant difference in weight at weaning (consistent with similar muscle mass) nor in the detected urinary creatinine levels in our study. Alternatively, a neurohumoral based developmental pattern may exist to explain these differences since it is already recognized that the vagus nerve has both sex- and time-specific changes in its effects after birth (Cerritelli et al., 2021). Given that afferent vagal pathways contribute to the negative feedback regulation of sympathoadrenal system (Mravec et al., 2015), and the regulatory patterns of sympathovagal balance show sexual dimorphism (Goncalves et al., 2017; Koenig et al., 2017), these phenomena may account for (or at least contribute to) the observed sex differences in basal urinary epinephrine levels in our study. Further experiments are needed to defend this consideration.

To determine whether the changes in gut flora can impact specific nervous system functions before puberty, we tested the effect of maternal antibiotic use on a well characterized reflex arc in their offspring—epinephrine release in response to insulin-induced hypoglycemia. What is novel from the current study is that when compared to controls, exposure to insulin-induced hypoglycemia resulted in even higher epinephrine release in the female Abx group (Figure 8C), while the ability to respond to hypoglycemia in male Abx offspring was significantly reduced (Figure 8B). This phenomenon is identical to our publications reporting on male adult GF and Abx mice (Giri et al., 2019; LaGamma et al., 2021).

Taken together with our data showing significant decreases in the relative abundance of SCFA-producing microbial taxa after Abx in both female and male offspring, we conclude that other factors/processes or mechanism(s) must exist to support differences in adrenal responses to hypoglycemic stress during gut dysbiosis in females. Based on the novel findings above, perhaps gap junction signaling is a contributing system (Berends et al., 2019; Desarmenien et al., 2013). Since several reports describe the use of vagal afferents by gut microbiota/microbial metabolites to modify the host’s behavioral and emotional responses, we speculate that redundant overlapping neuronal processes emerged through evolution to enhance survival responses as a reasonable teleologic interpretation to explain these observations (Goehler et al., 2005; Lyte et al., 1998; Bravo et al., 2011; Sgritta et al., 2019; Bercik et al., 2011; Han et al., 2022).

While we are clearly speculating on the relevance and complexity of interpreting even a single reflex arc as a model for understanding the impact of dysbiosis, we argue that it represents a more important strategy for gaining mechanistic insights into neuronal signaling in more complex nervous system functions such as behavior. Significantly, in our model we observed sex-dependent effects on the anxiety-related behavioral outcomes and exploratory behavior in the offspring at weaning with and without microbiome perturbance: for example, female pups from control group traveled significantly less total distance on the OF test, compared to males, and female Abx pups spent significantly less time in the center (OF) than male Abx.

Collectively, our observations are consistent with the well-reasoned and accepted concept that gut bacteria can impact neurological outcomes—altering complex behaviors relevant to mood, pain and cognition, or responses to physiologic stress (hypoglycemia), potentially affecting the onset and/or severity of future nervous system disorders beginning from birth (Sampson and Mazmanian, 2015; Borre et al., 2014b; Bruce-Keller et al., 2017; Diaz Heijtz, 2016; Kayyal et al., 2020; Champagne-Jorgensen et al., 2020; O'Connor et al., 2021; Morel et al., 2023; Lynch et al., 2023).

### 4.1 Limitations of the study

There are some limitations in the study worthwhile discussing: (1) Abx-induced changes in maternal microbiome were not evaluated in the study; (2) Our analyses were based on fecal WGS sequencing (the non-adherent bacteria in the stool) and are not necessarily representative of mucosa associated microbiota (Donaldson et al., 2016); (3) We also did not analyze the stomach content in the offspring (proxy for the breast milk microbiota); (4) Adrenal medullary transcriptome profiles are based on polyA RNA sequencing in 3 biological replicates/group. Potential changes in protein levels or post-translational modifications have not been examined yet.

Given the observational design of the study, our data enabled the identification of associative (and not necessarily causative) sex-dimorphic effects of maternal Abx exposure on microbiome composition, predictive functionality and metabolic activity, adrenal medullary transcriptomes, stress responsiveness and behavioral outcomes in the pre-pubescent offspring. Future experiments will be performed to delineate hypothesized mechanism(s) to acquire new knowledge that can help develop therapeutic measures to alter nervous system functions and behavior.

We conclude that maternal oral antimicrobials dictate neonatal microbiome disruption in weanling mice in a sexually dimorphic manner, associated with altered host adrenal medullary transcriptome, locomotor activity, anxiety-like behavior and peripheral catecholaminergic pathways/responses to stress. The broad implications of these findings portend pragmatic consequences for patient care regarding overuse of antibiotics, repopulation of gut flora and developing of sex-specific nutritional and therapeutic strategies for maintenance of successful stress adaptations and promotion of host well-being.

## Data Availability

The whole genome shotgun sequencing data are deposited in NCBI SRA (BioProject ID PRJNA1098026). All other data supporting the findings of the study are available in the paper and Supplementary materials, or from the corresponding author upon request.

## Footnotes

^1^ https://app.cosmosid.com
