# Supplementary material for: Early life microbiome disbalance impacts neuroendocrine outcomes in pre-pubertal mice in a sexually dimorphic manner
Source: Front Microbiol. 2025 Jun 20;16:1504513. doi: 10.3389/fmicb.2025.1504513 (PMC12277575; doi:10.3389/fmicb.2025.1504513)
Supplement: Supplementary file 1 [file Supplementary_file_1.zip › Supplementary Figures 1-4.DOCX]

**
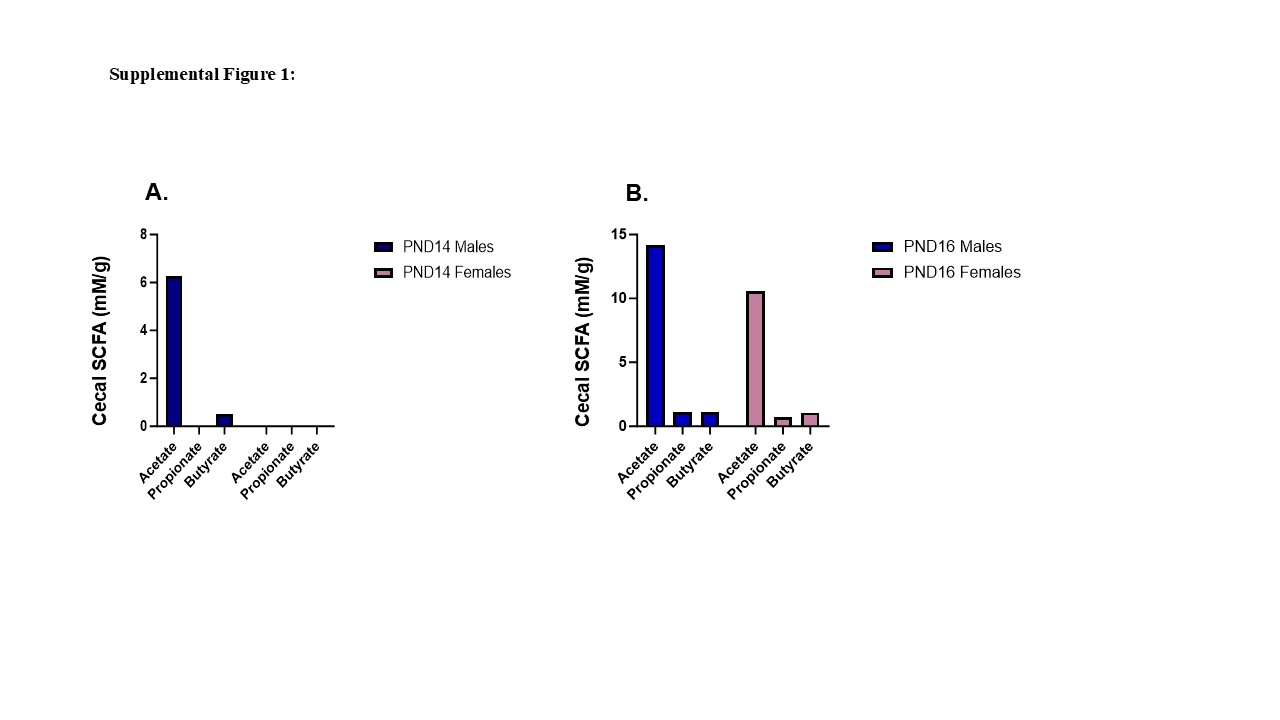
**

**Supplemental figure 1:** Pooled cecal samples from control male and female offspring at PND 14 (A) and PND 16 (B) were used to isolate and quantify SCFA as described in methods.

**
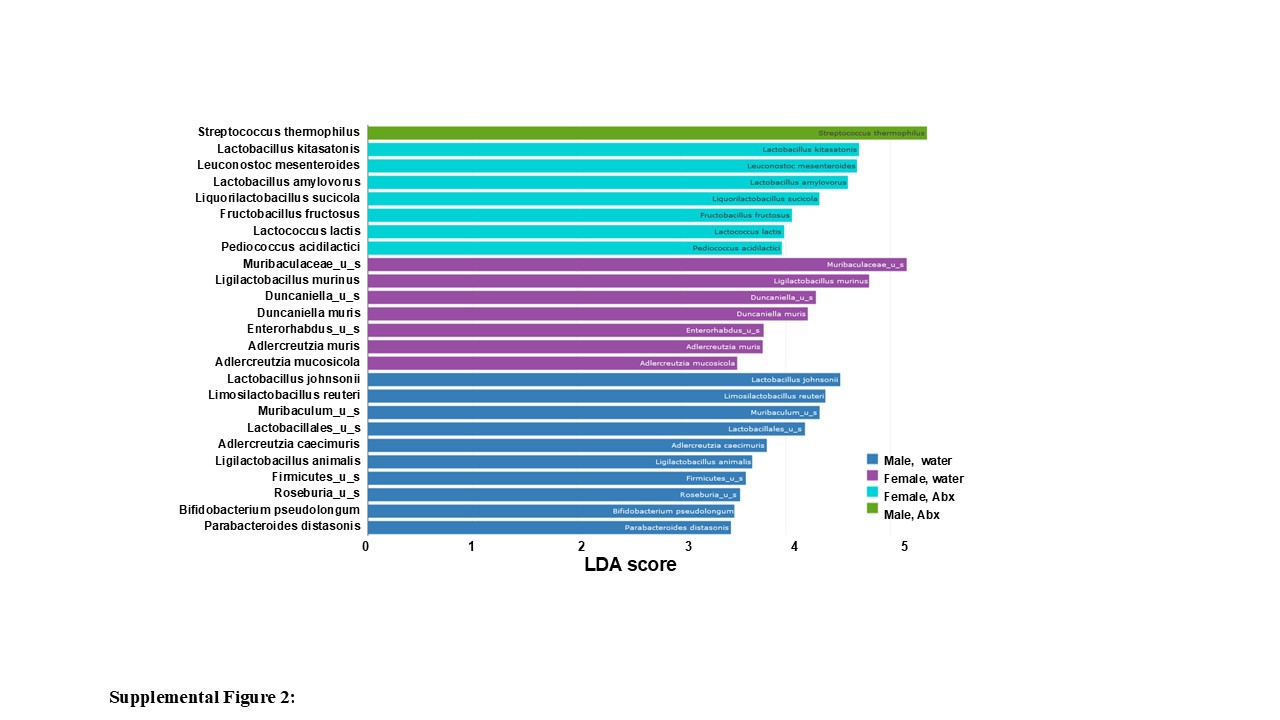
**

**Supplemental figure 2: Graphic plot of linear discriminant analysis** (**LDA) scores of multilevel comparisons of taxa across the 4 experimental groups using CosmosID-Hub:** Strain level **e**nrichment, logarithmic threshold ≥ 2, P < 0.05). Groups: 1- male, water (blue); 2- male, Abx (green); 3 – female, water (purple); 4 – female, Abx (turquoise).

**
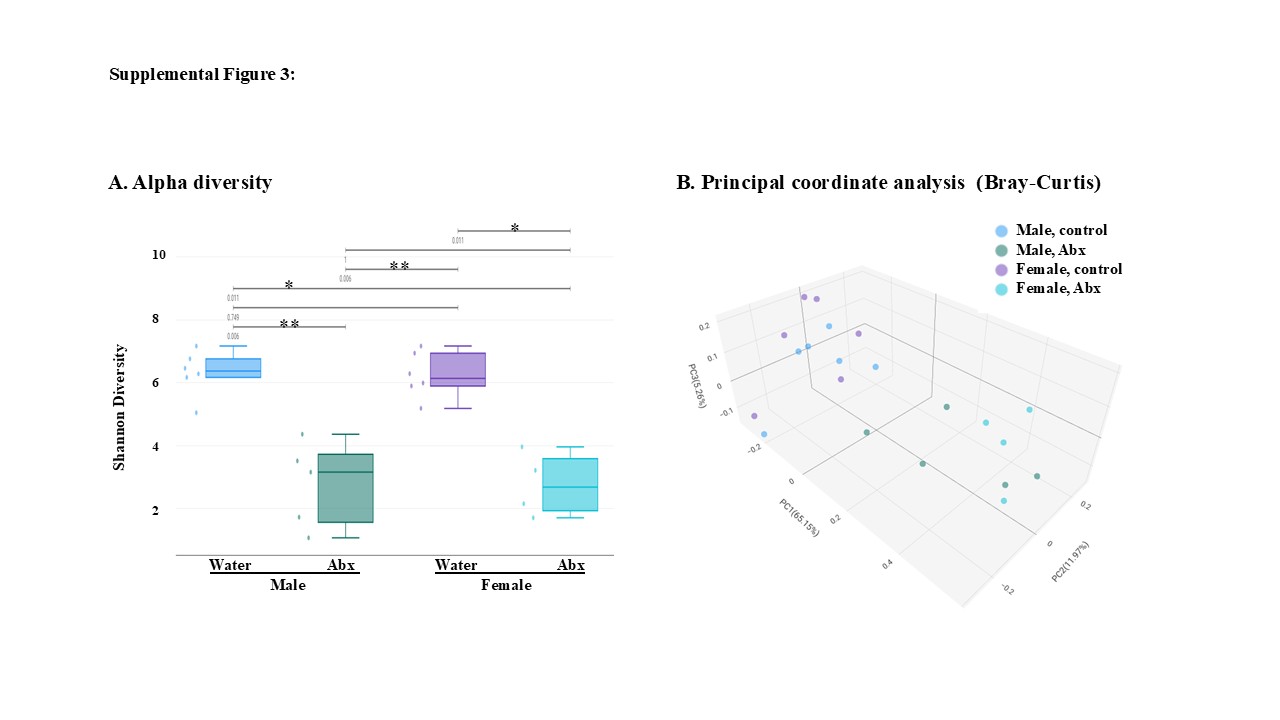
**

**Supplemental figure 3: Predictive metabolic activities from WGS sequencing** - metabolic pathways enrichment analysis based on MetaCyc database. Groups: 1- male, water (blue); 2- male, Abx (green); 3 – female, water (purple); 4 – female, Abx (turquoise). Each dot represents value for an individual animal, n ≥ 4. **A**) Alpha diversity represented by Shannon index (richness and evenness). Boxplots show 25th and 75th percentiles with a line at the median. Wilcoxon rank sum test, **p ≤ 0.006; *p ≤ 0.05. **B**) Beta diversity (Bray-Curtis) principal coordinate analysis (PERMANOVA, all **p = 0.001), indicating distinct clustering and separation of Abx and

water groups.

**
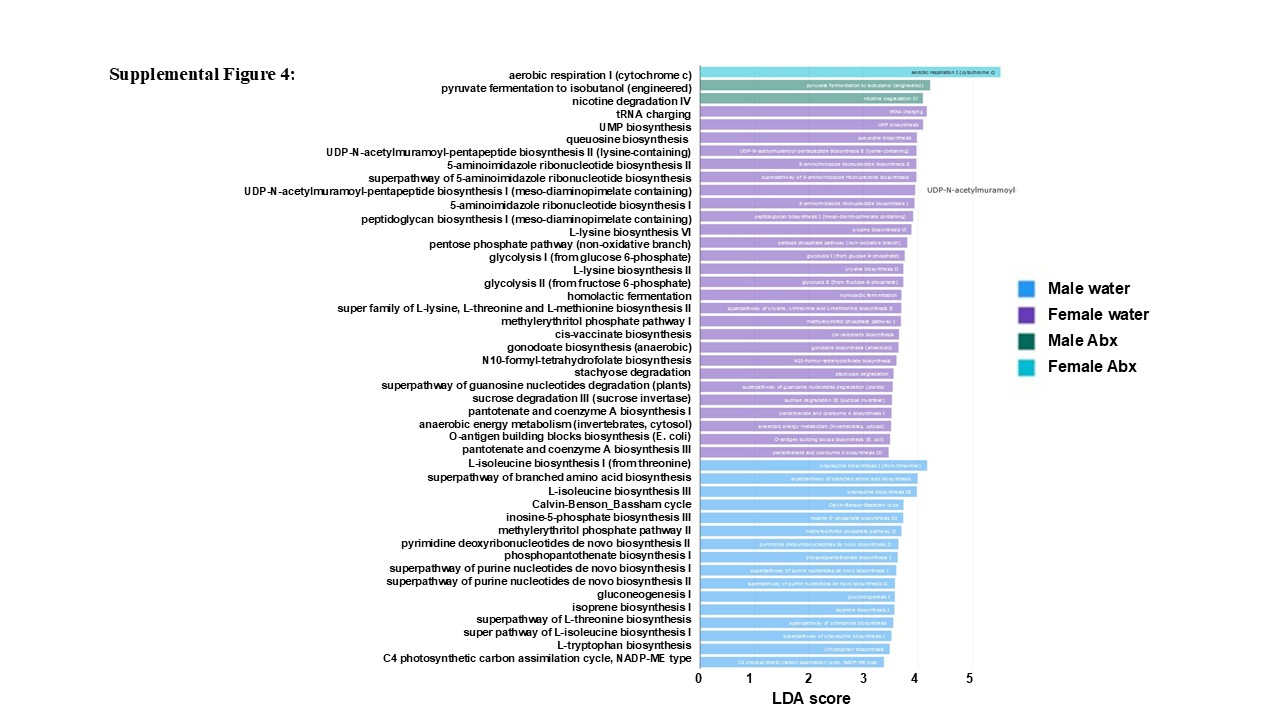
**

**Supplemental figure 4:** Graphic plot of LDA scores of multilevel comparisons of predictive metabolic pathways across the 4 experimental groups using CosmosID-Hub: The LDA score from LEfSe analysis were calculated with a Kruskal-Wallis alpha value of 0.05, a Wilcoxon alpha value of 0.05, and a logarithmic LDA score threshold of 2.0. The metabolic pathways enrichment analysis was based on MetaCyc database between groups (see Methods).
