# Supplementary material for: Early life microbiome disbalance impacts neuroendocrine outcomes in pre-pubertal mice in a sexually dimorphic manner
Source: Front Microbiol. 2025 Jun 20;16:1504513. doi: 10.3389/fmicb.2025.1504513 (PMC12277575; doi:10.3389/fmicb.2025.1504513)
Supplement: Supplementary file 1 [file Supplementary_file_1.zip › Supplementary File Captions.DOCX]

The supplementary materials include the following.

**Figures:**

**Supplemental figure 1:** Delayed initial colonization and different microbial activity in control female offspring

**Supplemental figure 2**: Graphic plot of linear discriminant analysis (LDA) scores of multilevel comparisons of taxa across the 4 cohorts

**Supplemental figure 3**: Predictive metabolic activities from WGS sequencing - metabolic pathways enrichment analysis

**Supplemental figure 4:** LDA scores of predictive metabolic pathways comparisons between the 4 experimental groups

**Tables:**

**Supplemental table 1:** Taxonomic profiles - strain level relative abundances in control and Abx groups

**Supplemental table 2:** LDA effect size (LEfSe) analysis of the bacterial strain differences between the experimental groups

**Supplemental table 3:** LEfSe table – predictive functional analysis, metabolic pathways

**Supplemental table 4:** DEG in adrenal medullae of male offspring (male control vs male Abx, log2 FC=1)

**Supplemental table 5:** DEG in adrenal medullae of female offspring (female control vs female Abx, log2 FC=1)

**Supplemental table 6:** Sexually dimorphic DEG in adrenal medullae of control offspring (female vs male, Log2 FC=2

**Supplemental table 7:** Sexually dimorphic DEG in adrenal medullae of Abx weanlings (female vs male, Log2 FC=2
