# Supplementary material for: Early life microbiome disbalance impacts neuroendocrine outcomes in pre-pubertal mice in a sexually dimorphic manner
Source: Front Microbiol. 2025 Jun 20;16:1504513. doi: 10.3389/fmicb.2025.1504513 (PMC12277575; doi:10.3389/fmicb.2025.1504513)
Supplement: Supplementary file 1 [file Supplementary_file_1.zip › Supplementary Table 1.DOCX]

**Supplemental Table 1: Taxonomic profiles - strain level relative abundances in control and Abx groups**

| **Strain** | **Male Water** | **Male Abx** | **Female Water** | **Female Abx** | **Taxonomy ID** |
| --- | --- | --- | --- | --- | --- |
|  | (avg) | (avg) | (avg) | (avg) |  |
| Acetatifactor muris | 0.002885 | 0.00333 | 0.002416 | 0 | 879566 |
| Acutalibacter sp. 1XD8-33 | 0.001538 | 0.001248 | 0.00082 | 0 | 2320081 |
| Adlercreutzia muris | 0.008697 | 0 | 0.011075 | 0 | 1796610 |
| Akkermansia muciniphila | 0.0032517 | 0 | 0 | 0 | 239935 |
| Akkermansia muciniphila ATCC BAA-835 | 0.017993 | 0.00334 | 0.017247 | 0 | 349741 |
| Akkermansia sp. KLE1798 | 0.0120983 | 0 | 0.00737 | 0 | 1574265 |
| Bacteria_u_t | 0.000788 | 0 | 0 | 0 | 2 |
| Alistipes finegoldii DSM 17242 | 0.0016433 | 0 | 0.00073 | 0 | 679935 |
| Alistipes sp. HGB5 | 0.0018183 | 0 | 0.000812 | 0 | 908612 |
| Anaerotruncus sp. G3(2012) | 0.012043 | 0.01181 | 0.0150417 | 0.00243 | 1235835 |
| Adlercreutzia equolifaciens subsp. celatus | 0.0002367 | 0 | 7.66667E-05 | 0 | 394340 |
| Bacteroides_u_t | 0.001253333 | 0 | 0.000595 | 0 | 816 |
| Bacteroides acidifaciens JCM 10556 | 0.001131667 | 0 | 0.00054 | 0 | 1235814 |
| Bacteroides caccae | 0.0001433 | 0 | 0 | 0 | 47678 |
| Bacteroides caecimuri | 3.66667E-05 | 0 | 0.00004 | 0 | 2516958 |
| Bacteroides caecimuris | 4.33333E-05 |  | 0.000237 | 0 | 1796613 |
| Bacteroides fragilis | 0.000116667 | 0 | 0 | 0 | 817 |
| Bacteroides fragilis str. 3397 T10 | 3.83333E-05 | 0 | 0 | 0 | 1339284 |
| Bacteroide massiliensis B84634 | 0.00152 | 0 | 0 | 0 | 1121098 |
| Bacteroides ovatus ATCC 8483 | 0.0004233 | 0 | 0.000168 | 0 | 411476 |
| Bacteroides ovatus SD CMC 3f | 0 | 0 | 0.005791667 | 0 | 702443 |
| Bacteroides sp. AF33-23 | 0.001675 | 0 | 0.000838 | 0 | 2292930 |
| Bacteroides sp. AM16-15 | 0.001863 | 0 | 0 | 0 | 2292939 |
| Bacteroides sp. OF03-11BH | 0 | 0 | 0.006493333 | 0 | 2292957 |
| Bacteroides uniformis dnLKV2 | 0.018621667 | 0.006118 | 0.005825 | 0.003445 | 1235787 |
| Bifidobacterium pseudolongum AGR2145 | 0.003875 | 0 | 0 | 0 | 1280701 |
| Bifidobacterium pseudolongum PV8-2 | 0.000511667 | 0 | 0 | 0 | 1447715 |
| Blautia sp. YL58 | 0.000203333 | 0 | 0.000117 | 0 | 1796616 |
| Burkholderiales_u_t | 0 | 0 | 0.001042 | 0 | 80840 |
| Butyricimonas virosa DSM 23226 | 0.000168333 | 0 | 0.00028 | 0 | 1121130 |
| Candidatus Arthromitus_u_t | 0 | 0.0012 | 0.000163 | 0.001523 | 49082 |
| Candidatus Arthromitus sp. SFB-mouse-Japan | 0.0038517 | 0 | 0.0031733 | 0 | 1029718 |
| Carnobacterium inhibens subsp. gilichinskyi | 0 | 0 | 0 | 0.004205 | 1266845 |
| Carnobacterium sp. WN1374 | 0 | 0 | 0 | 0.0026075 | 1266850 |
| Clostridium_u_t | 0 | 0 | 0 | 0.0022875 | 1485 |
| [Clostridium] cocleatum | 0.000808 | 0 | 0.0016233 | 0 | 69824 |
| Clostridium cuniculi | 0 | 0 | 0.00014 | 0 | 2548455 |
| Clostridium sp. MD294 | 0 | 0 | 0.00014 | 0 | 97138 |
| Schaedlerella arabinosiphila | 0.0184433 | 0.019866 | 0.004827 | 0.00816 | 2044587 |
| Curtobacterium_u_t | 0 | 0 | 0 | 0.0028175 | 2034 |
| Dorea sp. 5-2 | 0.003468333 | 0.009832 | 0.002718 | 0.004483 | 1235798 |
| Duncaniella muris | 0.0103717 | 0 | 0.02742 | 0 | 2094150 |
| Duncaniella sp. B8 | 0.012273333 | 0 | 0.0337117 | 0 | 2576606 |
| Duncaniella freteri | 0.036295 | 0.012966 | 0.029098 | 0.005843 | 2530391 |
| Eggerthella lenta | 0 | 0 | 0.000363333 | 0 | 84112 |
| Eggerthella sp. 1_3_56FAA | 0 | 0 | 0.000773333 | 0 | 665943 |

| Enterococcus_u_t | 0.00193 | 0 | 0.002095 | 0 | 1350 |
| --- | --- | --- | --- | --- | --- |
| Enterococcus faecium |  | 0.01043 |  | 0 | 1352 |
| Enterorhabdus caecimuris B7 | 0.0119783 | 0 | 0.008295 | 0 | 1235794 |
| Enterorhabdus mucosicola DSM 19490 | 0.000365 | 0 | 0.003478 | 0 | 1121866 |
| Enterorhabdus sp. NM05_H27 | 0.0091183 | 0 | 0.011305 | 0 | 2516956 |
| Escherichia coli SWW33 | 0 | 0 | 0 | 0.002 | 1235805 |
| Escherichia coli | 0.00526 | 0.002258 | 0.002425 | 0 | 562 |
| Eubacterium plexicaudatum ASF492 | 0.002145 | 0.00331 | 0.00094 | 0 | 1235802 |
| Eubacterium sp. 14-2 | 0.003425 | 0.005964 | 0.000488 | 0 | 1235790 |
| Faecalibaculum rodentium | 0.00035 | 0 | 0.000173 | 0 | 1702221 |
| Firmicutes bacterium ASF500 | 0.005546667 | 0.005018 | 0.004497 | 0 | 1378168 |
| Fructobacillus fructosus KCTC 3544 | 0 | 0.004636 | 0 | 0.023678 | 930946 |
| Glutamicibacter sp. BW77 | 0 | 0 | 0 | 0.024925 | 2024402 |
| Lachnospiraceae bacterium 10-1 | 0.013643333 | 0.0107733 | 0.002458 | 0 | 1235800 |
| Lachnospiraceae bacterium 28-4 | 0.016691667 | 0.00945 | 0.035083 | 0 | 397287 |
| 0.0050333+AA64 | 0.003881667 | 0.005108 | 0.002538 | 0 | 397288 |
| Lachnospiraceae bacterium MD308 | 0.003411667 | 0.007912 | 0.00153 | 0.00229 | 1235799 |
| Lachnospiraceae bacterium A2 | 0.007398333 | 0.004462 | 0.00952 | 0.011253 | 397290 |
| Lachnospiraceae bacterium A4 | 0.06357 | 0.127682 | 0.0374983 | 0.028695 | 397291 |
| Lachnospiraceae bacterium MD335 | 0.039351667 | 0.05802 | 0.027165 | 0.013085 | 1235793 |
| Lachnospiraceae bacterium M18-1 | 0.004501667 | 0 | 0.0050333 | 0 | 1235792 |
| Lactobacillaceae bacterium | 0.031111667 | 0 | 0.0271883 | 0 | 2291323 |
| Ligilactobacillus_u_t | 0 | 0.005164 | 0 | 0 | 2767887 |
| Lactobacillus amylovorus | 0 | 0.031326 | 0 | 0.063795 | 1604 |
| Lactobacillus amylovorus GRL1118 | 0 | 0.00965 | 0 | 0.0162225 | 695562 |
| Ligilactobacillus animalis | 0.00108333 | 0 | 0.000685 | 0 | 1605 |
| Lactobacillus delbrueckii subsp. lactis | 0 | 0 | 0 | 0.00452 | 29397 |
| Lactobacillus johnsonii N6.2 | 0.06716 | 0.004958 | 0.039783 | 0.001418 | 1408186 |
| Lactobacillus johnsonii NCC 533 | 0 | 0 | 0.003535 | 0 | 257314 |
| Lactobacillus kitasatonis DSM 16761 = JCM 1039 | 0 | 0.03103 | 0 | 0.100348 | 1423767 |
| Lactobacillus murinus ASF361 | 0.132023333 | 0.00558 | 0.133678333 | 0.034313 | 1235801 |
| Limosilactobacillus reuteri | 0 | 0 | 0 | 0.0025 | 1598 |
| Lactobacillus reuteri TD1 | 0.019333 | 0 | 0 | 0 | 1358027 |
| Limosilactobacillus reuteri subsp. murium | 0.0328317 | 0.003952 | 0.035968 | 0.002235 | 863368 |
| Lactobacillus reuteri mlc3 | 0 | 0 | 0.003343333 | 0 | 863369 |
| Lactobacillus sp. ASF360 | 0.037216667 | 0.001236 | 0.033305 | 0 | 97137 |
| Lactobacillus sp. OTU4228 | 0 | 0.007234 | 0 | 0.030478 | 1572760 |
| Lactobacillus sp. UMNPBX19 | 0 | 0 | 0.000901667 | 0 | 2042028 |
| Lactobacillus sucicola DSM 21376 = JCM 15457 | 0 | 0.017974 | 0 | 0.043005 | 1423806 |
| Lactococcus lactis subsp. cremoris | 0 | 0.002304 | 0 | 0.01598 | 1359 |
| Lactococcus lactis subsp. lactis | 0 | 0.002172 | 0 | 0 | 1360 |
| Leuconostoc_u_t | 0 | 0.001768 | 0 | 0 | 1243 |
| Leuconostoc mesenteroides | 0 | 0.013454 | 0 | 0.042055 | 1245 |
| Leuconostoc mesenteroides subsp. mesenteroides ATCC 8293 | 0 | 0.00663 | 0 | 0.053685 | 203120 |
| Mucispirillum schaedleri ASF457 | 0.032671667 | 0.052416 | 0.030018 | 0.005063 | 1379858 |
| Muribaculaceae bacterium Isolate-001 (NCI) | 0.005495 | 0 | 0.0089967 | 0 | 2489214 |
| Muribaculaceae bacterium Isolate-004 (NCI) | 0.000708333 | 0 | 0.001032 | 0 | 2489216 |
| Muribaculaceae bacterium Isolate-007 (NCI) | 0.0063717 | 0 | 0.013825 | 0 | 2489217 |

| Muribaculaceae bacterium Isolate-013 (NCI) | 0.000235 | 0 | 0.0382317 | 0.02251 | 2489218 |
| --- | --- | --- | --- | --- | --- |
| Muribaculaceae bacterium Isolate-036 (Harlan) | 0.005501667 | 0 | 0.008877 | 0 | 2486460 |
| Muribaculaceae bacterium Isolate-037 (Harlan) | 0.02164 | 0 | 0.0727567 | 0.031905 | 2486461 |
| Muribaculaceae bacterium Isolate-039 (Harlan) | 0.009856667 | 0 | 0.024183 | 0 | 2486466 |
| Muribaculaceae bacterium Isolate-042 (Harlan) | 0.004906667 | 0 | 0.011402 | 0 | 2486462 |
| Muribaculaceae bacterium Isolate-043 (Harlan) | 0.0002 | 0 | 0.000055 | 0 | 2486463 |
| Muribaculaceae bacterium Isolate-077 (Janvier) | 0.004433333 | 0 | 0.00062 | 0 | 2486467 |
| Muribaculaceae bacterium Isolate-080 (Janvier) | 0.000155 | 0 | 0.000212 | 0 | 2486468 |
| Muribaculaceae bacterium Isolate-083 (Janvier) | 0.0085783 | 0 | 0.0265383 | 0 | 2486464 |
| Muribaculaceae bacterium Isolate-100 (HZI) | 0.00584 | 0 | 0.014297 | 0 | 2486469 |
| Muribaculaceae bacterium Isolate-102 (HZI) | 0.05135 | 0.009188 | 0.0167267 | 0.00199 | 2486470 |
| Muribaculaceae bacterium Isolate-104 (HZI) | 0.001273333 | 0 | 0.001488 | 0 | 2486471 |
| Muribaculaceae bacterium Isolate-105 (HZI) | 0.0020933 | 0 | 0.001768 | 0 | 2486472 |
| Muribaculaceae bacterium Isolate-110 (HZI) | 0.003675 | 0 | 0.01871 | 0 | 2486473 |
| Muribaculaceae bacterium Isolate-113 (HZI) | 0.005125 | 0 | 0.008105 | 0 | 2486474 |
| Muribaculaceae bacterium Isolate-114 (HZI) | 0.005045 | 0 | 0.008143 | 0 | 2486475 |
| Muribaculum intestinale | 0.004933333 | 0 | 0.01253 | 0 | 1796646 |
| Muribaculum sp. NM65_B17 | 0.040011667 | 0.005868 | 0.014057 | 0 | 2516961 |
| Oscillibacter sp. 1-3 | 0.016174 | 0.011586 | 0.00503 | 0.001425 | 1235797 |
| Parabacteroides distasonis | 0.00133 | 0 | 0.001005 | 0 | 823 |
| Pediococcus acidilactici | 0 | 0.006586 | 0 | 0.007055 | 1254 |
| Pediococcus acidilactici AGR20 | 0 | 0 | 0 | 0.0083125 | 1384067 |
| Clostridioides difficile | 0.000285 | 0 | 0.0003017 | 0 | 1496 |
| Clostridioides difficile F501 | 0 | 0 | 0.000746667 | 0 | 1151372 |
| Peptostreptococcus russellii | 0 | 0 | 0 | 0.0028 | 215200 |
| Prevotella sp. MGM1 | 0.069156667 | 0.019462 | 0.0437883 | 0.010963 | 2033405 |
| Roseburia sp. 1XD42-69 | 0.002156667 | 0.001718 | 0.00132 | 0 | 2320088 |
| Mammaliicoccus lentus F1142 | 0.000085 | 0 | 0.000485 | 0 | 1179226 |
| Staphylococcus xylosus | 0.000133333 | 0 | 7.16667E-05 | 0 | 1288 |
| Streptococcus_u_t | 0 | 0.00897 | 0 | 0 | 1301 |
| Streptococcus thermophilus | 0 | 0 | 0 | 0.0883 | 1308 |
| Streptococcus thermophilus LMD-9 | 0 | 0.043 | 0 | 0 | 322159 |
| Streptococcus thermophilus MN-ZLW-002 | 0 | 0 | 0 | 0.076 | 1187956 |
| Streptococcus thermophilus TH1435 | 0 | 0.058 | 0 | 0 | 1415776 |
| Streptococcus thermophilus TH1436 | 0 | 0 | 0 | 0.19 | 1423145 |
