# Supplementary material for: Early life microbiome disbalance impacts neuroendocrine outcomes in pre-pubertal mice in a sexually dimorphic manner
Source: Front Microbiol. 2025 Jun 20;16:1504513. doi: 10.3389/fmicb.2025.1504513 (PMC12277575; doi:10.3389/fmicb.2025.1504513)
Supplement: Supplementary file 1 [file Supplementary_file_1.zip › Supplementary Table 2 & 3.DOCX]

**Supplemental Table 2: LDA effect size (LEfSe) analysis of the bacterial strain differences between the groups**

| **Phylum** | **Strain** | **Enriched cohort** | **LDA Score** | **P-value** |
| --- | --- | --- | --- | --- |
| Firmicutes | Streptococcus Thermophilus | 2 | 5^.^35 | 0^.^0004 |
| Bacteroidetes | Muribaculaceae_u_s | 3 | 5^.^16 | 0^.^032 |
| Firmicutes | Ligilactobacillus murinus | 3 | 4^.^79 | 0^.^0027 |
| Firmicutes | Lactobacillus kitasatonis | 4 | 4^.^7 | 0^.^0011 |
| Firmicutes | Leuconostoc mesenteroides | 4 | 4^.^68 | 0^.^0011 |
| Firmicutes | Lactobacillus amylovorus | 4 | 4^.^59 | 0^.^0009 |
| Firmicutes | Lactobacillus johnsonii | 1 | 4^.^52 | 0^.^0044 |
| Firmicutes | Limosilactobacillus reuteri | 1 | 4^.^38 | 0^.^0014 |
| Bacteroidetes | Muribaculum_u_s | 1 | 4^.^32 | 0^.^0099 |
| Firmicutes | Liquorilactobacillus sucicola | 4 | 4^.^32 | 0^.^0016 |
| Bacteroidetes | Duncaniella_u_s | 3 | 4^.^29 | 0^.^003 |
| Bacteroidetes | Duncaniella muris | 3 | 4^.^21 | 0^.^0028 |
| Firmicutes | Lactobacillales_u_s | 1 | 4^.^19 | 0^.^0027 |
| Firmicutes | Fructobacillus fructosus | 4 | 4^.^06 | 0^.^016 |
| Firmicutes | Lactococcus lactis | 4 | 3^.^98 | 0^.^0014 |
| Firmicutes | Pediococcus acidilactici | 4 | 3^.^96 | 0^.^003 |
| Actinobacteria | Adlercreutzia caecimuris | 1 | 3^.^82 | 0^.^0019 |
| Actinobacteria | Enterorhabdus_u_s | 3 | 3^.^79 | 0^.^0075 |
| Actinobacteria | Adlercreutzia muris | 3 | 3^.^78 | 0^.^007 |
| Firmicutes | Ligilactobacillus animalis | 1 | 3^.^68 | 0^.^012 |
| Firmicutes | Firmicutes_u_s | 1 | 3^.^62 | 0^.^031 |
| Firmicutes | Roseburia_u_s | 1 | 3^.^56 | 0^.^013 |
| Actinobacteria | Adlercreutzia mucosicola | 3 | 3^.^53 | 0^.^004 |
| Actinobacteria | Bifidobacterium pseudolongum | 1 | 3^.^51 | 0^.^041 |
| Bacteroidetes | Parabacteroides distasonis | 1 | 3^.^47 | 0^.^034 |

**Supplemental Table 3: LEfSe table – predictive functional analysis, metabolic pathways**

| **Metabolic pathways** | **Enriched cohort** | **LDA score** | **P-value** |
| --- | --- | --- | --- |
| Aerobic respiration I (cytochrome c) | 4 | 5^.^51 | 0^.^0011 |
| Pyruvate fermentation to isobutanol | 2 | 4^.^22 | 0^.^015 |
| L-isoleucine biosynthesis I | 1 | 4^.^17 | 0^.^008 |
| tRNA charging | 3 | 4^.^16 | 0^.^0013 |
| UMP biosynthesis | 3 | 4^.^09 | 0^.^003 |
| Nicotine degradation IV | 2 | 4^.^09 | 0^.^007 |
| Superpathway of branched amino acid biosynthesis | 1 | 3^.^99 | 0^.^024 |
| Queuosine biosynthesis | 3 | 3^.^98 | 0^.^018 |
| L-isoleucine biosynthesis III | 1 | 3^.^97 | 0^.^024 |
| UDP-N-acetylmuramoyl-pentapeptide biosynthesis II | 3 | 3^.^97 | 0^.^0016 |
| 5-aminoimidazole ribonucleotide biosynthesis II | 3 | 3^.^97 | 0^.^029 |
| Superpathway of 5-aminoimidazole ribonucleotide biosynthesis | 3 | 3^.^95 | 0^.^029 |
| UDP-N-acetylmuramoyl-pentapeptide biosynthesis I | 3 | 3^.^95 | 0^.^0016 |
| 5-aminoimidazole ribonucleotide biosynthesis I | 3 | 3^.^94 | 0^.^0061 |
| Peptidoglycan biosynthesis I | 3 | 3^.^91 | 0^.^004 |
| L-lysine biosynthesis VI | 3 | 3^.^88 | 0^.^027 |
| Pentose phosphate pathway | 3 | 3^.^80 | 0^.^015 |
| Glycolysis I | 3 | 3^.^75 | 0^.^0046 |
| Calvin-Benson-Bassham cycle | 1 | 3^.^73 | 0^.^0043 |
| L-lysine biosynthesis II | 3 | 3^.^73 | 0^.^0075 |
| Glycolysis II | 3 | 3^.^73 | 0^.^0046 |
| Inosine-5’-phosphate biosynthesis III | 1 | 3^.^73 | 0^.^0056 |
| Methylerythritol phosphate pathway II | 1 | 3^.^69 | 0^.^0139 |
| Homolactic fermentation | 3 | 3^.^69 | 0^.^0026 |
| Superpathway of L-lysine, L-threonine and L-methionine biosynthesis II | 3 | 3^.^69 | 0^.^004 |
| Methylerythritol phosphate pathway I | 3 | 3^.^69 | 0^.^0047 |
| Cis-vaccenate biosynthesis | 3 | 3^.^65 | 0^.^0028 |
| Gondoate biosynthesis (anaerobic) | 3 | 3^.^64 | 0^.^0025 |
| Pyrimidine deoxyribonucleotides de novo biosynthesis II | 1 | 3^.^64 | 0^.^042 |
| Phosphopantothenate biosynthesis I | 1 | 3^.^63 | 0^.^007 |
| N10-formyl-tetrahydrofolate biosynthesis | 3 | 3^.^6 | 0^.^0076 |
| Superpathway of purine nucleotides de novo biosynthesis I | 1 | 3^.^6 | 0^.^024 |
| Superpathway of purine nucleotides de novo biosynthesis II | 1 | 3^.^58 | 0^.^0205 |
| Gluconeogenesis I | 1 | 3^.^57 | 0^.^017 |
| Isoprene biosynthesis I | 1 | 3^.^56 | 0^.^0027 |
| Stachyose degradation | 3 | 3^.^55 | 0^.^014 |
| Superpathway of L-threonine biosynthesis | 1 | 3^.^54 | 0^.^036 |
| Superpathway of guanosine nucleotides degradation (plants) | 3 | 3^.^53 | 0^.^009 |
| Sucrose degradation III (sucrose invertase) | 3 | 3^.^52 | 0^.^041 |
| Pantothenate and coenzyme A biosynthesis I | 3 | 3^.^51 | 0^.^0102 |
| Superpathway of L-isoleucine biosynthesis I | 1 | 3^.^51 | 0^.^0142 |
| Anaerobic energy metabolism (invertebrates, cytosol) | 3 | 3^.^5 | 0^.^041 |
| O-antigen building blocks biosynthesis (E. coli) | 3 | 3^.^49 | 0^.^043 |
| L-tryptophan biosynthesis | 1 | 3^.^48 | 0^.^041 |
| Pantothenate and coenzyme A biosynthesis III | 3 | 3^.^46 | 0^.^01 |
| C4 photosynthetic carbon assimilation cycle, NADP-ME type | 1 | 3^.^73 | 0^.^048 |
