# Supplementary material for: Early life microbiome disbalance impacts neuroendocrine outcomes in pre-pubertal mice in a sexually dimorphic manner
Source: Front Microbiol. 2025 Jun 20;16:1504513. doi: 10.3389/fmicb.2025.1504513 (PMC12277575; doi:10.3389/fmicb.2025.1504513)
Supplement: Supplementary file 1 [file Supplementary_file_1.zip › Supplementary Table 4.DOCX]

**Supplemental Table 4:** DEGs in adrenal medullae of male offspring: comparison male control vs. male Abx, Log2 FC=1.

| **Gene ID** | **Gene Name** | **Mean M Con** | **Mean M Abx** | **pvalue** | **padj** |
| --- | --- | --- | --- | --- | --- |
| ENSMUSG00000026818 | Cel | 0.634969799 | 0 | 3.4099E-09 | 2.7941E-05 |
| ENSMUSG00000089739 | Gm20431 | 0 | 67.30977658 | 1.9674E-06 | 0.006045384 |
| ENSMUSG00000108022 | Gm7298 | 0 | 52.82396861 | 1.0677E-17 | 2.62459E-13 |
| ENSMUSG00000030228 | Pik3c2g | 1.690009884 | 329.5575656 | 1.4257E-05 | 0.035046221 |
| ENSMUSG00000021214 | Akr1c18 | 27.77182845 | 17354.79193 | 6.5844E-08 | 0.000269761 |
| ENSMUSG00000057465 | Saa2 | 0 | 1.023275546 | 5.841E-08 | 0.000269761 |
| ENSMUSG00000039488 | Cntn5 | 0.634969799 | 0 | 2.0809E-12 | 2.55766E-08 |
| ENSMUSG00000020429 | Igfbp1 | 0 | 42.63648109 | 4.6489E-09 | 2.85697E-05 |
| ENSMUSG00000061947 | Serpina10 | 0 | 16.37240874 | 6.8526E-06 | 0.018716657 |
| ENSMUSG00000035896 | Rnase1 | 0 | 0.371999779 | 2.2055E-05 | 0.049287547 |
| ENSMUSG00000024868 | Dkk1 | 0 | 0.495999705 | 4.7873E-07 | 0.001681158 |
