# Supplementary material for: Early life microbiome disbalance impacts neuroendocrine outcomes in pre-pubertal mice in a sexually dimorphic manner
Source: Front Microbiol. 2025 Jun 20;16:1504513. doi: 10.3389/fmicb.2025.1504513 (PMC12277575; doi:10.3389/fmicb.2025.1504513)
Supplement: Supplementary file 1 [file Supplementary_file_1.zip › Supplementary Table 5.DOCX]

**Supplemental Table 5:** DEGs in adrenal medullae of female offspring: comparison female control vs. female Abx, Log2 FC=1. Included are protein coding transcripts.

| **Gene ID** | **GeneName** | **Mean F Con** | **Mean F Abx** | **pvalue** | **padj** |
| --- | --- | --- | --- | --- | --- |
