# Supplementary material for: Early life microbiome disbalance impacts neuroendocrine outcomes in pre-pubertal mice in a sexually dimorphic manner
Source: Front Microbiol. 2025 Jun 20;16:1504513. doi: 10.3389/fmicb.2025.1504513 (PMC12277575; doi:10.3389/fmicb.2025.1504513)
Supplement: Supplementary file 1 [file Supplementary_file_1.zip › Supplementary Table 6.DOCX]

**Supplemental Table 6: Sexually dimorphic DEG in adrenal medullae of control groups**

Female vs Male comparison, Log2 FC=2, mean values shown

| **GeneID** |  | **GeneName** | **F Con** | **M Con** | **log2FoldChange** | **padj** |
| --- | --- | --- | --- | --- | --- | --- |
| ENSMUSG00000099032 |  | Tcf24 | 1294.296651 | 264.9405865 | 2.290405413 | 0.000195061 |
| ENSMUSG00000025955 |  | Akr1cl | 71916.82111 | 29906.96759 | 1.265890999 | 0.004890244 |
| ENSMUSG00000062209 |  | Erbb4 | 195.2973052 | 26.76904661 | 2.865523081 | 0.000949438 |
| ENSMUSG00000101344 |  | Gm29183 | 40.08236206 | 8.329927371 | 2.373703139 | 0.00198323 |
| ENSMUSG00000047443 |  | Erfe | 36.43406277 | 265.2128233 | -2.842698229 | 2.33728E-05 |
| ENSMUSG00000101952 |  | Gm10550 | 21.02009619 | 4.109528127 | 2.448481686 | 0.04634152 |
| ENSMUSG00000045382 |  | Cxcr4 | 693.7804627 | 2116.410062 | -1.610196508 | 0.033829578 |
| ENSMUSG00000042510 |  | AA986860 | 216.9530685 | 701.1051284 | -1.700428441 | 8.83259E-08 |
| ENSMUSG00000026435 |  | Slc45a3 | 703.3049366 | 1651.354741 | -1.235115285 | 4.21458E-05 |
| ENSMUSG00000070644 |  | Etnk2 | 160.8563579 | 828.8814356 | -2.363339396 | 0.013969743 |
| ENSMUSG00000033849 |  | B3galt2 | 1169.130504 | 3337.776039 | -1.513895545 | 0.034082216 |
| ENSMUSG00000097754 |  | Ptgs2os2 | 127.5100693 | 22.01590092 | 2.541126746 | 0.003831315 |
| ENSMUSG00000033544 |  | Angptl1 | 244.632035 | 93.80813599 | 1.385874897 | 0.0003242 |
| ENSMUSG00000026601 |  | Axdnd1 | 76.04401891 | 212.8171399 | -1.479461687 | 0.019770769 |
| ENSMUSG00000026579 |  | F5 | 713.8195428 | 43.54410373 | 4.041102011 | 7.49694E-07 |
| ENSMUSG00000026675 |  | Hsd17b7 | 10431.84365 | 1814.925536 | 2.522866727 | 8.39991E-06 |
| ENSMUSG00000007107 |  | Atp1a4 | 205.0289933 | 35.42065963 | 2.537846765 | 0.003461651 |
| ENSMUSG00000026532 |  | Spta1 | 242.124496 | 37.0786002 | 2.709350399 | 0.015470751 |
| ENSMUSG00000038949 |  | Cnst | 2894.207494 | 1395.267141 | 1.051801007 | 0.006888308 |
| ENSMUSG00000039384 |  | Dusp10 | 293.5778363 | 717.3143049 | -1.288647789 | 0.015544409 |
| ENSMUSG00000026630 |  | Batf3 | 27.94827533 | 86.17263628 | -1.621373048 | 0.002266107 |
| ENSMUSG00000026623 |  | Lpgat1 | 9025.40003 | 4238.461335 | 1.090688186 | 0.001650958 |
| ENSMUSG00000016262 |  | Sertad4 | 796.1463898 | 319.2231682 | 1.309801855 | 0.000157268 |
| ENSMUSG00000026650 |  | Meig1 | 23.33831793 | 4.395059475 | 2.284366615 | 0.027095209 |
| ENSMUSG00000026643 |  | Nmt2 | 5514.177313 | 2744.208698 | 1.007317876 | 1.82022E-06 |
| ENSMUSG00000026672 |  | Optn | 2096.869405 | 6095.730245 | -1.539634851 | 1.31198E-10 |
| ENSMUSG00000045319 |  | Proser2 | 296.4584576 | 107.6352626 | 1.447337099 | 0.000841617 |
| ENSMUSG00000062061 |  | Obp2a | 32.63214562 | 0.286373416 | 6.365371131 | 0.002342864 |
| ENSMUSG00000026939 |  | Tmem141 | 539.0654312 | 222.9540227 | 1.272676218 | 1.48196E-06 |
| ENSMUSG00000001864 |  | Aif1l | 300.3497563 | 784.6045969 | -1.384073971 | 0.001726475 |
| ENSMUSG00000050592 |  | Fam78a | 176.6513436 | 69.09102419 | 1.363371368 | 0.004462937 |
| ENSMUSG00000026885 |  | Ttll11 | 215.1517932 | 53.80199778 | 2.009841475 | 0.005447726 |
| ENSMUSG00000050447 |  | Lypd6 | 1095.411168 | 120.5218982 | 3.186971966 | 1.75225E-08 |
| ENSMUSG00000053475 |  | Tnfaip6 | 79.36243569 | 257.2629732 | -1.669547541 | 0.034901516 |
| ENSMUSG00000026950 |  | Neb | 910.5489157 | 102.7076558 | 3.149508269 | 0.020108765 |
| ENSMUSG00000026836 |  | Acvr1 | 2805.063192 | 6805.406216 | -1.278165591 | 6.29264E-05 |
| ENSMUSG00000027016 |  | Zfp385b | 33.66142638 | 7.440057679 | 2.155808692 | 0.00241836 |
| ENSMUSG00000027004 |  | Frzb | 22477.90737 | 1928.490486 | 3.543575778 | 1.41708E-06 |
| ENSMUSG00000045392 |  | Olfr1033 | 502.2535309 | 202.4290116 | 1.305836326 | 0.02714088 |
| ENSMUSG00000027230 |  | Creb3l1 | 661.9212822 | 1478.361531 | -1.161631494 | 2.72971E-15 |
| ENSMUSG00000040434 |  | Large2 | 21.22933487 | 4.247548882 | 2.31941678 | 0.003043861 |
| ENSMUSG00000012350 |  | Ehf | 114.8836551 | 8.42446593 | 3.797007535 | 0.001386019 |
| ENSMUSG00000027171 |  | Prrg4 | 539.6806927 | 1940.797753 | -1.843961278 | 3.78738E-06 |
| ENSMUSG00000005973 |  | Rcn1 | 9102.471611 | 3242.19618 | 1.489048565 | 9.60685E-12 |
| ENSMUSG00000048482 |  | Bdnf | 40.66974579 | 1.92365017 | 4.177915494 | 2.70846E-06 |
| ENSMUSG00000027313 |  | Chac1 | 343.1444745 | 101.132981 | 1.775331184 | 0.000624585 |
| ENSMUSG00000070719 |  | Pla2g4d | 41.31711922 | 0.859120247 | 5.171144598 | 1.53617E-06 |
| ENSMUSG00000054484 |  | Tmem62 | 1422.682359 | 670.9770946 | 1.085956148 | 0.014188116 |
| ENSMUSG00000033498 |  | Strc | 3.292025003 | 28.43131106 | -3.018528935 | 0.000387755 |
| ENSMUSG00000027360 |  | Hdc | 1762.169912 | 135.0207565 | 3.710484272 | 6.59172E-07 |
| ENSMUSG00000037902 |  | Sirpa | 7439.175055 | 3027.785547 | 1.297458517 | 0.000512178 |
| ENSMUSG00000027401 |  | Tgm3 | 100.7551592 | 7.495191655 | 3.714912951 | 0.008545766 |
| ENSMUSG00000027442 |  | Cst8 | 2575.898858 | 57.54695386 | 5.487891957 | 5.55062E-08 |
| ENSMUSG00000068115 |  | Ninl | 1730.335523 | 761.0674404 | 1.187040513 | 7.30295E-06 |
| ENSMUSG00000032715 |  | Trib3 | 327.2866282 | 42.23788447 | 2.945317206 | 0.000705947 |
| ENSMUSG00000027602 |  | Map1lc3a | 8439.40871 | 4189.132291 | 1.010804954 | 4.7488E-09 |
| ENSMUSG00000027611 |  | Procr | 1281.330208 | 2652.557787 | -1.047982921 | 0.026014349 |
| ENSMUSG00000038180 |  | Spag4 | 34.21324527 | 118.2355032 | -1.726811289 | 8.97317E-06 |
| ENSMUSG00000027412 |  | Lpin3 | 2310.48287 | 959.1646833 | 1.269455897 | 5.95752E-07 |
| ENSMUSG00000074607 |  | Tox2 | 160.4804488 | 453.9284433 | -1.509695474 | 0.000592465 |
| ENSMUSG00000042845 |  | Wfdc12 | 39.98664657 | 0 | 8.277227858 | 0.000395669 |
| ENSMUSG00000070533 |  | Wfdc8 | 6.850615876 | 50.30847452 | -2.913500984 | 0.029274606 |
| ENSMUSG00000053166 |  | Cdh22 | 75.07946918 | 234.2460557 | -1.652742651 | 0.004228546 |
| ENSMUSG00000056501 |  | Cebpb | 3364.782004 | 1561.420027 | 1.107351604 | 1.28042E-06 |
| ENSMUSG00000008999 |  | Bmp7 | 1618.635144 | 4855.736838 | -1.586202229 | 7.00384E-05 |
| ENSMUSG00000069094 |  | Pde7a | 4378.272935 | 1806.877462 | 1.277618489 | 5.90016E-06 |
| ENSMUSG00000001865 |  | Cpa3 | 248.4583999 | 84.25439097 | 1.562862193 | 0.03254835 |
| ENSMUSG00000011463 |  | Cpb1 | 35771.01331 | 344.3509576 | 6.698171933 | 4.97159E-05 |
| ENSMUSG00000058174 |  | Gm5148 | 3.418855986 | 30.35880731 | -2.978100171 | 0.022560868 |
| ENSMUSG00000046743 |  | Fat4 | 3405.860177 | 1408.475874 | 1.274575555 | 0.001078441 |
| ENSMUSG00000090919 |  | Pabpc4l | 187.2585861 | 62.11763655 | 1.568484461 | 0.000318076 |
| ENSMUSG00000103527 |  | Gm37261 | 88.2849435 | 21.76135792 | 2.050465634 | 0.032184249 |
| ENSMUSG00000049404 |  | Rarres1 | 4.85588331 | 59.46113922 | -3.423645657 | 0.0143524 |
| ENSMUSG00000027955 |  | Fam198b | 2043.954936 | 4182.507561 | -1.034381696 | 0.006644839 |
| ENSMUSG00000041842 |  | Fhdc1 | 866.6652706 | 119.6624432 | 2.85615992 | 5.904E-08 |
| ENSMUSG00000041750 |  | Cd1d2 | 27.92807069 | 1.487843122 | 4.102082869 | 0.003995113 |
| ENSMUSG00000028076 |  | Cd1d1 | 17701.11099 | 1645.799094 | 3.426753174 | 1.18169E-08 |
| ENSMUSG00000059743 |  | Fdps | 15596.18168 | 4115.423962 | 1.922038469 | 0.002342864 |
| ENSMUSG00000042784 |  | Muc1 | 99.39047149 | 9.216197256 | 3.429684188 | 0.019055444 |
| ENSMUSG00000052415 |  | Tchh | 291.2641847 | 37.58888516 | 2.961555264 | 2.76826E-05 |
| ENSMUSG00000044505 |  | Lingo4 | 18.83535053 | 45.21561174 | -1.238086281 | 0.023086196 |
| ENSMUSG00000033386 |  | Frrs1 | 1724.00336 | 602.5942351 | 1.517358691 | 0.000202254 |
| ENSMUSG00000074207 |  | Adh1 | 185417.8475 | 70750.41671 | 1.389979974 | 0.031275334 |
| ENSMUSG00000028273 |  | Pdlim5 | 9313.435812 | 3689.516523 | 1.336130772 | 4.28621E-05 |
| ENSMUSG00000056025 |  | Clca3a1 | 1099.511828 | 395.2879916 | 1.477468457 | 0.028143219 |
| ENSMUSG00000028179 |  | Cth | 1492.804913 | 329.129338 | 2.180818318 | 1.00255E-06 |
| ENSMUSG00000041261 |  | Car8 | 4121.959433 | 191.6408222 | 4.428172306 | 6.21358E-07 |
|  |  |  |  |  |  |  |
| ENSMUSG00000073988 |  | Ttpa | 529.4443658 | 193.9348431 | 1.454109146 | 0.001140584 |
| ENSMUSG00000055761 |  | Nkain3 | 133.6230472 | 4.121179907 | 4.910937176 | 1.9747E-06 |
| ENSMUSG00000054659 |  | Pm20d2 | 538.5343151 | 139.5309107 | 1.941903943 | 2.36298E-09 |
| ENSMUSG00000044813 |  | Shb | 2556.527371 | 1260.169469 | 1.022652814 | 7.43426E-05 |
| ENSMUSG00000028339 |  | Col15a1 | 3766.56386 | 7682.282747 | -1.028398963 | 0.027901324 |
| ENSMUSG00000086712 |  | AI427809 | 141.9689197 | 38.76644814 | 1.879615665 | 0.001451306 |
| ENSMUSG00000015243 |  | Abca1 | 31338.26678 | 8548.248134 | 1.874322045 | 2.94519E-05 |
| ENSMUSG00000028420 |  | Tmem38b | 1232.448633 | 584.4960362 | 1.075230598 | 0.004890576 |
| ENSMUSG00000089945 |  | Pakap | 3630.340195 | 1279.273648 | 1.505734089 | 0.01058091 |
| ENSMUSG00000028378 |  | Ptgr1 | 831.9914973 | 1819.438448 | -1.12926889 | 0.006905283 |
| ENSMUSG00000063851 |  | Rnf183 | 1.706121163 | 22.30119336 | -3.302942288 | 0.022558851 |
| ENSMUSG00000028392 |  | Bspry | 139.8989997 | 375.9479423 | -1.411575188 | 5.06761E-05 |
| ENSMUSG00000043753 |  | Dmrta1 | 134.687753 | 9.063759614 | 3.886908108 | 6.40839E-05 |
| ENSMUSG00000052684 |  | Jun | 6225.474627 | 13536.21124 | -1.119981678 | 3.47165E-05 |
| ENSMUSG00000034926 |  | Dhcr24 | 17027.54903 | 5385.962161 | 1.660615729 | 0.021909593 |
| ENSMUSG00000085873 |  | Ttc39aos1 | 94.96217222 | 198.8196299 | -1.082722227 | 0.035479366 |
| ENSMUSG00000070803 |  | Cited4 | 380.761913 | 84.62448586 | 2.167483487 | 0.017859547 |
| ENSMUSG00000028885 |  | Smpdl3b | 1116.992788 | 34.24874778 | 5.021884621 | 7.35517E-08 |
| ENSMUSG00000070691 |  | Runx3 | 118.0087721 | 241.2868631 | -1.006516532 | 0.001601124 |
| ENSMUSG00000086845 |  | Gm13010 | 123.2277058 | 18.40284403 | 2.776325822 | 0.008622694 |
| ENSMUSG00000028737 |  | Aldh4a1 | 7169.352485 | 2768.110042 | 1.372950746 | 0.002124189 |
| ENSMUSG00000028927 |  | Padi2 | 744.2715193 | 100.8344662 | 2.885757339 | 0.000147795 |
| ENSMUSG00000058579 |  | Cela2a | 38416.72825 | 1.621539993 | 14.57668039 | 0.000114146 |
| ENSMUSG00000028602 |  | Tnfrsf8 | 33.48283995 | 11.59499104 | 1.586810363 | 0.015392946 |
| ENSMUSG00000058183 |  | Mmel1 | 454.2806255 | 102.2391881 | 2.159369162 | 0.01292316 |
| ENSMUSG00000029059 |  | Fam213b | 2629.587967 | 892.8595413 | 1.559008547 | 0.005587388 |
| ENSMUSG00000029092 |  | D5Ertd615e | 8.099762987 | 25.14078189 | -1.584059244 | 0.017405119 |
| ENSMUSG00000029212 |  | Gabrb1 | 113.833995 | 16.62033954 | 2.760998179 | 0.019605527 |
| ENSMUSG00000035505 |  | Cox18 | 955.0812953 | 407.5102838 | 1.228999869 | 1.41708E-06 |
| ENSMUSG00000029384 |  | 2010109A12Rik | 22.53324097 | 9.294156986 | 1.244282436 | 0.036174657 |
| ENSMUSG00000029304 |  | Spp1 | 4969.291421 | 202.40613 | 4.618278672 | 0.011736231 |
| ENSMUSG00000034438 |  | Gbp8 | 37.05754976 | 75.4969445 | -1.041859137 | 0.040436259 |
| ENSMUSG00000029544 |  | Cabp1 | 665.0043849 | 209.8482522 | 1.6635537 | 0.043489999 |
| ENSMUSG00000029359 |  | Tesc | 1979.659093 | 4100.925424 | -1.051113495 | 0.00947959 |
| ENSMUSG00000086847 |  | Tbx3os2 | 29.20226687 | 72.72126308 | -1.348473645 | 0.035620653 |
| ENSMUSG00000032754 |  | Slc8b1 | 982.5428077 | 428.5063389 | 1.194659043 | 1.75434E-06 |
| ENSMUSG00000046245 |  | Pilra | 45.63152814 | 95.09392297 | -1.011985892 | 0.010665236 |
| ENSMUSG00000048988 |  | Elfn1 | 1322.070675 | 46.65828561 | 4.851325748 | 3.69231E-13 |
| ENSMUSG00000036565 |  | Ttyh3 | 4527.366254 | 2171.922381 | 1.060616402 | 0.002549823 |
| ENSMUSG00000029759 |  | Pon3 | 8909.870293 | 4113.558828 | 1.115294359 | 0.003998964 |
| ENSMUSG00000029752 |  | Asns | 2854.933523 | 1117.200408 | 1.352756394 | 6.40839E-05 |
| ENSMUSG00000042460 |  | C1galt1 | 5913.595472 | 2604.933165 | 1.182956926 | 0.005926808 |
| ENSMUSG00000046178 |  | Nxph1 | 238.5238612 | 45.14605351 | 2.404048054 | 0.004919939 |
| ENSMUSG00000029552 |  | Tes | 1379.51877 | 550.5664715 | 1.327487387 | 0.000517239 |
| ENSMUSG00000029695 |  | Aass | 682.146693 | 40.23939657 | 4.0949043 | 1.43109E-09 |
| ENSMUSG00000017978 |  | Cadps2 | 799.1744662 | 324.0009847 | 1.305116622 | 0.000368444 |
| ENSMUSG00000029772 |  | Ahcyl2 | 12817.97421 | 6027.098439 | 1.088701482 | 0.000496138 |
| ENSMUSG00000071553 |  | Cpa2 | 6687.137571 | 20.50523204 | 8.352376416 | 9.2207E-10 |
| ENSMUSG00000084965 |  | Gm13857 | 276.8112643 | 704.9877854 | -1.349324355 | 0.047058565 |
| ENSMUSG00000038641 |  | Akr1d1 | 68979.17923 | 189.2646654 | 8.512744441 | 5.97419E-10 |
| ENSMUSG00000106889 |  | Gm7463 | 1406.836942 | 4.508695699 | 8.397368333 | 3.79704E-08 |
| ENSMUSG00000068587 |  | Mgam | 185.7092036 | 5.449018589 | 5.097912569 | 2.9085E-09 |
| ENSMUSG00000102802 |  | Mgam2-ps | 162.8165641 | 0 | 10.30155242 | 0.000213163 |
| ENSMUSG00000029864 |  | Gstk1 | 5166.526746 | 1906.873918 | 1.43860639 | 0.000795636 |
| ENSMUSG00000071506 |  | Tmem139 | 21.54140619 | 0.286373416 | 5.769728942 | 0.004171789 |
| ENSMUSG00000029814 |  | Igf2bp3 | 146.274825 | 30.82668368 | 2.241682591 | 0.001141516 |
| ENSMUSG00000029822 |  | Osbpl3 | 2622.709774 | 5249.856598 | -1.00090469 | 0.000337927 |
| ENSMUSG00000038058 |  | Nod1 | 762.9732471 | 331.6301622 | 1.202250936 | 0.002173464 |
| ENSMUSG00000036390 |  | Gadd45a | 645.3332543 | 266.2996952 | 1.275284602 | 0.013685638 |
| ENSMUSG00000056091 |  | St3gal5 | 6361.899678 | 13901.36497 | -1.127776875 | 0.000305492 |
| ENSMUSG00000057278 |  | Snrpg | 4435.982587 | 2003.955431 | 1.147440581 | 0.000128905 |
| ENSMUSG00000033152 |  | Podxl2 | 757.1530832 | 1709.053447 | -1.176896885 | 2.94646E-17 |
| ENSMUSG00000030089 |  | Slc41a3 | 1255.546877 | 3607.751405 | -1.52315277 | 1.64304E-06 |
|  |  |  |  |  |  |  |
| ENSMUSG00000035158 |  | Mitf | 877.9847978 | 408.7973051 | 1.097389219 | 5.58669E-08 |
| ENSMUSG00000030077 |  | Chl1 | 8012.47409 | 1039.234493 | 2.947005158 | 5.50256E-05 |
| ENSMUSG00000064293 |  | Cntn4 | 58.27654893 | 25.61406311 | 1.214441107 | 0.011736231 |
| ENSMUSG00000034648 |  | Lrrn1 | 8828.173037 | 1601.338937 | 2.462967449 | 1.87445E-05 |
| ENSMUSG00000034387 |  | Ssu2 | 658.621327 | 13.22265997 | 5.615630278 | 0.000298563 |
| ENSMUSG00000049112 |  | Oxtr | 22860.57593 | 1844.811965 | 3.631680695 | 1.54416E-05 |
| ENSMUSG00000030257 |  | Srgap3 | 1171.525888 | 2413.938176 | -1.042020246 | 0.005932626 |
| ENSMUSG00000025701 |  | Alox5 | 47.33432236 | 17.95034016 | 1.366825513 | 0.006759959 |
| ENSMUSG00000030111 |  | A2m | 5668.544114 | 348.979994 | 4.024538215 | 1.90128E-06 |
| ENSMUSG00000043832 |  | Clec4a3 | 82.19679076 | 172.0303087 | -1.054570722 | 0.039140496 |
| ENSMUSG00000030351 |  | Tspan11 | 372.2809335 | 1706.916612 | -2.195272392 | 0.030766906 |
| ENSMUSG00000079293 |  | Clec7a | 113.0753167 | 284.9745002 | -1.332251463 | 0.00950874 |
| ENSMUSG00000090698 |  | Apold1 | 593.8466066 | 1979.106991 | -1.733382819 | 0.001015949 |
| ENSMUSG00000030219 |  | Erp27 | 233.9536051 | 2.276331535 | 6.675219712 | 0.031471528 |
| ENSMUSG00000044378 |  | Slc15a5 | 22.49937368 | 0 | 7.456630898 | 0.001930002 |
| ENSMUSG00000008540 |  | Mgst1 | 50635.90852 | 13567.18663 | 1.900086931 | 5.67647E-05 |
| ENSMUSG00000030228 |  | Pik3c2g | 12717.94198 | 1.690009884 | 12.99849831 | 1.31198E-10 |
| ENSMUSG00000107956 |  | Speer9-ps1 | 54.3720597 | 5.883744665 | 3.226985718 | 1.74568E-07 |
| ENSMUSG00000040714 |  | Klc3 | 23.44277996 | 91.43760805 | -1.940011472 | 0.003509613 |
| ENSMUSG00000097215 |  | Gm26550 | 15.94478185 | 1.002306955 | 3.651146366 | 0.006106767 |
| ENSMUSG00000057454 |  | Lypd3 | 49.49629517 | 2.965115436 | 4.215710186 | 4.02339E-05 |
| ENSMUSG00000074272 |  | Ceacam1 | 1554.243864 | 428.397031 | 1.859729224 | 0.002569575 |
| ENSMUSG00000052974 |  | Cyp2f2 | 13030.33723 | 120.5631152 | 6.756066524 | 9.9192E-12 |
| ENSMUSG00000084174 |  | Sycn | 1274.536916 | 0 | 31.27633178 | 9.99712E-06 |
| ENSMUSG00000005553 |  | Atp4a | 36.7447197 | 130.2604142 | -1.884464367 | 2.40342E-06 |
| ENSMUSG00000050440 |  | Hamp | 597.3412822 | 1.266696685 | 9.485364661 | 8.00951E-14 |
| ENSMUSG00000056836 |  | Gm6851 | 40.44662416 | 17.71754194 | 1.238345431 | 0.015979711 |
| ENSMUSG00000063903 |  | Klk1 | 3431.353727 | 0.286373416 | 13.21023263 | 0.02553128 |
| ENSMUSG00000003271 |  | Sult2b1 | 34.94335861 | 96.87834313 | -1.48032309 | 0.000169671 |
| ENSMUSG00000040212 |  | Emp3 | 2006.136694 | 4508.864515 | -1.1686149 | 1.50759E-06 |
| ENSMUSG00000040189 |  | Ccdc114 | 354.7745766 | 1010.109079 | -1.511663784 | 0.000118434 |
| ENSMUSG00000055026 |  | Gabrg3 | 850.0601292 | 391.3762061 | 1.120353629 | 0.022592355 |
| ENSMUSG00000030549 |  | Rhcg | 20.73307394 | 221.1305299 | -3.465288911 | 1.23679E-06 |
| ENSMUSG00000030607 |  | Acan | 67.3701991 | 861.8987648 | -3.675858434 | 0.001503294 |
| ENSMUSG00000087088 |  | Gm16638 | 263.5676814 | 84.75253897 | 1.638794012 | 0.002712561 |
| ENSMUSG00000046027 |  | Stard5 | 2045.060989 | 627.2846677 | 1.705247189 | 0.00320539 |
| ENSMUSG00000108825 |  | Gm45838 | 140.5167123 | 39.62424851 | 1.844232695 | 0.0040349 |
| ENSMUSG00000030562 |  | Nox4 | 686.2577269 | 102.0992546 | 2.751466521 | 1.70221E-05 |
| ENSMUSG00000074006 |  | Omp | 228.7847255 | 9.029282081 | 4.685844975 | 0.000466544 |
| ENSMUSG00000035211 |  | Xrra1 | 136.081955 | 23.72632834 | 2.539171033 | 0.012913478 |
| ENSMUSG00000109002 |  | Gm38405 | 17.01566594 | 4.610801056 | 1.787448313 | 0.025076185 |
| ENSMUSG00000030688 |  | Stard10 | 597.103965 | 1435.650478 | -1.266727676 | 0.000944955 |
| ENSMUSG00000001827 |  | Folr1 | 81.88096598 | 8.001152731 | 3.413004755 | 0.000187884 |
| ENSMUSG00000038244 |  | Mical2 | 874.7782803 | 1876.742914 | -1.101807402 | 0.002782156 |
| ENSMUSG00000030905 |  | Crym | 24.75073151 | 279.5672604 | -3.499920687 | 3.52769E-11 |
| ENSMUSG00000042978 |  | Sbk1 | 3726.603553 | 922.2314819 | 2.014641358 | 1.28554E-11 |
| ENSMUSG00000049350 |  | Zg16 | 7464.146241 | 0 | 39.46587142 | 5.75129E-09 |
| ENSMUSG00000030789 |  | Itgax | 103.4580401 | 302.2479795 | -1.541104266 | 0.023086196 |
| ENSMUSG00000047517 |  | Dmbt1 | 6832.891134 | 0 | 38.82896951 | 1.06961E-08 |
| ENSMUSG00000040205 |  | Cuzd1 | 1534.7214 | 0.844464457 | 10.91970843 | 0.032258751 |
| ENSMUSG00000030956 |  | Fam53b | 5212.561561 | 2334.432503 | 1.158637794 | 1.82022E-06 |
| ENSMUSG00000030861 |  | Acadsb | 22762.68771 | 8388.12067 | 1.440259889 | 4.84016E-06 |
| ENSMUSG00000025464 |  | Paox | 1091.429557 | 518.8563479 | 1.070362472 | 0.00131947 |
| ENSMUSG00000060314 |  | Zfp941 | 240.2233599 | 511.9845599 | -1.097682199 | 0.018091716 |
| ENSMUSG00000025496 |  | Drd4 | 7.688042637 | 163.4417887 | -4.616524257 | 6.11114E-14 |
| ENSMUSG00000037887 |  | Dusp8 | 611.0184498 | 1401.119714 | -1.19363442 | 0.0238643 |
| ENSMUSG00000009545 |  | Kcnq1 | 282.6292602 | 842.1164879 | -1.575302929 | 0.003419793 |
| ENSMUSG00000037664 |  | Cdkn1c | 14232.53005 | 2862.751538 | 2.313655612 | 1.01951E-07 |
| ENSMUSG00000108934 |  | Gm44732 | 61.57789915 | 11.53493002 | 2.432892866 | 0.000279714 |
| ENSMUSG00000109061 |  | Map2k7 | 0 | 83.17974898 | -38.01919168 | 3.52769E-11 |
| ENSMUSG00000031453 |  | Rasa3 | 4294.481093 | 1701.949374 | 1.335501717 | 0.000669818 |
| ENSMUSG00000037738 |  | Nek5 | 396.9803029 | 1274.160478 | -1.685714036 | 2.77139E-07 |
| ENSMUSG00000109419 |  | Gm45163 | 29.92277183 | 2.535555312 | 3.762480791 | 0.009040616 |
| ENSMUSG00000031488 |  | Rab11fip1 | 405.2440473 | 1161.698317 | -1.520067285 | 0.005270677 |
| ENSMUSG00000055976 |  | Cldn23 | 11.76013578 | 0.996060031 | 3.394099025 | 0.03804844 |
| ENSMUSG00000039633 |  | Lonrf1 | 580.6922022 | 1210.367902 | -1.054691995 | 0.001478577 |
| ENSMUSG00000031561 |  | Tenm3 | 3431.94346 | 1622.292728 | 1.081460074 | 0.008231511 |
| ENSMUSG00000031520 |  | Vegfc | 793.7194332 | 211.9551263 | 1.90353637 | 4.9842E-07 |
| ENSMUSG00000053886 |  | Sh2d4a | 580.4231309 | 86.95295473 | 2.730534847 | 2.9793E-10 |
| ENSMUSG00000030465 |  | Psd3 | 10692.14608 | 3865.157409 | 1.468061082 | 0.011736231 |
| ENSMUSG00000005413 |  | Hmox1 | 965.7144277 | 6916.525255 | -2.839992619 | 2.08144E-31 |
| ENSMUSG00000037148 |  | Arhgap10 | 2122.651458 | 997.6568226 | 1.088751606 | 1.75225E-08 |
| ENSMUSG00000074219 |  | Gm10644 | 80.60831871 | 40.19423024 | 1.013737504 | 0.02489782 |
| ENSMUSG00000031700 |  | Gpt2 | 12167.45928 | 2188.9944 | 2.474734585 | 0.002056602 |
| ENSMUSG00000031654 |  | Cbln1 | 91.60599135 | 3.820150701 | 4.57104108 | 0.001740711 |
| ENSMUSG00000031659 |  | Adcy7 | 701.6123489 | 1853.945459 | -1.400683888 | 2.19312E-11 |
| ENSMUSG00000033192 |  | Lpcat2 | 122.8186744 | 283.5682342 | -1.194139985 | 0.004171789 |
| ENSMUSG00000031872 |  | Bean1 | 543.1364069 | 1198.639139 | -1.144575458 | 0.04367965 |
| ENSMUSG00000031880 |  | Rrad | 48.89272083 | 130.5503918 | -1.421889404 | 0.016917653 |
| ENSMUSG00000031896 |  | Ctrl | 12904.26093 | 15.81322834 | 9.679663617 | 2.39465E-05 |
| ENSMUSG00000000303 |  | Cdh1 | 60.66438637 | 9.369954773 | 2.662054001 | 0.042104148 |
| ENSMUSG00000059854 |  | Hydin | 48.16412497 | 19.88059415 | 1.297611553 | 0.020657102 |
| ENSMUSG00000031750 |  | Il34 | 877.613356 | 374.6447491 | 1.232647371 | 0.01213578 |
| ENSMUSG00000033579 |  | Fa2h | 677.6400212 | 0 | 12.35838485 | 1.07905E-11 |
| ENSMUSG00000012519 |  | Mlkl | 1067.138403 | 508.4141668 | 1.071465929 | 0.000486878 |
| ENSMUSG00000031957 |  | Ctrb1 | 37211.90421 | 0 | 38.3115008 | 1.76197E-08 |
| ENSMUSG00000033998 |  | Kcnk1 | 134.391437 | 599.7198164 | -2.158769273 | 0.004971753 |
| ENSMUSG00000057060 |  | Slc35f3 | 81.32221001 | 14.32346218 | 2.551766566 | 0.016576346 |
| ENSMUSG00000070323 |  | Mmp27 | 49.86829333 | 5.301507996 | 3.303964802 | 0.031157855 |
| ENSMUSG00000049723 |  | Mmp12 | 75.47120521 | 218.494459 | -1.510037096 | 0.037045711 |
| ENSMUSG00000031936 |  | Hephl1 | 730.329897 | 68.12701582 | 3.430515475 | 0.002307326 |
| ENSMUSG00000004098 |  | Col5a3 | 14575.76263 | 4675.945113 | 1.640336704 | 0.010235625 |
| ENSMUSG00000111497 |  | Raver1 | 539.1182424 | 1112.214757 | -1.047671154 | 0.010617767 |
| ENSMUSG00000040146 |  | Rgl3 | 1194.240535 | 565.5447031 | 1.079205789 | 0.000813293 |
| ENSMUSG00000043067 |  | Dpy19l1 | 5923.311559 | 1970.572379 | 1.587625509 | 3.21363E-08 |
| ENSMUSG00000031995 |  | St14 | 87.30597238 | 18.69967026 | 2.169128402 | 0.000407529 |
| ENSMUSG00000011118 |  | Panx3 | 54.10838546 | 0 | 8.717126968 | 4.54889E-05 |
| ENSMUSG00000066749 |  | Olfr877 | 163.483747 | 0.4233132 | 8.459088114 | 0.000552385 |
| ENSMUSG00000032238 |  | Rora | 14867.75233 | 5255.377843 | 1.500448074 | 8.57731E-10 |
| ENSMUSG00000032360 |  | Hcrtr2 | 40.34913944 | 2.984937179 | 3.750253201 | 6.80702E-05 |
| ENSMUSG00000057933 |  | Gsta2 | 17.11412091 | 0 | 7.057362108 | 0.017690118 |
| ENSMUSG00000032417 |  | Rwdd2a | 287.4630263 | 92.08353058 | 1.635717875 | 0.000209943 |
| ENSMUSG00000033491 |  | Prss35 | 21742.44417 | 50910.43276 | -1.227550903 | 0.002359633 |
| ENSMUSG00000097195 |  | Snhg5 | 1450.503602 | 724.865753 | 1.00293036 | 0.042136194 |
| ENSMUSG00000049493 |  | Pls1 | 57.41974795 | 16.18921327 | 1.826029325 | 0.025331419 |
| ENSMUSG00000032531 |  | Amotl2 | 12347.0524 | 5528.490519 | 1.159154212 | 0.000191175 |
| ENSMUSG00000032561 |  | Acpp | 4916.463428 | 26.77710596 | 7.514810563 | 5.72607E-05 |
| ENSMUSG00000032578 |  | Cish | 783.1409518 | 300.9762113 | 1.387052362 | 0.015701784 |
| ENSMUSG00000025648 |  | Pfkfb4 | 391.3468165 | 853.5634598 | -1.125627458 | 0.039611272 |
| ENSMUSG00000033392 |  | Clasp2 | 9450.502505 | 20415.76606 | -1.111031312 | 7.46981E-05 |
| ENSMUSG00000056880 |  | Gadl1 | 52.07299188 | 4.270613539 | 3.486487982 | 0.001233614 |
| ENSMUSG00000040653 |  | Ppp1r14c | 614.4240781 | 172.4988768 | 1.836688973 | 1.30081E-07 |
| ENSMUSG00000060487 |  | Samd5 | 2075.947302 | 867.7567982 | 1.258938559 | 9.84937E-06 |
| ENSMUSG00000101969 |  | Gm20125 | 25.69149791 | 1.207716631 | 4.188278527 | 9.30155E-05 |
| ENSMUSG00000019850 |  | Tnfaip3 | 322.840483 | 651.8206363 | -1.007111439 | 0.007035307 |
| ENSMUSG00000053219 |  | Raet1e | 54.03165261 | 18.18325637 | 1.634126338 | 0.002972532 |
| ENSMUSG00000020010 |  | Vnn3 | 4077.604844 | 753.6659439 | 2.43669026 | 9.7479E-05 |
| ENSMUSG00000037440 |  | Vnn1 | 2332.302919 | 514.2173738 | 2.182662572 | 2.36726E-05 |
| ENSMUSG00000019889 |  | Ptprk | 2457.273277 | 1152.089604 | 1.093262248 | 0.000112945 |
| ENSMUSG00000039552 |  | Rsph4a | 102.2948479 | 11.80880958 | 3.139149161 | 0.004990694 |
| ENSMUSG00000071335 |  | Mfsd4b3 | 190.3824688 | 40.41261894 | 2.213891452 | 2.19706E-09 |
| ENSMUSG00000003746 |  | Man1a | 14510.99776 | 2544.784541 | 2.511695716 | 2.47801E-08 |
| ENSMUSG00000050953 |  | Gja1 | 14771.1329 | 3530.349742 | 2.064986169 | 9.74956E-09 |
| ENSMUSG00000020262 |  | Adarb1 | 2058.06812 | 5411.301015 | -1.394501766 | 0.001623604 |
| ENSMUSG00000020325 |  | Fstl3 | 366.4586175 | 1181.963236 | -1.69654616 | 4.37083E-10 |
| ENSMUSG00000015312 |  | Gadd45b | 618.6110004 | 1570.116243 | -1.34593212 | 5.58321E-06 |
| ENSMUSG00000020263 |  | Appl2 | 25521.99449 | 5858.403645 | 2.123350961 | 0.000282795 |
| ENSMUSG00000050108 |  | Bpifc | 127.0977702 | 4.016070539 | 5.02580361 | 0.016256271 |
| ENSMUSG00000090610 |  | Gm3571 | 84.6925482 | 23.41569131 | 1.842036589 | 0.013688473 |
| ENSMUSG00000020027 |  | Socs2 | 2205.875195 | 1058.418358 | 1.063542068 | 1.75434E-06 |
| ENSMUSG00000036499 |  | Eea1 | 8156.535527 | 4061.456591 | 1.006487258 | 0.000305492 |
| ENSMUSG00000054934 |  | Kcnmb4 | 31.16425455 | 135.0992265 | -2.122683181 | 0.000952217 |
| ENSMUSG00000052302 |  | Tbc1d30 | 236.8657018 | 102.8157839 | 1.216018111 | 0.004335858 |
| ENSMUSG00000025401 |  | Myo1a | 75.55368457 | 536.6971576 | -2.8364102 | 4.03747E-11 |
| ENSMUSG00000040127 |  | Sdr9c7 | 46.79569935 | 7.837787387 | 2.507198524 | 0.038444594 |
| ENSMUSG00000047631 |  | Apof | 390.3935152 | 54.30147303 | 2.863382082 | 6.90485E-07 |
| ENSMUSG00000009073 |  | Nf2 | 14083.4011 | 4963.308002 | 1.504702796 | 1.46548E-05 |
| ENSMUSG00000048834 |  | Vstm2a | 69.44944082 | 11.45937113 | 2.602807317 | 0.006417648 |
| ENSMUSG00000020303 |  | Stc2 | 120.1035488 | 54.58472445 | 1.133413089 | 0.039611272 |
| ENSMUSG00000020279 |  | Il9r | 2.68252407 | 13.51828432 | -2.167524913 | 0.042204249 |
| ENSMUSG00000047511 |  | Olfr1396 | 12.72772047 | 78.70074818 | -2.485094471 | 5.53884E-05 |
| ENSMUSG00000063564 |  | Col23a1 | 477.2520047 | 186.0128873 | 1.353869581 | 0.024711086 |
| ENSMUSG00000020388 |  | Pdlim4 | 380.1709578 | 159.5726906 | 1.260999514 | 0.01485025 |
| ENSMUSG00000020534 |  | Shmt1 | 2436.637658 | 1116.115852 | 1.125419116 | 0.002957064 |
| ENSMUSG00000010122 |  | Slc47a1 | 60.10031921 | 20.50463625 | 1.535115189 | 0.033727663 |
| ENSMUSG00000042826 |  | Fgf11 | 277.2652225 | 650.5717027 | -1.227491037 | 3.03088E-06 |
| ENSMUSG00000040963 |  | Asgr2 | 93.02212697 | 23.90591562 | 2.004643772 | 0.000955851 |
| ENSMUSG00000000317 |  | Bcl6b | 1131.1329 | 2304.571983 | -1.024637601 | 0.001140584 |
| ENSMUSG00000040543 |  | Pitpnm3 | 55.51493531 | 275.3966493 | -2.314027489 | 0.00347737 |
| ENSMUSG00000040471 |  | Ggt6 | 60.76386063 | 3.550356115 | 4.116957732 | 0.006413444 |
| ENSMUSG00000017453 |  | Pipox | 262.6602159 | 58.31815703 | 2.159674355 | 2.9793E-10 |
| ENSMUSG00000020826 |  | Nos2 | 156.3608346 | 417.3179452 | -1.405888493 | 1.45603E-05 |
| ENSMUSG00000018648 |  | Dusp14 | 115.15102 | 459.6734608 | -1.999311253 | 3.63837E-08 |
| ENSMUSG00000085628 |  | Appbp2os | 42.63761983 | 18.47071076 | 1.266494895 | 0.00837294 |
| ENSMUSG00000018427 |  | Ypel2 | 2324.95152 | 1129.092334 | 1.039734364 | 0.001029656 |
| ENSMUSG00000046719 |  | Nxph3 | 65.17783811 | 253.1698232 | -1.947983807 | 0.009416701 |
| ENSMUSG00000001240 |  | Ramp2 | 1032.306753 | 2787.348122 | -1.432949818 | 0.029394032 |
| ENSMUSG00000018411 |  | Mapt | 7930.669481 | 2935.350834 | 1.434133357 | 0.007035307 |
| ENSMUSG00000034652 |  | Cd300a | 118.5837187 | 273.6666213 | -1.193182185 | 0.023010784 |
| ENSMUSG00000020734 |  | Grin2c | 101.5381628 | 252.3194363 | -1.342263179 | 4.30952E-05 |
| ENSMUSG00000057286 |  | St6galnac2 | 318.7162217 | 657.2288375 | -1.045083528 | 0.039540522 |
| ENSMUSG00000053113 |  | Socs3 | 484.9247993 | 179.4466957 | 1.442086243 | 0.010672972 |
| ENSMUSG00000000056 |  | Narf | 1182.856229 | 557.6912807 | 1.079298135 | 2.08278E-06 |
| ENSMUSG00000039208 |  | Metrnl | 550.4445758 | 235.9657443 | 1.226683306 | 0.029660289 |
| ENSMUSG00000020607 |  | Fam84a | 1614.417902 | 426.6021818 | 1.920746848 | 0.041543061 |
| ENSMUSG00000020572 |  | Nampt | 18535.14869 | 6431.048681 | 1.526916238 | 3.38294E-07 |
| ENSMUSG00000096954 |  | Gdap10 | 627.8420743 | 285.7013864 | 1.137063264 | 0.039640982 |
| ENSMUSG00000048285 |  | Frmd6 | 4232.912449 | 8922.825401 | -1.075317275 | 0.000119384 |
| ENSMUSG00000042724 |  | Map3k9 | 67.63843461 | 158.5395162 | -1.257912559 | 0.001044154 |
| ENSMUSG00000021228 |  | Acot3 | 55.83774622 | 2.328222597 | 4.526876629 | 0.000509589 |
| ENSMUSG00000057963 |  | Itpk1 | 1737.642151 | 4118.722433 | -1.247133995 | 7.72882E-12 |
| ENSMUSG00000041481 |  | Serpina3g | 383.6506584 | 124.6064505 | 1.632182458 | 0.034147308 |
| ENSMUSG00000041323 |  | Ak7 | 9.476499822 | 25.53983147 | -1.532116519 | 0.046432073 |
| ENSMUSG00000076617 |  | Ighm | 527.4552103 | 1778.653338 | -1.75190431 | 3.2844E-06 |
| ENSMUSG00000021213 |  | Akr1c13 | 2579.713807 | 764.7711942 | 1.755229213 | 8.25762E-05 |
| ENSMUSG00000021214 |  | Akr1c18 | 231337.153 | 27.77182845 | 13.0308318 | 2.30439E-11 |
| ENSMUSG00000046159 |  | Chrm3 | 206.7879782 | 419.0069906 | -1.02763855 | 0.00734918 |
| ENSMUSG00000095105 |  | Edaradd | 20.81540996 | 5.084685444 | 2.124241605 | 0.02226491 |
| ENSMUSG00000055137 |  | Sugct | 449.7724902 | 210.0600444 | 1.094479971 | 0.006952036 |
| ENSMUSG00000053101 |  | Gpr141 | 39.09854652 | 78.42914855 | -1.014558492 | 0.01698229 |
| ENSMUSG00000064140 |  | Trim38 | 25.59846254 | 7.584325358 | 1.749483458 | 0.014776824 |
| ENSMUSG00000044734 |  | Serpinb1a | 9249.320226 | 740.4593806 | 3.643622534 | 5.86398E-10 |
| ENSMUSG00000051029 |  | Serpinb1b | 65.04366615 | 23.73522238 | 1.449815437 | 0.018107954 |
| ENSMUSG00000021384 |  | Susd3 | 35.83297615 | 296.7766395 | -3.061263704 | 0.002782156 |
| ENSMUSG00000051107 |  | Gm15440 | 104.3906351 | 17.27548797 | 2.634589345 | 4.73425E-07 |
| ENSMUSG00000021477 |  | Ctsl | 28482.20799 | 8733.309816 | 1.705473443 | 0.001515123 |
| ENSMUSG00000069170 |  | Adgrv1 | 292.1768985 | 120.2347225 | 1.279205414 | 0.001515123 |
| ENSMUSG00000021613 |  | Hapln1 | 772.4424145 | 1913.962205 | -1.309347227 | 0.002913306 |
| ENSMUSG00000034488 |  | Edil3 | 452.3687708 | 960.8135013 | -1.089554433 | 0.002833808 |
| ENSMUSG00000089940 |  | Gm4117 | 97.95228683 | 24.45715658 | 2.014356327 | 0.003419793 |
| ENSMUSG00000021620 |  | Acot12 | 22.26747408 | 119.1313912 | -2.366063985 | 1.85475E-06 |
| ENSMUSG00000021684 |  | Pde8b | 5121.236329 | 2218.875876 | 1.207172009 | 0.004051253 |
| ENSMUSG00000021680 |  | Crhbp | 0 | 8.639365444 | -5.73372369 | 0.006532907 |
| ENSMUSG00000091387 |  | Gcnt4 | 42.41121148 | 16.37636735 | 1.315881769 | 0.047769288 |
| ENSMUSG00000041817 |  | Fam169a | 542.3704544 | 129.3606698 | 2.074280762 | 0.001545473 |
| ENSMUSG00000071203 |  | Naip5 | 2802.111557 | 436.41632 | 2.683343995 | 0.001230538 |
| ENSMUSG00000021638 |  | Ocln | 507.2554115 | 167.0531482 | 1.601762008 | 0.000731267 |
| ENSMUSG00000041417 |  | Pik3r1 | 15323.04944 | 5866.217456 | 1.385088985 | 0.006905283 |
| ENSMUSG00000042743 |  | Sgtb | 996.4681698 | 3225.258151 | -1.695629561 | 1.24048E-06 |
| ENSMUSG00000055194 |  | Actbl2 | 0.787440537 | 17.32438239 | -4.001914913 | 0.041018639 |
| ENSMUSG00000021758 |  | Ddx4 | 109.4827275 | 30.92157913 | 1.864344177 | 0.000129583 |
| ENSMUSG00000015533 |  | Itga2 | 976.0414875 | 2145.166909 | -1.136513398 | 0.036873145 |
| ENSMUSG00000021728 |  | Emb | 11241.94079 | 1356.394871 | 3.051211634 | 0.0003242 |
| ENSMUSG00000095930 |  | Nim1k | 421.3332563 | 206.2284529 | 1.033549308 | 0.016061662 |
| ENSMUSG00000045201 |  | Lrrc3b | 130.1697086 | 280.7891669 | -1.106371891 | 0.002302283 |
| ENSMUSG00000021779 |  | Thrb | 7928.646481 | 1999.915619 | 1.987489642 | 0.000368444 |
| ENSMUSG00000050335 |  | Lgals3 | 193.6676966 | 470.0190095 | -1.272489671 | 0.0406752 |
| ENSMUSG00000046352 |  | Gjb2 | 997.8154168 | 32.64846739 | 4.934423299 | 5.75129E-09 |
| ENSMUSG00000043157 |  | Arl11 | 26.0531918 | 57.52605852 | -1.103968887 | 0.04193072 |
| ENSMUSG00000021944 |  | Gata4 | 82.94609825 | 32.60858501 | 1.363553226 | 0.013224108 |
| ENSMUSG00000022090 |  | Pdlim2 | 386.2332135 | 191.4459228 | 1.005123074 | 0.012185134 |
| ENSMUSG00000033730 |  | Egr3 | 75.58636659 | 253.1889446 | -1.729261364 | 0.045408795 |
| ENSMUSG00000034959 |  | Rubcnl | 30.66499009 | 94.11768468 | -1.658537443 | 4.28056E-06 |
| ENSMUSG00000048349 |  | Pou4f1 | 45.57973245 | 6.300810941 | 2.85600985 | 2.55375E-06 |
| ENSMUSG00000022112 |  | Gpc5 | 364.0539356 | 0.286373416 | 10.00742626 | 3.95632E-05 |
| ENSMUSG00000005268 |  | Prlr | 31045.0005 | 10373.29986 | 1.581424977 | 2.06866E-05 |
| ENSMUSG00000089678 |  | Agxt2 | 260.7342686 | 78.90555914 | 1.736712306 | 7.06917E-05 |
| ENSMUSG00000042895 |  | Abra | 87.21908391 | 4.688521882 | 4.230023097 | 0.001708759 |
| ENSMUSG00000022419 |  | Deptor | 24138.28648 | 9384.465509 | 1.362971754 | 9.99712E-06 |
| ENSMUSG00000013846 |  | St3gal1 | 11956.9374 | 3441.385482 | 1.797270583 | 5.10286E-05 |
| ENSMUSG00000000934 |  | Top1mt | 1204.312566 | 347.1353829 | 1.795427001 | 1.46871E-07 |
| ENSMUSG00000057346 |  | Apol9a | 71.53175773 | 173.1685777 | -1.251438967 | 0.021945253 |
| ENSMUSG00000016942 |  | Tmprss6 | 107.3989334 | 24.32598591 | 2.223485143 | 6.14573E-06 |
| ENSMUSG00000044216 |  | Kcnj4 | 9.059611625 | 74.34555344 | -2.984624664 | 2.52155E-06 |
| ENSMUSG00000044021 |  | Muc19 | 37.95454851 | 1.562559938 | 4.506157742 | 0.031678682 |
| ENSMUSG00000036273 |  | Lrrk2 | 12548.7801 | 2340.416009 | 2.423030218 | 1.12759E-06 |
| ENSMUSG00000023031 |  | Cela1 | 44365.5915 | 1737.406646 | 4.674450508 | 5.2656E-05 |
| ENSMUSG00000049382 |  | Krt8 | 80.27077532 | 10.92819083 | 2.868078067 | 0.044313608 |
| ENSMUSG00000054939 |  | Zfp174 | 1065.377675 | 278.5671199 | 1.93606936 | 4.67274E-07 |
| ENSMUSG00000039457 |  | Ppl | 133.0877826 | 386.6075286 | -1.533390783 | 0.000727456 |
| ENSMUSG00000008393 |  | Carhsp1 | 7593.019938 | 3741.589559 | 1.021254112 | 8.90656E-06 |
| ENSMUSG00000022773 |  | Ypel1 | 336.2893641 | 140.3498949 | 1.270005117 | 0.002312973 |
| ENSMUSG00000022758 |  | P2rx6 | 25.40328159 | 109.0357697 | -2.135400316 | 0.000870362 |
| ENSMUSG00000022756 |  | Slc7a4 | 276.3215051 | 561.4442829 | -1.026398415 | 0.002031771 |
| ENSMUSG00000003526 |  | Prodh | 892.3547806 | 271.769494 | 1.711550782 | 0.000764328 |
| ENSMUSG00000041205 |  | Map6d1 | 231.9465647 | 41.37672027 | 2.49753123 | 2.90499E-05 |
| ENSMUSG00000051146 |  | Camk2n2 | 501.6065282 | 1594.241084 | -1.670289203 | 3.89291E-11 |
| ENSMUSG00000022871 |  | Fetub | 5654.851885 | 181.6172823 | 4.962835187 | 2.55189E-09 |
| ENSMUSG00000109783 |  | Gm45338 | 160.1421432 | 44.24213119 | 1.86774564 | 0.008754968 |
| ENSMUSG00000022514 |  | Il1rap | 1141.916315 | 502.1390529 | 1.18744029 | 0.010935362 |
| ENSMUSG00000035578 |  | Iqcg | 63.94304071 | 134.109145 | -1.046039524 | 0.021945253 |
| ENSMUSG00000052133 |  | Sema5b | 663.2861729 | 2527.104721 | -1.933077107 | 5.75129E-09 |
| ENSMUSG00000022865 |  | Cxadr | 1953.889964 | 322.1521691 | 2.597353265 | 2.14974E-06 |
| ENSMUSG00000022893 |  | Adamts1 | 4437.837221 | 10003.30867 | -1.171913606 | 0.003419793 |
| ENSMUSG00000022948 |  | Setd4 | 208.1875683 | 98.15057296 | 1.085609188 | 0.003991491 |
| ENSMUSG00000023827 |  | Agpat4 | 732.620907 | 1935.324039 | -1.3989787 | 1.87445E-05 |
| ENSMUSG00000023905 |  | Tnfrsf12a | 1689.701019 | 3393.014589 | -1.005168606 | 0.000668151 |
| ENSMUSG00000024155 |  | Meiob | 39.31368035 | 99.25629485 | -1.347873595 | 0.031275441 |
| ENSMUSG00000073435 |  | Nme3 | 245.2496705 | 95.92265067 | 1.354054469 | 0.005276324 |
| ENSMUSG00000023979 |  | Guca1b | 93.49829517 | 24.46388867 | 1.875744769 | 4.37008E-08 |
| ENSMUSG00000051682 |  | Treml4 | 18.43357079 | 75.403366 | -2.062122186 | 5.8606E-05 |
| ENSMUSG00000023992 |  | Trem2 | 44.48379929 | 112.3975133 | -1.316765305 | 0.035129306 |
| ENSMUSG00000024164 |  | C3 | 75060.34616 | 7917.975884 | 3.244824171 | 0.025148895 |
| ENSMUSG00000050612 |  | Txndc2 | 1012.62732 | 326.0852469 | 1.637535822 | 0.004252586 |
| ENSMUSG00000038541 |  | Srd5a2 | 1402.802375 | 45861.13088 | -5.030388748 | 4.95502E-33 |
| ENSMUSG00000059811 |  | Atl2 | 13090.18606 | 5737.996464 | 1.189879653 | 0.008989828 |
| ENSMUSG00000024247 |  | Pkdcc | 3989.838275 | 1304.602932 | 1.614005648 | 9.106E-06 |
| ENSMUSG00000061013 |  | Mkx | 792.3813837 | 275.7504427 | 1.524489652 | 0.047742668 |
| ENSMUSG00000042942 |  | Greb1l | 4568.534854 | 685.7871328 | 2.737174097 | 3.53445E-07 |
| ENSMUSG00000033107 |  | Rnf125 | 576.6615567 | 184.0856152 | 1.644614822 | 0.026273934 |
| ENSMUSG00000024277 |  | Mapre2 | 5266.037973 | 12713.91676 | -1.272432751 | 6.42077E-22 |
| ENSMUSG00000042834 |  | Nrep | 47904.50347 | 1542.495149 | 4.956872638 | 3.18502E-10 |
| ENSMUSG00000024486 |  | Hbegf | 186.0317854 | 425.8920939 | -1.188298154 | 0.000888828 |
| ENSMUSG00000024427 |  | Spry4 | 1226.894615 | 2638.477201 | -1.102668164 | 9.35879E-05 |
| ENSMUSG00000056671 |  | Prelid2 | 64.38530139 | 27.73795701 | 1.21832986 | 0.012159828 |
| ENSMUSG00000073565 |  | Prr16 | 1914.089099 | 158.2644097 | 3.60084278 | 2.13333E-06 |
| ENSMUSG00000024600 |  | Slc27a6 | 270.6869837 | 22.71849854 | 3.573778044 | 4.3083E-07 |
| ENSMUSG00000034320 |  | Slc26a2 | 2161.921286 | 954.4697537 | 1.178888144 | 0.002003016 |
| ENSMUSG00000024575 |  | Pde6a | 55.04112382 | 1.266696685 | 5.963865879 | 8.68664E-05 |
| ENSMUSG00000045730 |  | Adrb2 | 425.8467184 | 93.76849543 | 2.17461397 | 0.001898099 |
| ENSMUSG00000038121 |  | Fam210a | 16006.01369 | 5996.41637 | 1.416353237 | 5.52511E-11 |
| ENSMUSG00000044646 |  | Zbtb7c | 517.5287088 | 126.5411669 | 2.037423797 | 0.000110115 |
| ENSMUSG00000024552 |  | Slc14a2 | 23.96726475 | 0 | 7.542169176 | 0.001683447 |
| ENSMUSG00000073514 |  | Dok6 | 52.0001209 | 9.770442219 | 2.380801208 | 0.003767492 |
| ENSMUSG00000024885 |  | Aldh3b1 | 3741.425049 | 1293.369415 | 1.533527969 | 0.000788581 |
| ENSMUSG00000024912 |  | Fosl1 | 10.91132259 | 62.11668831 | -2.43771354 | 0.000238009 |
| ENSMUSG00000024818 |  | Slc25a45 | 890.7301112 | 424.5826288 | 1.070614023 | 0.010235625 |
| ENSMUSG00000047787 |  | Flrt1 | 135.3826249 | 385.1513876 | -1.514291774 | 4.67303E-05 |
| ENSMUSG00000024672 |  | Ms4a7 | 112.6209511 | 230.6771047 | -1.023404065 | 0.027901324 |
| ENSMUSG00000024731 |  | Ms4a10 | 219.486209 | 12.41747181 | 4.215650853 | 7.46677E-07 |
| ENSMUSG00000024712 |  | Rfk | 13681.27584 | 4010.979116 | 1.770265917 | 1.0395E-06 |
| ENSMUSG00000037847 |  | Nmrk1 | 242.1470083 | 687.4051936 | -1.500447675 | 6.40327E-06 |
| ENSMUSG00000058624 |  | Gda | 3011.746279 | 792.9809651 | 1.925088827 | 0.00741523 |
| ENSMUSG00000052085 |  | Dock8 | 4442.877036 | 809.9868295 | 2.455719076 | 8.21106E-07 |
| ENSMUSG00000024770 |  | Lipn | 48.32872534 | 1.002306955 | 5.370196888 | 0.003993886 |
| ENSMUSG00000050370 |  | Ch25h | 17.53874516 | 101.8701668 | -2.495090849 | 0.000242136 |
| ENSMUSG00000041180 |  | Hectd2 | 231.1538567 | 504.8450846 | -1.125573932 | 0.009666023 |
| ENSMUSG00000048612 |  | Myof | 1330.955075 | 2933.630958 | -1.140522817 | 3.2844E-06 |
| ENSMUSG00000025017 |  | Pik3ap1 | 346.9997398 | 720.180567 | -1.056048215 | 0.031787064 |
| ENSMUSG00000025020 |  | Slit1 | 718.9643747 | 2539.742004 | -1.821401298 | 0.00516669 |
| ENSMUSG00000034765 |  | Dusp5 | 188.3724968 | 479.4797084 | -1.340749891 | 0.001079219 |
| ENSMUSG00000024978 |  | Gpam | 20754.9532 | 7084.221649 | 1.550874664 | 0.003796224 |
| ENSMUSG00000073295 |  | Nudt11 | 1254.46428 | 394.8030424 | 1.669239566 | 2.77139E-07 |
| ENSMUSG00000037369 |  | Kdm6a | 3971.569192 | 1915.918375 | 1.050538767 | 2.52461E-33 |
| ENSMUSG00000017057 |  | Il13ra1 | 2084.209515 | 844.2641809 | 1.303303483 | 9.25654E-05 |
| ENSMUSG00000044400 |  | Sowahd | 3.610022909 | 30.36228913 | -3.109207413 | 0.001121136 |
| ENSMUSG00000037010 |  | Apln | 256.3500551 | 1064.767999 | -2.055956905 | 0.020216932 |
| ENSMUSG00000031104 |  | Rab33a | 341.7738881 | 145.7303728 | 1.220073285 | 0.036701074 |
| ENSMUSG00000031111 |  | Igsf1 | 59.90105258 | 170.5602065 | -1.511728968 | 0.011721919 |
| ENSMUSG00000031119 |  | Gpc4 | 6836.874864 | 2746.444644 | 1.315631207 | 5.5003E-06 |
| ENSMUSG00000031189 |  | Aff2 | 440.4149857 | 150.8963538 | 1.546305041 | 0.007277555 |
| ENSMUSG00000019558 |  | Slc6a8 | 2468.202949 | 4936.977079 | -1.000717413 | 5.45082E-16 |
| ENSMUSG00000025056 |  | Nr0b1 | 312.97002 | 52.70566247 | 2.551558967 | 0.000761424 |
| ENSMUSG00000045103 |  | Dmd | 5629.618634 | 1945.742345 | 1.532555558 | 1.24897E-05 |
| ENSMUSG00000034457 |  | Eda2r | 597.9592224 | 150.0989861 | 1.999012132 | 4.12629E-06 |
| ENSMUSG00000046532 |  | Ar | 24576.91184 | 9478.795208 | 1.374542297 | 6.79288E-05 |
| ENSMUSG00000051159 |  | Cited1 | 29.85694984 | 155.886536 | -2.367984219 | 0.001290145 |
| ENSMUSG00000086503 |  | Xist | 39253.42361 | 6.457572468 | 12.45252019 | 0 |
| ENSMUSG00000072944 |  | Nup62cl | 550.4720794 | 1115.956454 | -1.023613698 | 0.000549962 |
| ENSMUSG00000031274 |  | Col4a5 | 3254.85093 | 1256.191826 | 1.373004391 | 0.018959946 |
| ENSMUSG00000031292 |  | Cdkl5 | 2802.462748 | 1398.875221 | 1.002147757 | 4.2303E-06 |
| ENSMUSG00000031380 |  | Vegfd | 4885.156172 | 1770.975168 | 1.464031245 | 0.008526618 |
| ENSMUSG00000056673 |  | Kdm5d | 2.426657551 | 1862.509994 | -10.06815281 | 5.1668E-70 |
| ENSMUSG00000069049 |  | Eif2s3y | 7.099828496 | 2411.296885 | -8.56919239 | 1.23878E-30 |
| ENSMUSG00000068457 |  | Uty | 3.561118842 | 2055.553532 | -9.530814674 | 3.21855E-90 |
| ENSMUSG00000069045 |  | Ddx3y | 15.57010358 | 8159.53475 | -9.05131258 | 1.64332E-14 |
| ENSMUSG00000064356 |  | mt-Atp8 | 2567.115773 | 12823.66983 | -2.320635906 | 0.00376281 |
| ENSMUSG00000034139 |  | Serpini2 | 781.9152259 | 0 | 29.95977303 | 2.92562E-05 |
| ENSMUSG00000083079 |  | Amy2b | 2016.781751 | 0 | 31.97808435 | 5.69374E-06 |
| ENSMUSG00000070360 |  | Amy2a1 | 305.3254984 | 0 | 28.04373819 | 0.000115072 |
| ENSMUSG00000023433 |  | Cela3b | 8376.68064 | 0 | 34.31311881 | 7.84906E-07 |
| ENSMUSG00000029273 |  | Sult1d1 | 5.249603579 | 0 | 19.88666078 | 0.004870619 |
| ENSMUSG00000029522 |  | Pla2g1b | 1175.620983 | 0 | 29.89309647 | 3.04999E-05 |
| ENSMUSG00000054446 |  | Cpa1 | 23823.31038 | 0 | 45.33365609 | 1.14869E-13 |
| ENSMUSG00000054106 |  | Try4 | 13657.12559 | 0 | 38.79598935 | 1.09547E-08 |
| ENSMUSG00000036938 |  | Try5 | 6461.122851 | 0 | 34.36189478 | 7.58674E-07 |
| ENSMUSG00000057163 |  | Prss2 | 11439.62469 | 0 | 41.1326994 | 9.84808E-10 |
| ENSMUSG00000071356 |  | Reg3b | 377.6370358 | 0 | 29.03383552 | 5.67647E-05 |
| ENSMUSG00000068341 |  | Reg3d | 404.1837088 | 0 | 29.02138865 | 5.71586E-05 |
| ENSMUSG00000023140 |  | Reg2 | 825.4125418 | 0 | 30.5753907 | 1.76496E-05 |
| ENSMUSG00000059654 |  | Reg1 | 14232.49484 | 0 | 37.52183661 | 3.85484E-08 |
| ENSMUSG00000057465 |  | Saa2 | 0.262480179 | 0 | 30.35515575 | 3.21086E-05 |
| ENSMUSG00000030954 |  | Gp2 | 4422.980848 | 0 | 35.10628329 | 4.00043E-07 |
| ENSMUSG00000039488 |  | Cntn5 | 0 | 0.634969799 | -30.66586754 | 3.31392E-09 |
| ENSMUSG00000015890 |  | Amdhd1 | 5.512083758 | 0 | 19.63906578 | 0.016343707 |
| ENSMUSG00000020429 |  | Igfbp1 | 14.30516975 | 0 | 20.89951375 | 5.3519E-06 |
| ENSMUSG00000061947 |  | Serpina10 | 14.90335593 | 0 | 21.13373314 | 0.000386443 |
| ENSMUSG00000035896 |  | Rnase1 | 10628.95813 | 0 | 30.8365063 | 4.48594E-14 |
| ENSMUSG00000024225 |  | Clps | 6955.445202 | 0 | 34.51918255 | 6.78218E-07 |
| ENSMUSG00000024028 |  | Tff2 | 909.0891206 | 0 | 31.27060335 | 9.99712E-06 |
| ENSMUSG00000024503 |  | Spink1 | 485.2777045 | 0 | 29.38226513 | 4.44311E-05 |
| ENSMUSG00000024868 |  | Dkk1 | 1.952805814 | 0 | 32.01956478 | 6.26638E-06 |
| ENSMUSG00000042248 |  | Cyp2c37 | 9.055566173 | 0 | 20.46377633 | 0.010944015 |
| ENSMUSG00000046008 |  | Pnlip | 72249.37406 | 0 | 33.63013014 | 1.26041E-08 |
| ENSMUSG00000042179 |  | Pnliprp1 | 19339.0054 | 0 | 38.93440926 | 9.74956E-09 |
| ENSMUSG00000025091 |  | Pnliprp2 | 3490.867281 | 0 | 31.83017312 | 6.33677E-06 |
