# Supplementary material for: Early life microbiome disbalance impacts neuroendocrine outcomes in pre-pubertal mice in a sexually dimorphic manner
Source: Front Microbiol. 2025 Jun 20;16:1504513. doi: 10.3389/fmicb.2025.1504513 (PMC12277575; doi:10.3389/fmicb.2025.1504513)
Supplement: Supplementary file 1 [file Supplementary_file_1.zip › Supplementary Table 7.DOCX]

**Supplemental Table 7: Sexually dimorphic DEG in adrenal medullae of Abx groups** (female Abx vs male Abx, mean values), Log2 FC=2 cutoff. Only protein coding transcripts are included.

| **Gene ID** |  | **Gene Name** | **F Abx** | **M Abx** | **log2FoldChange** | **padj** |
| --- | --- | --- | --- | --- | --- | --- |
|  |  |  |  |  |  |  |
| ENSMUSG00000099032 |  | Tcf24 | 1191.988956 | 328.3635212 | 1.858727851 | 0.004357 |
| ENSMUSG00000025934 |  | Gsta3 | 2427.57165 | 591.8421553 | 2.035363519 | 0.035938 |
| ENSMUSG00000047343 |  | Mettl21c | 0 | 14.33068792 | -5.851565752 | 0.006118 |
| ENSMUSG00000101337 |  | Dnah7c | 58.96800619 | 152.477901 | -1.370758396 | 0.033766 |
| ENSMUSG00000025981 |  | Coq10b | 4837.205812 | 10061.22875 | -1.056447002 | 2.8E-05 |
| ENSMUSG00000038242 |  | Aox4 | 2.273515528 | 25.36353643 | -3.472509849 | 0.017747 |
| ENSMUSG00000026012 |  | Cd28 | 30.84773217 | 71.31661052 | -1.203191945 | 0.020751 |
| ENSMUSG00000026014 |  | Raph1 | 11460.9294 | 29717.84401 | -1.374633093 | 2.89E-05 |
| ENSMUSG00000027520 |  | Zdbf2 | 2648.9803 | 7093.646119 | -1.421147937 | 0.000728 |
| ENSMUSG00000062209 |  | Erbb4 | 154.0024655 | 34.67148931 | 2.130602755 | 0.023265 |
| ENSMUSG00000073650 |  | Catip | 13.66638519 | 32.35398205 | -1.224690182 | 0.044329 |
| ENSMUSG00000051703 |  | Tmem198 | 68.89079373 | 144.1393562 | -1.062677277 | 0.021911 |
| ENSMUSG00000089960 |  | Ugt1a1 | 1102.558143 | 68.03071293 | 4.021202484 | 0.04349 |
| ENSMUSG00000047443 |  | Erfe | 58.96152463 | 289.1593817 | -2.294913731 | 0.001193 |
| ENSMUSG00000042510 |  | AA986860 | 203.2215712 | 801.9476888 | -1.978511808 | 3.47E-10 |
| ENSMUSG00000042251 |  | Pm20d1 | 724.8272811 | 349.4119732 | 1.047591802 | 0.003114 |
| ENSMUSG00000009772 |  | Nuak2 | 144.6648455 | 341.3134863 | -1.234808535 | 0.00011 |
| ENSMUSG00000070644 |  | Etnk2 | 169.2416679 | 902.7343041 | -2.415081104 | 0.010592 |
| ENSMUSG00000102976 |  | Zc3h11a | 5546.669835 | 2075.561351 | 1.418054069 | 0.000274 |
| ENSMUSG00000026452 |  | Syt2 | 282.5932344 | 8.625954896 | 5.017036306 | 0.001271 |
| ENSMUSG00000026358 |  | Rgs1 | 67.57162799 | 151.4103549 | -1.166052757 | 0.035929 |
| ENSMUSG00000097754 |  | Ptgs2os2 | 101.2333361 | 24.01953111 | 2.055063445 | 0.027965 |
| ENSMUSG00000006014 |  | Prg4 | 185.810148 | 38.96649301 | 2.263844498 | 0.006611 |
| ENSMUSG00000033544 |  | Angptl1 | 242.8615327 | 79.02459982 | 1.597448422 | 2.62E-05 |
| ENSMUSG00000026601 |  | Axdnd1 | 83.8429292 | 308.5409094 | -1.882521647 | 0.001342 |
| ENSMUSG00000026579 |  | F5 | 694.2391975 | 89.31226052 | 2.96002029 | 0.000728 |
| ENSMUSG00000040612 |  | Ildr2 | 711.0096781 | 1862.255041 | -1.388744419 | 3.63E-07 |
| ENSMUSG00000026675 |  | Hsd17b7 | 8535.290851 | 2080.204296 | 2.036562617 | 0.000696 |
| ENSMUSG00000006403 |  | Adamts4 | 166.3890587 | 367.395559 | -1.143332602 | 0.028309 |
| ENSMUSG00000004707 |  | Ly9 | 39.11142697 | 118.4950509 | -1.606970171 | 0.004742 |
| ENSMUSG00000015355 |  | Cd48 | 60.80742682 | 149.143942 | -1.297346217 | 0.044869 |
| ENSMUSG00000038147 |  | Cd84 | 157.1367675 | 331.8805381 | -1.081759052 | 0.013684 |
| ENSMUSG00000049598 |  | Vsig8 | 21.76363691 | 1.239999263 | 3.843007862 | 0.028694 |
| ENSMUSG00000038949 |  | Cnst | 2647.023026 | 1114.737892 | 1.247675091 | 0.00073 |
| ENSMUSG00000055676 |  | Gm5069 | 126.9471123 | 53.52928077 | 1.246583736 | 0.00017 |
| ENSMUSG00000026630 |  | Batf3 | 30.20945772 | 82.56933769 | -1.45118737 | 0.009905 |
| ENSMUSG00000026628 |  | Atf3 | 122.088166 | 576.474952 | -2.238416475 | 0.003787 |
| ENSMUSG00000016262 |  | Sertad4 | 897.046503 | 366.5364416 | 1.296974128 | 0.000214 |
| ENSMUSG00000026672 |  | Optn | 2380.683967 | 5936.124166 | -1.318094342 | 9.95E-08 |
| ENSMUSG00000039145 |  | Camk1d | 992.1058762 | 2106.438648 | -1.086302039 | 0.04659 |
| ENSMUSG00000045319 |  | Proser2 | 345.9677206 | 94.4294583 | 1.868351361 | 5.41E-06 |
| ENSMUSG00000048038 |  | Ccdc187 | 181.5838757 | 617.7946704 | -1.766111367 | 0.03469 |
| ENSMUSG00000026818 |  | Cel | 0.918370742 | 0 | 35.74620689 | 4.12E-07 |
| ENSMUSG00000001864 |  | Aif1l | 332.987157 | 1066.046993 | -1.678110184 | 7.07E-05 |
| ENSMUSG00000050592 |  | Fam78a | 124.2490656 | 54.62677585 | 1.182177108 | 0.020985 |
| ENSMUSG00000026885 |  | Ttll11 | 238.1600593 | 76.24366611 | 1.642751563 | 0.030857 |
| ENSMUSG00000106994 |  | Gm10829 | 149.7971189 | 15.06487803 | 3.284455998 | 0.040039 |
| ENSMUSG00000050447 |  | Lypd6 | 876.3560711 | 111.3391816 | 2.973037011 | 2.67E-07 |
| ENSMUSG00000085862 |  | Gm13483 | 14.03880594 | 44.25298903 | -1.654457782 | 0.038549 |
| ENSMUSG00000053475 |  | Tnfaip6 | 78.52551779 | 366.068744 | -2.220427501 | 0.002052 |
| ENSMUSG00000026950 |  | Neb | 917.0886011 | 120.411684 | 2.926956532 | 0.032201 |
| ENSMUSG00000086447 |  | Gm13522 | 8.139712325 | 0.495999705 | 3.481011891 | 0.03469 |
| ENSMUSG00000026836 |  | Acvr1 | 2654.227234 | 8659.287502 | -1.706024512 | 1.83E-08 |
| ENSMUSG00000027004 |  | Frzb | 21625.79611 | 2166.668752 | 3.31889419 | 9.8E-06 |
| ENSMUSG00000102091 |  | Olfr1034 | 80.0263362 | 27.19928868 | 1.548995154 | 0.045909 |
| ENSMUSG00000045392 |  | Olfr1033 | 478.8621032 | 198.2047333 | 1.271374327 | 0.030847 |
| ENSMUSG00000027257 |  | Pacsin3 | 1268.403484 | 588.3622252 | 1.10884735 | 2.35E-10 |
| ENSMUSG00000027230 |  | Creb3l1 | 610.3467983 | 1733.377571 | -1.506337444 | 1.65E-25 |
| ENSMUSG00000027222 |  | Pex16 | 2184.689517 | 778.9349376 | 1.487723826 | 1.27E-05 |
| ENSMUSG00000012350 |  | Ehf | 107.2860705 | 16.48902776 | 2.660955242 | 0.038589 |
| ENSMUSG00000027187 |  | Cat | 21635.07309 | 10323.27498 | 1.067463701 | 0.007326 |
| ENSMUSG00000027171 |  | Prrg4 | 454.1730133 | 2245.039338 | -2.305526448 | 2.69E-09 |
| ENSMUSG00000005973 |  | Rcn1 | 10436.30965 | 3710.747144 | 1.491761052 | 7.83E-12 |
| ENSMUSG00000040093 |  | Bmf | 1247.514564 | 546.5875093 | 1.189298618 | 4.37E-05 |
| ENSMUSG00000078137 |  | Ankrd63 | 13.57077696 | 0.495999705 | 4.28460599 | 0.015726 |
| ENSMUSG00000027313 |  | Chac1 | 311.8562189 | 88.91515882 | 1.799575374 | 0.000555 |
| ENSMUSG00000070719 |  | Pla2g4d | 34.53530149 | 5.436563591 | 2.644564806 | 0.005646 |
| ENSMUSG00000054484 |  | Tmem62 | 1390.401822 | 672.1763892 | 1.047822553 | 0.017967 |
| ENSMUSG00000033498 |  | Strc | 0 | 15.99284557 | -5.956530635 | 0.000264 |
| ENSMUSG00000033213 |  | AA467197 | 59.3630398 | 14.86500326 | 1.977669843 | 0.006533 |
| ENSMUSG00000027360 |  | Hdc | 1236.159524 | 322.7123966 | 1.935391153 | 0.029054 |
| ENSMUSG00000027399 |  | Il1a | 0 | 8.830274938 | -5.176909184 | 0.008774 |
| ENSMUSG00000037902 |  | Sirpa | 6567.745627 | 3215.477334 | 1.030217496 | 0.009746 |
| ENSMUSG00000027438 |  | Napb | 1683.651081 | 746.3333675 | 1.173236077 | 0.02487 |
| ENSMUSG00000027442 |  | Cst8 | 2063.497234 | 129.1363212 | 3.991983907 | 0.000246 |
| ENSMUSG00000044863 |  | Defb36 | 3.198569361 | 17.46603928 | -2.448576713 | 0.018693 |
| ENSMUSG00000032715 |  | Trib3 | 364.7530722 | 45.31215846 | 3.001535189 | 0.000528 |
| ENSMUSG00000027602 |  | Map1lc3a | 8971.763132 | 4011.159529 | 1.160923375 | 7.83E-12 |
| ENSMUSG00000027611 |  | Procr | 1239.595857 | 2867.266515 | -1.209623041 | 0.006521 |
| ENSMUSG00000038180 |  | Spag4 | 41.21777925 | 117.6759498 | -1.524791945 | 0.000238 |
| ENSMUSG00000027412 |  | Lpin3 | 2095.443073 | 781.6696073 | 1.422257151 | 1.6E-08 |
| ENSMUSG00000074607 |  | Tox2 | 175.865624 | 445.1603213 | -1.340153439 | 0.003308 |
| ENSMUSG00000042845 |  | Wfdc12 | 39.89204598 | 1.239999263 | 4.799057378 | 0.028227 |
| ENSMUSG00000070533 |  | Wfdc8 | 3.58383766 | 65.41218521 | -4.182748531 | 0.001512 |
| ENSMUSG00000053166 |  | Cdh22 | 66.57605641 | 306.1643394 | -2.201964234 | 5.33E-05 |
| ENSMUSG00000006800 |  | Sulf2 | 3784.79854 | 7650.829394 | -1.015424493 | 2.47E-05 |
| ENSMUSG00000056501 |  | Cebpb | 3792.200856 | 1397.535489 | 1.439294633 | 5.95E-11 |
| ENSMUSG00000008999 |  | Bmp7 | 1678.826304 | 4178.014497 | -1.315269664 | 0.0018 |
| ENSMUSG00000069094 |  | Pde7a | 4318.250367 | 1851.351905 | 1.220991642 | 2.17E-05 |
| ENSMUSG00000027698 |  | Nceh1 | 13688.14585 | 6607.273371 | 1.050701036 | 1.23E-06 |
| ENSMUSG00000045031 |  | Cetn4 | 155.2232283 | 342.0678728 | -1.139253569 | 3.61E-06 |
| ENSMUSG00000046743 |  | Fat4 | 3528.314721 | 1522.539518 | 1.212716433 | 0.00209 |
| ENSMUSG00000090919 |  | Pabpc4l | 181.6522329 | 60.54842508 | 1.557752364 | 0.000424 |
| ENSMUSG00000102544 |  | Gm5103 | 34.02481191 | 6.0767328 | 2.394549979 | 0.00449 |
| ENSMUSG00000074604 |  | Mgst2 | 1559.13945 | 533.5054634 | 1.546715659 | 0.029059 |
| ENSMUSG00000027796 |  | Smad9 | 5739.968803 | 2556.054644 | 1.16619935 | 0.000153 |
| ENSMUSG00000034009 |  | Rxfp1 | 218.8510923 | 2.461862476 | 6.443376062 | 0.030847 |
| ENSMUSG00000027955 |  | Fam198b | 1774.202418 | 4859.49547 | -1.453728473 | 2.98E-05 |
| ENSMUSG00000041842 |  | Fhdc1 | 739.5126772 | 135.4924889 | 2.435429034 | 9.09E-06 |
| ENSMUSG00000041750 |  | Cd1d2 | 31.75633759 | 2.479998526 | 3.441187139 | 0.013289 |
| ENSMUSG00000028076 |  | Cd1d1 | 16672.18175 | 1411.17792 | 3.562039122 | 3.42E-09 |
| ENSMUSG00000004891 |  | Nes | 2917.362388 | 6485.568751 | -1.152692485 | 0.001136 |
| ENSMUSG00000059743 |  | Fdps | 14944.02495 | 4126.867701 | 1.856334915 | 0.003444 |
| ENSMUSG00000027908 |  | Tchhl1 | 47.11955202 | 8.39276045 | 2.480554823 | 0.034618 |
| ENSMUSG00000052415 |  | Tchh | 215.9505283 | 49.60201729 | 2.109809139 | 0.006312 |
| ENSMUSG00000027859 |  | Ngf | 36.72009342 | 83.80081784 | -1.177344475 | 0.021827 |
| ENSMUSG00000027962 |  | Vcam1 | 2071.904962 | 4143.714067 | -1.000166617 | 0.011329 |
| ENSMUSG00000033386 |  | Frrs1 | 1578.140748 | 522.3672749 | 1.59379984 | 8.97E-05 |
| ENSMUSG00000033308 |  | Dpyd | 2843.774983 | 1420.044509 | 1.000927634 | 9.13E-05 |
| ENSMUSG00000028125 |  | Abca4 | 72.46506761 | 26.70292077 | 1.403863065 | 0.001531 |
| ENSMUSG00000027983 |  | Cyp2u1 | 497.4259911 | 211.882547 | 1.229920203 | 0.032933 |
| ENSMUSG00000037922 |  | Bank1 | 18.60680798 | 79.32126867 | -2.089080601 | 0.005206 |
| ENSMUSG00000028159 |  | Dapp1 | 493.8878432 | 995.0988976 | -1.010086554 | 0.009134 |
| ENSMUSG00000074207 |  | Adh1 | 182597.7004 | 67074.29225 | 1.444827056 | 0.021108 |
| ENSMUSG00000028273 |  | Pdlim5 | 9070.144642 | 4377.056803 | 1.05061226 | 0.002591 |
| ENSMUSG00000040213 |  | Kyat3 | 625.6268942 | 295.0274245 | 1.085666678 | 0.049779 |
| ENSMUSG00000028266 |  | Lmo4 | 3407.057816 | 1547.291507 | 1.137917878 | 1.11E-07 |
| ENSMUSG00000028179 |  | Cth | 1258.699067 | 345.1146999 | 1.865453127 | 6.45E-05 |
| ENSMUSG00000041261 |  | Car8 | 3683.280004 | 354.1397351 | 3.376965075 | 0.000371 |
| ENSMUSG00000028238 |  | Atp6v0d2 | 5.897653269 | 44.97086409 | -2.932451162 | 0.045959 |
| ENSMUSG00000073988 |  | Ttpa | 486.0106712 | 231.7658435 | 1.059879956 | 0.030252 |
| ENSMUSG00000055761 |  | Nkain3 | 94.02520446 | 14.71675669 | 2.63876469 | 0.021373 |
| ENSMUSG00000054659 |  | Pm20d2 | 440.7543351 | 118.8431923 | 1.881364452 | 1.63E-08 |
| ENSMUSG00000044813 |  | Shb | 2518.383498 | 995.7878786 | 1.337770921 | 5.84E-08 |
| ENSMUSG00000086712 |  | AI427809 | 131.0886242 | 39.99756126 | 1.690789547 | 0.005551 |
| ENSMUSG00000015243 |  | Abca1 | 29980.38597 | 10274.02361 | 1.544865109 | 0.001085 |
| ENSMUSG00000028378 |  | Ptgr1 | 859.3763965 | 2096.360888 | -1.286576118 | 0.001347 |
| ENSMUSG00000043753 |  | Dmrta1 | 149.841826 | 22.60703864 | 2.687574317 | 0.009959 |
| ENSMUSG00000008489 |  | Elavl2 | 555.2284271 | 63.68561747 | 3.124133382 | 0.023442 |
| ENSMUSG00000052684 |  | Jun | 5666.094442 | 13396.38277 | -1.241415928 | 3.28E-06 |
| ENSMUSG00000078612 |  | Fyb2 | 16.32352713 | 45.05546379 | -1.476080649 | 0.005555 |
| ENSMUSG00000085873 |  | Ttc39aos1 | 84.85434458 | 226.7453442 | -1.417369784 | 0.002879 |
| ENSMUSG00000070806 |  | Zmynd12 | 45.95314607 | 94.05689782 | -1.032863072 | 0.03686 |
| ENSMUSG00000070803 |  | Cited4 | 452.7691714 | 100.5765261 | 2.165434016 | 0.016359 |
| ENSMUSG00000032744 |  | Heyl | 622.9535914 | 1366.227812 | -1.132726979 | 0.009908 |
| ENSMUSG00000028778 |  | Hcrtr1 | 78.56829549 | 23.55794569 | 1.750717166 | 0.027289 |
| ENSMUSG00000028893 |  | Sesn2 | 1369.953557 | 555.173135 | 1.302758361 | 0.012267 |
| ENSMUSG00000028885 |  | Smpdl3b | 1151.691938 | 93.67989562 | 3.611490306 | 0.000313 |
| ENSMUSG00000028836 |  | Slc30a2 | 816.3764795 | 1751.762528 | -1.10188683 | 0.001688 |
| ENSMUSG00000036896 |  | C1qc | 822.6959376 | 1690.374374 | -1.039176914 | 0.006246 |
| ENSMUSG00000028737 |  | Aldh4a1 | 6618.884866 | 2683.130911 | 1.302471284 | 0.003928 |
| ENSMUSG00000028927 |  | Padi2 | 644.7525806 | 119.797805 | 2.424672274 | 0.002429 |
| ENSMUSG00000066026 |  | Dhrs3 | 1949.72234 | 810.1927204 | 1.266289033 | 0.001512 |
| ENSMUSG00000028599 |  | Tnfrsf1b | 466.2413143 | 986.7532035 | -1.083476054 | 2.79E-06 |
| ENSMUSG00000078502 |  | Gm13212 | 159.4591097 | 54.38136022 | 1.529917504 | 0.005091 |
| ENSMUSG00000080059 |  | Rps19-ps3 | 191.7582697 | 92.568723 | 1.033576624 | 0.000214 |
| ENSMUSG00000058183 |  | Mmel1 | 417.5623113 | 90.60028279 | 2.198616159 | 0.010225 |
| ENSMUSG00000029059 |  | Fam213b | 2855.339034 | 835.4769154 | 1.772570973 | 0.001048 |
| ENSMUSG00000023153 |  | Tmem52 | 42.33076577 | 149.389709 | -1.817292746 | 0.020997 |
| ENSMUSG00000005907 |  | Pex1 | 1818.84269 | 896.9664633 | 1.019457072 | 0.007073 |
| ENSMUSG00000044968 |  | Napepld | 3348.649037 | 1557.569923 | 1.103673227 | 0.002448 |
| ENSMUSG00000105422 |  | Gm6089 | 12.6927063 | 1.674183108 | 2.739915763 | 0.023494 |
| ENSMUSG00000029168 |  | Dpysl5 | 447.5761908 | 71.01956669 | 2.657242873 | 0.032201 |
| ENSMUSG00000029108 |  | Pcdh7 | 656.3769529 | 216.2878398 | 1.60254259 | 0.003979 |
| ENSMUSG00000070780 |  | Rbm47 | 7748.84053 | 3720.173793 | 1.057974531 | 0.000868 |
| ENSMUSG00000035505 |  | Cox18 | 827.1354346 | 377.4620407 | 1.130771197 | 1.72E-05 |
| ENSMUSG00000029335 |  | Bmp3 | 218.4593424 | 32.57403641 | 2.743930767 | 0.011592 |
| ENSMUSG00000029299 |  | Abcg3 | 83.23471618 | 249.2882841 | -1.58279736 | 0.002591 |
| ENSMUSG00000034438 |  | Gbp8 | 21.33570648 | 63.92694603 | -1.581571215 | 0.001689 |
| ENSMUSG00000086847 |  | Tbx3os2 | 21.85852497 | 64.21065727 | -1.54990485 | 0.016311 |
| ENSMUSG00000032754 |  | Slc8b1 | 1007.81075 | 374.1791255 | 1.43220726 | 5.13E-09 |
| ENSMUSG00000029454 |  | Mapkapk5 | 1306.286956 | 526.9162537 | 1.307481788 | 0.006312 |
| ENSMUSG00000037411 |  | Serpine1 | 384.4601865 | 787.8681205 | -1.034352348 | 0.037114 |
| ENSMUSG00000066682 |  | Pilrb2 | 12.27618306 | 33.31803597 | -1.455917917 | 0.01509 |
| ENSMUSG00000046245 |  | Pilra | 36.3288622 | 107.7518618 | -1.561341813 | 4.18E-05 |
| ENSMUSG00000048988 |  | Elfn1 | 1283.286162 | 28.4718612 | 5.479321629 | 1.18E-16 |
| ENSMUSG00000029752 |  | Asns | 2863.414427 | 969.7730396 | 1.561649217 | 2.42E-06 |
| ENSMUSG00000029695 |  | Aass | 543.5525961 | 84.91512861 | 2.668748509 | 0.00029 |
| ENSMUSG00000029772 |  | Ahcyl2 | 11787.52277 | 5539.819384 | 1.08906731 | 0.000511 |
| ENSMUSG00000045613 |  | Chrm2 | 228.1446098 | 16.29062581 | 3.819118455 | 0.035372 |
| ENSMUSG00000038641 |  | Akr1d1 | 65304.81549 | 619.4333834 | 6.719339923 | 3.29E-06 |
| ENSMUSG00000068587 |  | Mgam | 109.6134539 | 22.16043432 | 2.330412599 | 0.015962 |
| ENSMUSG00000029864 |  | Gstk1 | 5309.211253 | 2058.50884 | 1.366275997 | 0.001663 |
| ENSMUSG00000051499 |  | Zfp786 | 133.7206757 | 65.73606992 | 1.017576684 | 0.047793 |
| ENSMUSG00000029816 |  | Gpnmb | 55.0765744 | 281.2770869 | -2.353183795 | 0.017835 |
| ENSMUSG00000029822 |  | Osbpl3 | 2710.034307 | 5580.584253 | -1.041919446 | 0.000182 |
| ENSMUSG00000038058 |  | Nod1 | 718.9126608 | 308.5896168 | 1.222323399 | 0.001741 |
| ENSMUSG00000029919 |  | Hpgds | 71.30401997 | 213.5722418 | -1.586056583 | 0.000768 |
| ENSMUSG00000036390 |  | Gadd45a | 805.4715956 | 293.2452118 | 1.4553503 | 0.003088 |
| ENSMUSG00000056091 |  | St3gal5 | 6467.157918 | 16341.56445 | -1.337349192 | 9.09E-06 |
| ENSMUSG00000009145 |  | Dqx1 | 47.56485257 | 109.804706 | -1.204758305 | 0.000312 |
| ENSMUSG00000063415 |  | Cyp26b1 | 392.451215 | 72.62471872 | 2.431702912 | 0.007308 |
| ENSMUSG00000079494 |  | Nat8f5 | 50.00429254 | 133.5765686 | -1.415241217 | 0.010605 |
| ENSMUSG00000030000 |  | Add2 | 981.517285 | 350.299598 | 1.487099496 | 0.022622 |
| ENSMUSG00000033152 |  | Podxl2 | 737.6749484 | 1661.246065 | -1.169847044 | 1.18E-16 |
| ENSMUSG00000030087 |  | Klf15 | 1638.165038 | 802.0013509 | 1.030450009 | 0.033636 |
| ENSMUSG00000030089 |  | Slc41a3 | 1367.377153 | 3889.363593 | -1.508275457 | 2.74E-06 |
| ENSMUSG00000035158 |  | Mitf | 873.7268754 | 373.5310783 | 1.22220794 | 1.41E-09 |
| ENSMUSG00000030077 |  | Chl1 | 8394.598149 | 1185.554136 | 2.823675514 | 0.00015 |
| ENSMUSG00000034648 |  | Lrrn1 | 6738.857113 | 2484.540832 | 1.439149411 | 0.031967 |
| ENSMUSG00000049112 |  | Oxtr | 21694.08105 | 3016.806424 | 2.845910992 | 0.001531 |
| ENSMUSG00000030257 |  | Srgap3 | 1282.678005 | 2714.121754 | -1.081089772 | 0.003733 |
| ENSMUSG00000030111 |  | A2m | 6580.81145 | 279.264162 | 4.555300446 | 4.62E-08 |
| ENSMUSG00000040552 |  | C3ar1 | 294.0257949 | 611.3113902 | -1.056714173 | 0.023705 |
| ENSMUSG00000043832 |  | Clec4a3 | 69.4501099 | 148.9244215 | -1.100873118 | 0.031405 |
| ENSMUSG00000023349 |  | Clec4n | 67.14914184 | 180.5830798 | -1.427400173 | 0.002238 |
| ENSMUSG00000098470 |  | C1rb | 33.25951806 | 73.80567206 | -1.155471671 | 0.009607 |
| ENSMUSG00000030351 |  | Tspan11 | 376.1622312 | 2020.852272 | -2.425560219 | 0.012289 |
| ENSMUSG00000079293 |  | Clec7a | 112.3110974 | 321.2392947 | -1.515436666 | 0.002223 |
| ENSMUSG00000090698 |  | Apold1 | 672.7232195 | 2480.158863 | -1.881898585 | 0.000269 |
| ENSMUSG00000008540 |  | Mgst1 | 45913.05201 | 12901.75056 | 1.831252116 | 0.000138 |
| ENSMUSG00000030228 |  | Pik3c2g | 12104.53048 | 329.5575656 | 5.197981931 | 0.027965 |
| ENSMUSG00000107956 |  | Speer9-ps1 | 50.29827364 | 9.926472903 | 2.309043572 | 0.000264 |
| ENSMUSG00000055541 |  | Lair1 | 118.9543124 | 292.0712008 | -1.293054298 | 0.005648 |
| ENSMUSG00000070873 |  | Lilra5 | 77.53543579 | 177.0155813 | -1.182199921 | 0.003186 |
| ENSMUSG00000030433 |  | Sbk2 | 0 | 4.98831075 | -18.3599827 | 8.39E-08 |
| ENSMUSG00000003545 |  | Fosb | 128.4596097 | 735.7331018 | -2.51657194 | 0.001334 |
| ENSMUSG00000074336 |  | Apoc4 | 356.8668255 | 175.4107842 | 1.025607601 | 0.048727 |
| ENSMUSG00000057454 |  | Lypd3 | 59.46965216 | 2.461862476 | 4.441899189 | 1.41E-05 |
| ENSMUSG00000074272 |  | Ceacam1 | 1160.066915 | 435.2150516 | 1.413255471 | 0.0345 |
| ENSMUSG00000052974 |  | Cyp2f2 | 12488.25178 | 309.9921952 | 5.329672434 | 2.27E-07 |
| ENSMUSG00000086868 |  | Gm15883 | 29.92936143 | 3.836968223 | 2.958925102 | 0.000259 |
| ENSMUSG00000030579 |  | Tyrobp | 210.5490065 | 455.0670617 | -1.113522596 | 0.001502 |
| ENSMUSG00000064109 |  | Hcst | 0 | 9.279632396 | -5.203736888 | 0.035531 |
| ENSMUSG00000036931 |  | Nfkbid | 35.36186641 | 94.98044011 | -1.41039532 | 0.003646 |
| ENSMUSG00000030577 |  | Cd22 | 12.71059505 | 41.84329866 | -1.715980938 | 0.037602 |
| ENSMUSG00000005553 |  | Atp4a | 38.06331237 | 115.3715826 | -1.597957586 | 0.000237 |
| ENSMUSG00000036578 |  | Fxyd7 | 125.7564765 | 10.3280835 | 3.616525361 | 0.016238 |
| ENSMUSG00000050440 |  | Hamp | 477.5965972 | 0.682183697 | 9.895187193 | 1.28E-11 |
| ENSMUSG00000003271 |  | Sult2b1 | 39.5894228 | 86.78384557 | -1.127023706 | 0.01045 |
| ENSMUSG00000040212 |  | Emp3 | 2036.286411 | 4149.567503 | -1.027075423 | 4.91E-05 |
| ENSMUSG00000040189 |  | Ccdc114 | 392.8833149 | 955.9445953 | -1.282330889 | 0.001911 |
| ENSMUSG00000030549 |  | Rhcg | 13.18406832 | 214.307461 | -4.022975126 | 5.84E-08 |
| ENSMUSG00000030606 |  | Hapln3 | 15.35817322 | 36.80261329 | -1.232133004 | 0.043167 |
| ENSMUSG00000030607 |  | Acan | 39.58706075 | 743.8452579 | -4.231743313 | 0.000181 |
| ENSMUSG00000046027 |  | Stard5 | 2485.26733 | 959.3191714 | 1.372046276 | 0.0253 |
| ENSMUSG00000108825 |  | Gm45838 | 150.6407368 | 40.22909039 | 1.884997865 | 0.00305 |
| ENSMUSG00000030562 |  | Nox4 | 889.6134362 | 191.2302781 | 2.213538632 | 0.001053 |
| ENSMUSG00000039428 |  | Tmem135 | 7305.653863 | 3561.789652 | 1.036070877 | 0.000111 |
| ENSMUSG00000074006 |  | Omp | 297.1651927 | 26.16305554 | 3.483322197 | 0.014486 |
| ENSMUSG00000035211 |  | Xrra1 | 91.90116495 | 15.37377485 | 2.571710332 | 0.012145 |
| ENSMUSG00000030729 |  | Pgm2l1 | 1920.178811 | 747.6769543 | 1.361163296 | 0.001648 |
| ENSMUSG00000030688 |  | Stard10 | 538.7041964 | 1488.599469 | -1.465780147 | 8E-05 |
| ENSMUSG00000001827 |  | Folr1 | 79.86745725 | 12.82751519 | 2.584450532 | 0.007144 |
| ENSMUSG00000078616 |  | Trim30c | 0 | 9.341816461 | -5.191984463 | 0.008613 |
| ENSMUSG00000034825 |  | Nrip3 | 347.1447775 | 96.08958243 | 1.853416591 | 0.018171 |
| ENSMUSG00000030771 |  | Micalcl | 0.447979707 | 33.82790216 | -6.116463508 | 0.004162 |
| ENSMUSG00000038244 |  | Mical2 | 901.0305264 | 2330.897558 | -1.370904116 | 8.24E-05 |
| ENSMUSG00000030905 |  | Crym | 21.86592823 | 270.6226346 | -3.630992602 | 3.63E-11 |
| ENSMUSG00000052889 |  | Prkcb | 530.4071407 | 1149.287376 | -1.115469575 | 0.012497 |
| ENSMUSG00000042978 |  | Sbk1 | 3846.152069 | 929.9418441 | 2.048220636 | 5.67E-12 |
| ENSMUSG00000049350 |  | Zg16 | 0.907885251 | 0 | 26.43407766 | 0.000479 |
| ENSMUSG00000049091 |  | Sephs2 | 4509.630451 | 2169.286892 | 1.055725935 | 0.023962 |
| ENSMUSG00000108815 |  | AC149222.1 | 1382.55476 | 0 | 13.35295714 | 0.000644 |
| ENSMUSG00000030844 |  | Rgs10 | 452.3077959 | 221.5589665 | 1.024685449 | 0.006611 |
| ENSMUSG00000030956 |  | Fam53b | 5514.811118 | 2146.760227 | 1.36125728 | 1.07E-08 |
| ENSMUSG00000030861 |  | Acadsb | 21004.42212 | 8340.066628 | 1.332393739 | 3.9E-05 |
| ENSMUSG00000078566 |  | Bnip3 | 9829.660702 | 4087.487376 | 1.265987596 | 0.002331 |
| ENSMUSG00000025473 |  | Adam8 | 97.75579369 | 272.7348664 | -1.483228577 | 0.049965 |
| ENSMUSG00000025464 |  | Paox | 1069.322632 | 467.735237 | 1.19268352 | 0.000248 |
| ENSMUSG00000060314 |  | Zfp941 | 280.544618 | 589.4856622 | -1.070639559 | 0.020604 |
| ENSMUSG00000038618 |  | Rassf7 | 88.55876539 | 31.81726498 | 1.471078447 | 0.031751 |
| ENSMUSG00000025496 |  | Drd4 | 9.51510792 | 225.739989 | -4.569611366 | 1.98E-13 |
| ENSMUSG00000037887 |  | Dusp8 | 583.6275992 | 1529.078492 | -1.38885041 | 0.005306 |
| ENSMUSG00000009545 |  | Kcnq1 | 277.2398144 | 795.657093 | -1.521300891 | 0.00501 |
| ENSMUSG00000037664 |  | Cdkn1c | 11189.33235 | 4995.151205 | 1.163251525 | 0.025705 |
| ENSMUSG00000109061 |  | Map2k7 | 0 | 87.70658814 | -36.41865131 | 3.23E-10 |
| ENSMUSG00000031453 |  | Rasa3 | 3812.402501 | 1509.800684 | 1.336035214 | 0.000685 |
| ENSMUSG00000037738 |  | Nek5 | 404.5478699 | 1526.636077 | -1.916104662 | 3.42E-09 |
| ENSMUSG00000031545 |  | Gpat4 | 9132.572846 | 4542.19118 | 1.007672068 | 0.014745 |
| ENSMUSG00000037406 |  | Htra4 | 534.589598 | 246.5579877 | 1.115697309 | 0.024219 |
| ENSMUSG00000031488 |  | Rab11fip1 | 361.0082219 | 1301.093594 | -1.849076008 | 0.000339 |
| ENSMUSG00000039633 |  | Lonrf1 | 597.3591485 | 1249.889778 | -1.064020878 | 0.001337 |
| ENSMUSG00000031520 |  | Vegfc | 827.0035145 | 222.1621003 | 1.894910904 | 6.79E-07 |
| ENSMUSG00000053886 |  | Sh2d4a | 515.7301509 | 82.86802682 | 2.62359392 | 2.69E-09 |
| ENSMUSG00000005413 |  | Hmox1 | 1154.75793 | 5346.059726 | -2.210845235 | 1.48E-18 |
| ENSMUSG00000031618 |  | Nr3c2 | 537.1878106 | 1075.022822 | -1.000004761 | 0.01296 |
| ENSMUSG00000031700 |  | Gpt2 | 11566.08154 | 2900.836199 | 1.995085082 | 0.019117 |
| ENSMUSG00000031654 |  | Cbln1 | 144.1958948 | 9.299994473 | 3.885311073 | 0.007691 |
| ENSMUSG00000045333 |  | Zfp423 | 559.8120871 | 1126.832993 | -1.008828447 | 0.008757 |
| ENSMUSG00000031659 |  | Adcy7 | 707.7461916 | 1800.714429 | -1.346895246 | 2.16E-10 |
| ENSMUSG00000054400 |  | Cklf | 1573.20482 | 3189.775677 | -1.020463779 | 0.00014 |
| ENSMUSG00000031875 |  | Cmtm3 | 2362.359034 | 4968.322951 | -1.072342835 | 2.11E-06 |
| ENSMUSG00000059854 |  | Hydin | 56.72378661 | 24.42557697 | 1.211951757 | 0.030201 |
| ENSMUSG00000031750 |  | Il34 | 915.032622 | 348.0838107 | 1.396015401 | 0.002981 |
| ENSMUSG00000033579 |  | Fa2h | 510.2000584 | 3.268046099 | 7.244721663 | 1.09E-07 |
| ENSMUSG00000031767 |  | Nudt7 | 1928.07986 | 693.9009659 | 1.474806909 | 0.005524 |
| ENSMUSG00000031853 |  | Map3k21 | 176.5899882 | 70.51691248 | 1.303657592 | 0.003308 |
| ENSMUSG00000033998 |  | Kcnk1 | 168.3369101 | 657.237168 | -1.964955878 | 0.012385 |
| ENSMUSG00000057060 |  | Slc35f3 | 97.74830445 | 18.67642773 | 2.374023962 | 0.026125 |
| ENSMUSG00000062380 |  | Tubb3 | 1735.556507 | 235.2652427 | 2.883277855 | 0.016827 |
| ENSMUSG00000042812 |  | Foxf1 | 25.83935522 | 55.90117938 | -1.110436905 | 0.023757 |
| ENSMUSG00000070323 |  | Mmp27 | 39.49413167 | 2.231998674 | 3.92444382 | 0.009661 |
| ENSMUSG00000049723 |  | Mmp12 | 114.076601 | 331.1124876 | -1.538140138 | 0.029422 |
| ENSMUSG00000031936 |  | Hephl1 | 522.0373864 | 27.4515581 | 4.240151076 | 8.24E-05 |
| ENSMUSG00000004098 |  | Col5a3 | 12878.41135 | 3819.836079 | 1.753151835 | 0.004742 |
| ENSMUSG00000040146 |  | Rgl3 | 1117.690733 | 523.6705461 | 1.092954374 | 0.000702 |
| ENSMUSG00000043067 |  | Dpy19l1 | 4810.960225 | 2080.121519 | 1.209179449 | 8.24E-05 |
| ENSMUSG00000036611 |  | Eepd1 | 3467.566314 | 1488.477415 | 1.219448384 | 0.019145 |
| ENSMUSG00000031995 |  | St14 | 54.82361355 | 18.42415831 | 1.579584306 | 0.022622 |
| ENSMUSG00000046240 |  | Hepacam | 61.77457886 | 12.6840821 | 2.276730906 | 0.017759 |
| ENSMUSG00000034739 |  | Mfrp | 6.396418549 | 37.62952719 | -2.563206284 | 0.034626 |
| ENSMUSG00000111329 |  | AC122273.1 | 60.83240174 | 12.56414922 | 2.294612976 | 3.29E-06 |
| ENSMUSG00000032269 |  | Htr3a | 1042.500341 | 195.5456371 | 2.413673736 | 0.004432 |
| ENSMUSG00000008590 |  | Htr3b | 289.3366255 | 37.7444641 | 2.938337038 | 0.036507 |
| ENSMUSG00000042195 |  | Slc35f2 | 56.68348653 | 149.5283687 | -1.394808698 | 0.036473 |
| ENSMUSG00000032281 |  | Acsbg1 | 7817.4391 | 19490.61388 | -1.318047645 | 0.00152 |
| ENSMUSG00000051243 |  | Islr2 | 209.3217418 | 19.10430848 | 3.454832943 | 0.005311 |
| ENSMUSG00000032238 |  | Rora | 13187.95694 | 4907.643461 | 1.425859013 | 1.01E-08 |
| ENSMUSG00000043013 |  | Onecut1 | 0 | 14.93495323 | -5.899666485 | 0.001052 |
| ENSMUSG00000032360 |  | Hcrtr2 | 34.4747506 | 5.513184858 | 2.642412337 | 0.006571 |
| ENSMUSG00000057933 |  | Gsta2 | 10.002466 | 0 | 6.241997833 | 0.042176 |
| ENSMUSG00000042761 |  | Mrap2 | 84.3739414 | 11.23068976 | 2.905410112 | 0.044733 |
| ENSMUSG00000032417 |  | Rwdd2a | 247.5616859 | 101.158389 | 1.300245677 | 0.006073 |
| ENSMUSG00000033491 |  | Prss35 | 20446.2612 | 53297.88428 | -1.382261868 | 0.000395 |
| ENSMUSG00000049493 |  | Pls1 | 61.20810626 | 18.36457849 | 1.716994733 | 0.036374 |
| ENSMUSG00000032537 |  | Ephb1 | 145.364794 | 338.1989909 | -1.218107902 | 0.002704 |
| ENSMUSG00000032531 |  | Amotl2 | 11994.74926 | 4321.791024 | 1.472382951 | 6.53E-07 |
| ENSMUSG00000025648 |  | Pfkfb4 | 327.6647746 | 937.1253841 | -1.5154792 | 0.002199 |
| ENSMUSG00000025644 |  | Gm7628 | 163.7566495 | 71.1568992 | 1.201269697 | 0.00644 |
| ENSMUSG00000033392 |  | Clasp2 | 10346.73957 | 22559.57827 | -1.124634437 | 7.08E-05 |
| ENSMUSG00000041608 |  | Entpd3 | 531.0762171 | 135.8709907 | 1.965197493 | 0.005258 |
| ENSMUSG00000032528 |  | Vipr1 | 21.8203854 | 59.58323974 | -1.448270116 | 0.024653 |
| ENSMUSG00000040653 |  | Ppp1r14c | 704.2010981 | 205.4564225 | 1.772633299 | 4.66E-07 |
| ENSMUSG00000060487 |  | Samd5 | 2312.985321 | 729.5783563 | 1.662837676 | 1.11E-09 |
| ENSMUSG00000019850 |  | Tnfaip3 | 355.1324613 | 794.1931745 | -1.160056005 | 0.001197 |
| ENSMUSG00000020010 |  | Vnn3 | 3937.056139 | 805.5312268 | 2.288101278 | 0.000342 |
| ENSMUSG00000037440 |  | Vnn1 | 2380.217281 | 594.6916195 | 1.999094541 | 0.00017 |
| ENSMUSG00000049872 |  | Fam26e | 60.64498769 | 27.35456475 | 1.144092916 | 0.047808 |
| ENSMUSG00000071335 |  | Mfsd4b3 | 130.3593094 | 37.45205804 | 1.800838525 | 6.23E-06 |
| ENSMUSG00000111171 |  | AC112265.1 | 71.6977288 | 14.65660615 | 2.233398679 | 0.016264 |
| ENSMUSG00000003746 |  | Man1a | 11759.1797 | 2968.955002 | 1.985476097 | 3.21E-05 |
| ENSMUSG00000050953 |  | Gja1 | 13131.59725 | 3276.957497 | 2.00229342 | 4.16E-08 |
| ENSMUSG00000020085 |  | Aifm2 | 10149.27676 | 5024.736026 | 1.013839068 | 0.000237 |
| ENSMUSG00000020262 |  | Adarb1 | 2280.540804 | 5512.619783 | -1.273342301 | 0.004906 |
| ENSMUSG00000020325 |  | Fstl3 | 347.3793264 | 1244.847723 | -1.841489425 | 1.17E-11 |
| ENSMUSG00000113262 |  | Dohh | 49.2952988 | 0 | 8.543348024 | 0.00028 |
| ENSMUSG00000034758 |  | Tle6 | 273.106154 | 729.285031 | -1.416899592 | 0.029972 |
| ENSMUSG00000020263 |  | Appl2 | 22925.29281 | 7045.064635 | 1.70215904 | 0.006214 |
| ENSMUSG00000047638 |  | Nr1h4 | 1404.875791 | 642.526112 | 1.127850257 | 0.020897 |
| ENSMUSG00000020027 |  | Socs2 | 1850.360966 | 905.1597827 | 1.029746765 | 5.68E-06 |
| ENSMUSG00000056888 |  | Glipr1 | 30.30650629 | 69.59097169 | -1.199102232 | 0.016726 |
| ENSMUSG00000112470 |  | AC158804.1 | 1.815770502 | 19.48057782 | -3.434608409 | 0.032148 |
| ENSMUSG00000052302 |  | Tbc1d30 | 306.6609013 | 106.2947606 | 1.531550891 | 0.000136 |
| ENSMUSG00000025432 |  | Avil | 502.7790238 | 34.02169637 | 3.884148915 | 0.000555 |
| ENSMUSG00000025401 |  | Myo1a | 53.60910118 | 869.1548911 | -4.020693564 | 1.16E-21 |
| ENSMUSG00000047631 |  | Apof | 339.202557 | 102.2707983 | 1.737918078 | 0.007444 |
| ENSMUSG00000020435 |  | Osbp2 | 137.4168871 | 298.058773 | -1.108720108 | 0.000128 |
| ENSMUSG00000009073 |  | Nf2 | 13314.26235 | 5029.872073 | 1.404290997 | 8.16E-05 |
| ENSMUSG00000048834 |  | Vstm2a | 64.50208231 | 11.4638842 | 2.463595184 | 0.011064 |
| ENSMUSG00000086468 |  | Etaa1os | 39.38319828 | 16.37557369 | 1.245991914 | 0.037195 |
| ENSMUSG00000044072 |  | Eml6 | 547.7566179 | 1337.153202 | -1.287494197 | 0.038484 |
| ENSMUSG00000018387 |  | Shroom1 | 224.2941031 | 561.0586055 | -1.322120262 | 0.044028 |
| ENSMUSG00000018238 |  | Gdf9 | 47.6873947 | 134.4662641 | -1.499494614 | 0.032789 |
| ENSMUSG00000020334 |  | Slc22a4 | 305.1169021 | 101.1574401 | 1.596418106 | 0.015965 |
| ENSMUSG00000020524 |  | Gria1 | 345.3481346 | 46.25752563 | 2.900329202 | 0.007308 |
| ENSMUSG00000050818 |  | Olfr330 | 11.80415015 | 1.145241945 | 3.285252835 | 0.039537 |
| ENSMUSG00000032691 |  | Nlrp3 | 25.85508346 | 53.88628264 | -1.024485558 | 0.019644 |
| ENSMUSG00000020534 |  | Shmt1 | 2474.494134 | 933.345946 | 1.405697527 | 8.25E-05 |
| ENSMUSG00000019102 |  | Aldh3a1 | 24.95748216 | 1.6231056 | 4.10733211 | 0.015621 |
| ENSMUSG00000046417 |  | Lrrc75a | 1399.124871 | 691.8926484 | 1.016108426 | 0.035171 |
| ENSMUSG00000042826 |  | Fgf11 | 202.3642699 | 566.7475205 | -1.48203559 | 1.76E-08 |
| ENSMUSG00000000317 |  | Bcl6b | 1140.337649 | 3413.906772 | -1.581958101 | 3.2E-08 |
| ENSMUSG00000018920 |  | Cxcl16 | 310.828124 | 637.3470347 | -1.036048185 | 0.001484 |
| ENSMUSG00000040471 |  | Ggt6 | 61.6990652 | 4.440865332 | 3.843626791 | 0.011154 |
| ENSMUSG00000043029 |  | Trpv3 | 31.36150551 | 74.17398301 | -1.239042312 | 0.04042 |
| ENSMUSG00000005951 |  | Shpk | 462.6587561 | 229.8379246 | 1.007248865 | 6.65E-05 |
| ENSMUSG00000017453 |  | Pipox | 274.9833361 | 125.0298192 | 1.137043361 | 0.003235 |
| ENSMUSG00000020826 |  | Nos2 | 158.0379697 | 514.0342632 | -1.701928669 | 8.73E-08 |
| ENSMUSG00000018986 |  | Slfn3 | 225.6999632 | 478.2809863 | -1.081715148 | 0.00017 |
| ENSMUSG00000018648 |  | Dusp14 | 176.7374599 | 484.5723337 | -1.452720241 | 0.000203 |
| ENSMUSG00000020532 |  | Acaca | 18798.26975 | 5325.594325 | 1.819610691 | 0.038815 |
| ENSMUSG00000018427 |  | Ypel2 | 2702.658375 | 939.3330131 | 1.522898078 | 1.52E-07 |
| ENSMUSG00000020865 |  | Abcc3 | 173.5065577 | 549.5211484 | -1.666397853 | 4.87E-07 |
| ENSMUSG00000018411 |  | Mapt | 7114.303497 | 2929.269147 | 1.279927213 | 0.019167 |
| ENSMUSG00000034652 |  | Cd300a | 106.6167427 | 244.4328774 | -1.200758723 | 0.021805 |
| ENSMUSG00000044811 |  | Cd300c2 | 68.97735681 | 154.9545589 | -1.169832654 | 0.005726 |
| ENSMUSG00000020734 |  | Grin2c | 88.98629275 | 216.1189198 | -1.277914482 | 0.00023 |
| ENSMUSG00000020814 |  | Mxra7 | 1294.87025 | 2868.902558 | -1.147404235 | 1.57E-14 |
| ENSMUSG00000033987 |  | Dnah17 | 0 | 13.02350862 | -5.698654462 | 0.023303 |
| ENSMUSG00000033857 |  | Engase | 390.7546059 | 182.8548937 | 1.089871064 | 9.09E-06 |
| ENSMUSG00000000056 |  | Narf | 1249.893738 | 547.7414728 | 1.186693241 | 1.45E-07 |
| ENSMUSG00000039208 |  | Metrnl | 532.225717 | 233.3195663 | 1.18873948 | 0.03469 |
| ENSMUSG00000113536 |  | CT030170.4 | 47.59466716 | 22.36125803 | 1.117754949 | 0.029868 |
| ENSMUSG00000036136 |  | Fam110c | 39.8707579 | 3.038550503 | 3.721113456 | 0.041318 |
| ENSMUSG00000020572 |  | Nampt | 17751.92298 | 5807.317336 | 1.611790474 | 6.68E-08 |
| ENSMUSG00000096954 |  | Gdap10 | 479.4081687 | 220.1916198 | 1.122934377 | 0.04101 |
| ENSMUSG00000048285 |  | Frmd6 | 3827.244974 | 10417.11883 | -1.444535223 | 4.91E-08 |
| ENSMUSG00000021228 |  | Acot3 | 42.56151684 | 0 | 8.33151049 | 3.87E-06 |
| ENSMUSG00000052392 |  | Acot4 | 377.8765554 | 60.51657145 | 2.652917927 | 8.74E-05 |
| ENSMUSG00000021036 |  | Sptlc2 | 16399.43098 | 8179.460487 | 1.003522622 | 1.01E-08 |
| ENSMUSG00000021886 |  | Gpr65 | 135.5104793 | 313.3664291 | -1.210015916 | 0.001678 |
| ENSMUSG00000033854 |  | Kcnk10 | 96.88447639 | 5.825034098 | 4.041949739 | 0.007691 |
| ENSMUSG00000057963 |  | Itpk1 | 1713.617239 | 3567.155763 | -1.057402938 | 1.76E-08 |
| ENSMUSG00000060807 |  | Serpina6 | 23.75345529 | 144.4458212 | -2.605626477 | 0.020917 |
| ENSMUSG00000041323 |  | Ak7 | 8.119663053 | 26.09273058 | -1.663960005 | 0.040991 |
| ENSMUSG00000076617 |  | Ighm | 579.5949415 | 2073.262063 | -1.838600415 | 1.06E-06 |
| ENSMUSG00000102332 |  | Gm19331 | 10.87621562 | 0 | 6.363134744 | 0.008022 |
| ENSMUSG00000021213 |  | Akr1c13 | 2193.551227 | 971.9705358 | 1.17276464 | 0.019131 |
| ENSMUSG00000046159 |  | Chrm3 | 197.3399336 | 440.7974477 | -1.157216547 | 0.001902 |
| ENSMUSG00000055137 |  | Sugct | 455.412802 | 203.168506 | 1.1623375 | 0.003455 |
| ENSMUSG00000044734 |  | Serpinb1a | 9053.339957 | 1268.132762 | 2.835026617 | 4.69E-06 |
| ENSMUSG00000045136 |  | Tubb2b | 750.135296 | 92.83650743 | 3.015458675 | 0.027737 |
| ENSMUSG00000039109 |  | F13a1 | 471.0400674 | 129.3126285 | 1.862646075 | 0.001399 |
| ENSMUSG00000054889 |  | Dsp | 184.7498357 | 47.46308381 | 1.96739714 | 0.028694 |
| ENSMUSG00000021384 |  | Susd3 | 16.33853519 | 215.9181949 | -3.723627631 | 0.000227 |
| ENSMUSG00000025877 |  | Hk3 | 23.58753085 | 71.22480563 | -1.597323218 | 0.039306 |
| ENSMUSG00000052957 |  | Gas1 | 1813.390667 | 737.4323898 | 1.2981761 | 0.037642 |
| ENSMUSG00000021477 |  | Ctsl | 24244.08247 | 9450.056997 | 1.359187784 | 0.017545 |
| ENSMUSG00000001504 |  | Irx2 | 10.40726493 | 0.495999705 | 3.928551442 | 0.045136 |
| ENSMUSG00000114554 |  | CT009711.2 | 16.32352713 | 0 | 6.948859695 | 0.00032 |
| ENSMUSG00000021620 |  | Acot12 | 38.20806429 | 151.0565212 | -1.982902591 | 0.000113 |
| ENSMUSG00000021684 |  | Pde8b | 4592.950076 | 2031.945752 | 1.175850436 | 0.005278 |
| ENSMUSG00000091387 |  | Gcnt4 | 66.15901454 | 20.42760031 | 1.680381826 | 0.004588 |
| ENSMUSG00000041817 |  | Fam169a | 637.44569 | 146.0169941 | 2.123132441 | 0.001077 |
| ENSMUSG00000071203 |  | Naip5 | 2260.983326 | 523.0435153 | 2.111265922 | 0.017192 |
| ENSMUSG00000021638 |  | Ocln | 440.7126104 | 170.860808 | 1.363781546 | 0.006073 |
| ENSMUSG00000021636 |  | Marveld2 | 40.85636262 | 105.1769302 | -1.364960846 | 0.005293 |
| ENSMUSG00000041417 |  | Pik3r1 | 13969.19131 | 6239.143542 | 1.162787136 | 0.030329 |
| ENSMUSG00000021624 |  | Cd180 | 51.87136725 | 126.5698348 | -1.28893702 | 0.029343 |
| ENSMUSG00000042743 |  | Sgtb | 1250.024959 | 3314.462206 | -1.406882581 | 0.000132 |
| ENSMUSG00000055194 |  | Actbl2 | 0.895959415 | 19.06896529 | -4.404667098 | 0.042447 |
| ENSMUSG00000021701 |  | Plk2 | 1595.783735 | 3811.679202 | -1.256015475 | 0.026643 |
| ENSMUSG00000021758 |  | Ddx4 | 91.73060238 | 40.8622301 | 1.144189414 | 0.04188 |
| ENSMUSG00000021728 |  | Emb | 10149.77454 | 2278.907309 | 2.154746212 | 0.021533 |
| ENSMUSG00000094634 |  | Gm3468 | 11.74452097 | 29.68743133 | -1.306613227 | 0.022614 |
| ENSMUSG00000021779 |  | Thrb | 5446.703618 | 2132.524928 | 1.352582977 | 0.03023 |
| ENSMUSG00000114553 |  | AC154646.1 | 28.61071432 | 3.131274293 | 3.089963205 | 0.040287 |
| ENSMUSG00000051506 |  | Wdfy4 | 159.3473701 | 321.4780658 | -1.011565481 | 0.027981 |
| ENSMUSG00000023064 |  | Sncg | 1218.206744 | 167.0958065 | 2.866407459 | 0.013731 |
| ENSMUSG00000050335 |  | Lgals3 | 224.7229552 | 583.7972869 | -1.379249933 | 0.020949 |
| ENSMUSG00000046352 |  | Gjb2 | 938.7003546 | 118.673824 | 2.977782799 | 0.001572 |
| ENSMUSG00000021944 |  | Gata4 | 92.07064496 | 38.03281312 | 1.285830112 | 0.020153 |
| ENSMUSG00000033730 |  | Egr3 | 54.34129668 | 503.6221669 | -3.210538475 | 1.01E-05 |
| ENSMUSG00000034959 |  | Rubcnl | 28.95804459 | 83.12269119 | -1.505834229 | 0.000181 |
| ENSMUSG00000021998 |  | Lcp1 | 1377.811624 | 3025.361125 | -1.135019167 | 1.73E-05 |
| ENSMUSG00000022003 |  | Slc25a30 | 2610.882685 | 6532.508167 | -1.323205426 | 0.006253 |
| ENSMUSG00000048349 |  | Pou4f1 | 30.80218934 | 7.892693691 | 1.905341099 | 0.004838 |
| ENSMUSG00000045871 |  | Slitrk6 | 80.46655487 | 242.9334268 | -1.594234138 | 0.036262 |
| ENSMUSG00000022148 |  | Fyb | 246.1686031 | 536.800332 | -1.123899636 | 0.003151 |
| ENSMUSG00000005268 |  | Prlr | 30108.49424 | 10873.0505 | 1.469248783 | 0.000117 |
| ENSMUSG00000022330 |  | Osr2 | 1023.645721 | 487.645202 | 1.067586062 | 0.000883 |
| ENSMUSG00000022419 |  | Deptor | 26532.06977 | 9633.783856 | 1.461506939 | 1.99E-06 |
| ENSMUSG00000022376 |  | Adcy8 | 245.6196818 | 65.09053332 | 1.897777023 | 0.019365 |
| ENSMUSG00000013846 |  | St3gal1 | 11709.34932 | 3665.396786 | 1.675346717 | 0.000233 |
| ENSMUSG00000022602 |  | Arc | 78.49174456 | 383.2852835 | -2.285713554 | 0.00048 |
| ENSMUSG00000000934 |  | Top1mt | 1025.156808 | 340.4455204 | 1.589162356 | 7.04E-06 |
| ENSMUSG00000057346 |  | Apol9a | 53.64395698 | 152.3442405 | -1.500788704 | 0.004511 |
| ENSMUSG00000068246 |  | Apol9b | 62.25025794 | 186.3470176 | -1.577718865 | 0.0018 |
| ENSMUSG00000071713 |  | Csf2rb | 763.8209853 | 186.8986858 | 2.033190534 | 0.008432 |
| ENSMUSG00000016552 |  | Foxred2 | 254.5276593 | 533.0399863 | -1.064333853 | 0.014936 |
| ENSMUSG00000016942 |  | Tmprss6 | 99.42553279 | 35.92517257 | 1.477198516 | 0.006312 |
| ENSMUSG00000044216 |  | Kcnj4 | 4.951628455 | 77.5924749 | -3.952480066 | 1.24E-07 |
| ENSMUSG00000042351 |  | Grap2 | 14.99983882 | 33.89176156 | -1.147065277 | 0.038909 |
| ENSMUSG00000042109 |  | Csdc2 | 803.4355143 | 2018.828744 | -1.329562388 | 8.27E-05 |
| ENSMUSG00000061740 |  | Cyp2d22 | 1698.837022 | 806.5507964 | 1.075747007 | 0.000297 |
| ENSMUSG00000016758 |  | Bik | 52.64190384 | 112.6654191 | -1.095674075 | 0.005374 |
| ENSMUSG00000036273 |  | Lrrk2 | 11691.72163 | 3249.858477 | 1.846794571 | 0.000538 |
| ENSMUSG00000022479 |  | Vdr | 590.836023 | 1706.913632 | -1.530577333 | 0.028694 |
| ENSMUSG00000054855 |  | Rnd1 | 117.4999132 | 259.3917792 | -1.141817803 | 0.027204 |
| ENSMUSG00000023484 |  | Prph | 1292.165009 | 131.4762001 | 3.297847658 | 0.016187 |
| ENSMUSG00000023034 |  | Nr4a1 | 11905.729 | 26474.6253 | -1.152924035 | 0.043 |
| ENSMUSG00000023045 |  | Soat2 | 19.07483696 | 2.387642941 | 3.318268144 | 0.002302 |
| ENSMUSG00000054939 |  | Zfp174 | 972.3160576 | 276.2868127 | 1.812776453 | 4.07E-06 |
| ENSMUSG00000039457 |  | Ppl | 139.7263816 | 519.6980327 | -1.894164451 | 1.27E-05 |
| ENSMUSG00000008393 |  | Carhsp1 | 7192.796267 | 3484.346231 | 1.045500053 | 5.86E-06 |
| ENSMUSG00000022758 |  | P2rx6 | 21.75983451 | 75.82741581 | -1.799414431 | 0.010447 |
| ENSMUSG00000022756 |  | Slc7a4 | 275.2249351 | 703.7491603 | -1.353114502 | 1.42E-05 |
| ENSMUSG00000003526 |  | Prodh | 826.5483173 | 258.0821012 | 1.678195526 | 0.001052 |
| ENSMUSG00000041205 |  | Map6d1 | 283.7970145 | 35.39272506 | 2.986002662 | 2.76E-07 |
| ENSMUSG00000051146 |  | Camk2n2 | 518.6312899 | 1763.638092 | -1.766067094 | 2.53E-12 |
| ENSMUSG00000022871 |  | Fetub | 4416.044107 | 361.166406 | 3.610367612 | 5.52E-05 |
| ENSMUSG00000022802 |  | Lmln | 920.0672441 | 1854.627567 | -1.012137886 | 0.009812 |
| ENSMUSG00000052133 |  | Sema5b | 649.7652757 | 2696.036706 | -2.053022172 | 6.01E-10 |
| ENSMUSG00000047261 |  | Gap43 | 753.8212234 | 101.4937385 | 2.893739059 | 0.028076 |
| ENSMUSG00000055447 |  | Cd47 | 14663.72225 | 6942.796462 | 1.078731296 | 2.72E-07 |
| ENSMUSG00000022865 |  | Cxadr | 1759.195944 | 341.3230768 | 2.364769795 | 2.91E-05 |
| ENSMUSG00000022893 |  | Adamts1 | 3286.49663 | 11649.79499 | -1.825580429 | 3.49E-07 |
| ENSMUSG00000089774 |  | Slc5a3 | 3584.672367 | 7292.518402 | -1.024791859 | 0.000109 |
| ENSMUSG00000022948 |  | Setd4 | 206.5171438 | 94.20940394 | 1.130849491 | 0.002578 |
| ENSMUSG00000023800 |  | Tiam2 | 1915.984299 | 803.6227107 | 1.252860871 | 0.01687 |
| ENSMUSG00000023827 |  | Agpat4 | 906.8692864 | 2046.245561 | -1.173359846 | 0.000648 |
| ENSMUSG00000038347 |  | Tcte2 | 40.7182938 | 85.34858928 | -1.060526044 | 0.015533 |
| ENSMUSG00000024155 |  | Meiob | 37.22122076 | 93.51040134 | -1.326228691 | 0.037114 |
| ENSMUSG00000024300 |  | Myo1f | 120.0118752 | 288.4619007 | -1.268094588 | 0.007062 |
| ENSMUSG00000092471 |  | Cyp21a2-ps | 3136.706723 | 1379.650482 | 1.18458862 | 0.015828 |
| ENSMUSG00000024397 |  | Aif1 | 58.58129754 | 181.8388033 | -1.636216056 | 0.001972 |
| ENSMUSG00000096847 |  | Tmem151b | 155.4862965 | 4.402917897 | 5.157430593 | 0.019064 |
| ENSMUSG00000023979 |  | Guca1b | 77.49873657 | 32.96213872 | 1.199220833 | 0.0018 |
| ENSMUSG00000023992 |  | Trem2 | 60.82099454 | 158.6880549 | -1.38531117 | 0.021599 |
| ENSMUSG00000024164 |  | C3 | 73838.72611 | 2672.193834 | 4.788350373 | 0.000195 |
| ENSMUSG00000034116 |  | Vav1 | 91.26597273 | 197.7565205 | -1.112281949 | 0.001209 |
| ENSMUSG00000004730 |  | Adgre1 | 325.4825213 | 711.8923347 | -1.129824684 | 0.010447 |
| ENSMUSG00000050612 |  | Txndc2 | 787.8153803 | 332.3370455 | 1.242368456 | 0.04586 |
| ENSMUSG00000024064 |  | Galnt14 | 58.05343784 | 257.5343677 | -2.14866386 | 0.021928 |
| ENSMUSG00000038541 |  | Srd5a2 | 1280.358467 | 41983.18353 | -5.03519134 | 8.26E-33 |
| ENSMUSG00000059811 |  | Atl2 | 11407.61429 | 5666.310569 | 1.009414739 | 0.034022 |
| ENSMUSG00000024247 |  | Pkdcc | 3892.985543 | 910.2610922 | 2.096563465 | 2.04E-09 |
| ENSMUSG00000063889 |  | Crem | 4629.535472 | 9384.76147 | -1.019532835 | 0.004298 |
| ENSMUSG00000061013 |  | Mkx | 871.1423008 | 296.6626527 | 1.551938564 | 0.038933 |
| ENSMUSG00000042942 |  | Greb1l | 3458.0978 | 846.9840146 | 2.028610325 | 0.000457 |
| ENSMUSG00000033107 |  | Rnf125 | 531.5365054 | 150.3034018 | 1.823859144 | 0.010038 |
| ENSMUSG00000024277 |  | Mapre2 | 6081.056136 | 15129.56428 | -1.315177618 | 1.94E-23 |
| ENSMUSG00000024381 |  | Bin1 | 2130.852332 | 4341.114076 | -1.026857115 | 0.005003 |
| ENSMUSG00000042834 |  | Nrep | 40631.7225 | 3153.336677 | 3.687410719 | 1.05E-05 |
| ENSMUSG00000024486 |  | Hbegf | 199.1254865 | 515.3548216 | -1.368973466 | 8.27E-05 |
| ENSMUSG00000090264 |  | Eif4ebp3 | 28.19275074 | 7.698173078 | 1.886286389 | 0.020584 |
| ENSMUSG00000024427 |  | Spry4 | 1207.714036 | 3084.093044 | -1.351849964 | 6.77E-07 |
| ENSMUSG00000073565 |  | Prr16 | 1541.368877 | 168.0957947 | 3.191333373 | 5.34E-05 |
| ENSMUSG00000024600 |  | Slc27a6 | 264.5206318 | 30.87431956 | 3.104336249 | 1.98E-05 |
| ENSMUSG00000034320 |  | Slc26a2 | 2085.229733 | 835.3260261 | 1.319715265 | 0.000361 |
| ENSMUSG00000024575 |  | Pde6a | 46.81724594 | 0.743999558 | 5.565155606 | 0.000288 |
| ENSMUSG00000045730 |  | Adrb2 | 565.9982771 | 70.38378591 | 3.018419733 | 3.29E-06 |
| ENSMUSG00000046318 |  | Ccbe1 | 3678.897617 | 7441.797853 | -1.016448737 | 0.002136 |
| ENSMUSG00000038121 |  | Fam210a | 15078.5183 | 5764.280414 | 1.386773305 | 1.98E-10 |
| ENSMUSG00000044646 |  | Zbtb7c | 559.7608688 | 133.0504242 | 2.072164637 | 8.97E-05 |
| ENSMUSG00000024552 |  | Slc14a2 | 15.89487652 | 0 | 6.910298881 | 0.005082 |
| ENSMUSG00000073514 |  | Dok6 | 59.47777559 | 17.26541808 | 1.788534414 | 0.038549 |
| ENSMUSG00000024885 |  | Aldh3b1 | 3813.040165 | 1592.239772 | 1.259490381 | 0.008904 |
| ENSMUSG00000024912 |  | Fosl1 | 11.80054928 | 49.07806531 | -2.040980263 | 0.005646 |
| ENSMUSG00000024799 |  | Tm7sf2 | 772.2914278 | 318.7438648 | 1.275713259 | 0.034144 |
| ENSMUSG00000047787 |  | Flrt1 | 137.4011588 | 439.3041632 | -1.674759966 | 5.99E-06 |
| ENSMUSG00000035179 |  | Ppp1r32 | 0 | 6.821468769 | -4.745920598 | 0.044861 |
| ENSMUSG00000024672 |  | Ms4a7 | 90.29209231 | 269.2770428 | -1.577256402 | 0.000159 |
| ENSMUSG00000024731 |  | Ms4a10 | 222.0821055 | 17.39811282 | 3.649103148 | 2.91E-05 |
| ENSMUSG00000041857 |  | Oosp1 | 20.91322032 | 0 | 7.306020293 | 0.000366 |
| ENSMUSG00000024712 |  | Rfk | 14904.56162 | 3938.22147 | 1.919888293 | 9.31E-08 |
| ENSMUSG00000037847 |  | Nmrk1 | 229.0871401 | 645.1968567 | -1.494198992 | 1.05E-05 |
| ENSMUSG00000036192 |  | Rorb | 73.70343165 | 169.5782504 | -1.206662179 | 0.047286 |
| ENSMUSG00000058624 |  | Gda | 2777.779187 | 518.3832604 | 2.421081543 | 0.000322 |
| ENSMUSG00000052085 |  | Dock8 | 3643.820355 | 925.1766729 | 1.976867661 | 0.000181 |
| ENSMUSG00000024770 |  | Lipn | 45.78681856 | 1.611999042 | 4.617627217 | 0.012425 |
| ENSMUSG00000050370 |  | Ch25h | 19.02497309 | 194.979535 | -3.355698548 | 2.65E-07 |
| ENSMUSG00000041180 |  | Hectd2 | 225.4593562 | 566.8721416 | -1.329975624 | 0.001342 |
| ENSMUSG00000048612 |  | Myof | 1577.209151 | 3191.174422 | -1.016489942 | 6.66E-05 |
| ENSMUSG00000061132 |  | Blnk | 26.8235196 | 78.39330113 | -1.554614054 | 0.011212 |
| ENSMUSG00000025020 |  | Slit1 | 652.8325814 | 1958.189374 | -1.584811334 | 0.019046 |
| ENSMUSG00000025196 |  | Cpn1 | 0 | 21.48878647 | -20.64384567 | 0.000313 |
| ENSMUSG00000034765 |  | Dusp5 | 181.9007572 | 548.6499313 | -1.589941963 | 6.31E-05 |
| ENSMUSG00000033717 |  | Adra2a | 122.5288128 | 8.95907833 | 3.789957729 | 0.039386 |
| ENSMUSG00000024978 |  | Gpam | 17964.20748 | 8019.031717 | 1.163461889 | 0.045262 |
| ENSMUSG00000073295 |  | Nudt11 | 1258.206629 | 477.5842233 | 1.396130724 | 4.12E-05 |
| ENSMUSG00000062393 |  | Dgkk | 1668.806764 | 4391.980942 | -1.396119533 | 0.018601 |
| ENSMUSG00000054453 |  | Sytl5 | 221.4805755 | 692.4714937 | -1.644599177 | 4.57E-06 |
| ENSMUSG00000040229 |  | Gpr34 | 34.06963457 | 100.2189467 | -1.559840067 | 0.004219 |
| ENSMUSG00000037369 |  | Kdm6a | 4060.866699 | 1944.393494 | 1.060359701 | 2.29E-33 |
| ENSMUSG00000037341 |  | Slc9a7 | 613.3474477 | 1511.304422 | -1.301181593 | 2.89E-05 |
| ENSMUSG00000037347 |  | Chst7 | 86.97386151 | 40.7893177 | 1.078260262 | 0.022313 |
| ENSMUSG00000001986 |  | Gria3 | 2916.57192 | 7647.091402 | -1.390729419 | 0.000233 |
| ENSMUSG00000031104 |  | Rab33a | 409.2777721 | 122.1489733 | 1.740062067 | 0.000829 |
| ENSMUSG00000031111 |  | Igsf1 | 51.56665381 | 176.9323624 | -1.777652578 | 0.002149 |
| ENSMUSG00000031119 |  | Gpc4 | 6665.143717 | 2716.423693 | 1.294610649 | 9.89E-06 |
| ENSMUSG00000001964 |  | Emd | 2097.361714 | 4278.525427 | -1.028803727 | 4.52E-05 |
| ENSMUSG00000025056 |  | Nr0b1 | 332.7361551 | 81.64264046 | 2.025314048 | 0.0116 |
| ENSMUSG00000045103 |  | Dmd | 5126.081057 | 2314.939807 | 1.146287532 | 0.002532 |
| ENSMUSG00000034457 |  | Eda2r | 433.9739278 | 144.5202333 | 1.581774629 | 0.000678 |
| ENSMUSG00000031302 |  | Nlgn3 | 1471.267275 | 650.5861205 | 1.176889629 | 0.003657 |
| ENSMUSG00000051159 |  | Cited1 | 23.11641964 | 131.1687493 | -2.498465726 | 0.000864 |
| ENSMUSG00000086503 |  | Xist | 33752.65506 | 13.43585757 | 11.59416187 | 0 |
| ENSMUSG00000031264 |  | Btk | 38.56567851 | 99.22341414 | -1.359426029 | 0.001964 |
| ENSMUSG00000033436 |  | Armcx2 | 4327.578459 | 9273.422813 | -1.099654995 | 2.14E-06 |
| ENSMUSG00000031284 |  | Pak3 | 2674.753427 | 5875.290753 | -1.135374286 | 0.01072 |
| ENSMUSG00000044583 |  | Tlr7 | 275.3017329 | 576.7999536 | -1.067127499 | 0.027488 |
| ENSMUSG00000056673 |  | Kdm5d | 0 | 1802.595215 | -12.81294724 | 5.95E-24 |
| ENSMUSG00000069049 |  | Eif2s3y | 0 | 2158.634807 | -13.07387242 | 2.19E-19 |
| ENSMUSG00000068457 |  | Uty | 0 | 2046.610805 | -12.99772802 | 2.43E-24 |
| ENSMUSG00000069045 |  | Ddx3y | 0 | 8674.242086 | -15.08050599 | 1.26E-18 |
| ENSMUSG00000054446 |  | Cpa1 | 1.377556113 | 0 | 31.17797345 | 2.6E-06 |
| ENSMUSG00000092624 |  | Gm3654 | 10.85288259 | 0 | 6.360253343 | 0.01436 |
| ENSMUSG00000039488 |  | Cntn5 | 107.7230711 | 0 | 39.12753641 | 1.07E-15 |
| ENSMUSG00000042540 |  | Acot5 | 21.16237878 | 0 | 7.323597701 | 0.014478 |
| ENSMUSG00000046008 |  | Pnlip | 2.295926855 | 0 | 18.67517422 | 0.006753 |
|  |  |  |  |  |  |  |
